# Identifying potential palaeolithic artificial memory systems via Spatial statistics: Implications for the origin of quantification

**DOI:** 10.1007/s12520-025-02286-4

**Published:** 2025-07-23

**Authors:** Lloyd Austin Courtenay, Francesco d’Errico, Rafael Núñez, Damián E. Blasi

**Affiliations:** 1 CNRS, PACEA UMR 5199, Université de Bordeaux, Bât. B2, Allée Geoffroy Saint Hilaire, CS50023, Pessac, 33600 France; 2 Department d’Història I Història de l’Art, Universitat Rovira i Virgili, Avinguda de Catalunya 35, Tarragona, 43002 Spain; 3 Centre for Early Sapiens Behaviour (SapienCE), Department of Archaeology, Cultural Studies and Religion, University of Bergen, History, Bergen, 5020 Norway; 4 Eidgenössische Technische Hochschule Zürich (ETH), Rämistrasse 101, Zürich, 8092 Switzerland; 5 Center for Brain and Cognition, Pompeu Fabra University, Jaume I Building, Edifici Merce Rodereda, C/ de Ramon Trias Farcas 25, Barcelona, 08018 Spain; 6 Catalan Institute for Research and Advanced Studies (ICREA), Passeig Lluís Companys 23, Barcelona, 08010 Spain

**Keywords:** Notations, Cognition, Point pattern processes, Counting, Mobiliary Art

## Abstract

Artificial Memory Systems (AMSs) are tools that allow for the storage and retrieval of coded information beyond the physical body, ranging from computers and writing systems to tallying sticks. Current scientific knowledge suggests humans are the only species to manufacture and use these tools. While a number of artifacts dating back to the Middle Paleolithic have been considered to be early instances of AMS, conclusive and systematic evidence of this function is absent. Here we contrast the spatial distribution of markings on these potential early AMSs to other Paleolithic artifacts displaying butchery and ornamental marks, as well as ethnographically recorded cases of AMS. We find that both ethnographic and Upper Paleolithic AMSs are endowed with systematically different signatures that distinguish them from the other artifacts. These findings suggest that modern humans in at least Africa and Europe had sophisticated cognitive capabilities for information storage and retrieval, providing insights into the possible development of quantity-related cognition.

**Supplementary Information:**

The online version contains supplementary material available at 10.1007/s12520-025-02286-4.

## Introduction

*Homo sapiens* is the only extant species to jointly exhibit symbolic reference (Deacon 1997), full-blown language (Lieberman 1991), external representations (Kirsh 2010), active intentional teaching that requires mental state attribution (Matsuzawa 2001; Thornton and Raihani 2008), and to manifest massive cultural learning and cumulative cultural evolution (Tomasello 2015). Out of this unique composite set of traits, Artificial Memory Systems (AMSs) have emerged, which encompass devices specifically designed to record, store, transmit, and retrieve coded information beyond the physical body (d’Errico 1998; Johnson and Everett 2021) by means of symbolic resources and external representations. Based on current evidence, we may have been the only species to have unequivocally developed tools of this type (d’Errico et al. 2017), revolutionizing the way we accumulate and transmit knowledge via cultural evolution (Tomasello 2015). Some of the most striking examples of this innovation, are writing systems, the oldest examples of which date back to ≈ 3400 BCE in Mesopotamia, with other notable occurrences at 3300 BCE in Egypt, 1800 BCE in Greece, and 1400 BCE in China (Green 1981; Kahl 2001). Nevertheless, ethnographic evidence reveals that devices for storing coded information are, by no means, exclusive to so-called “complex” societies.

Some traditional societies of hunter-gatherers, fishermen, pastoralists, and horticulturists have also exhibited the use of AMSs to exchange messages, track time, navigate, record quantities, and as an aid memory in recalling prayers, rituals, and narrations (Marshack 1985; Genz 2016; Kelly 2020, 2024; Zavala, 2021). Even sub-groups from societies that eventually developed writing and numerical systems did not abandon the use of simpler AMSs, as is the case of the tally sticks used in England until the 17th century to collect taxes (Baxter 1989).

Ethnographic evidence suggests that AMSs may have existed in various parts of the world long before the emergence of ‘complex’ societies, writing, and numerical notational systems, and their use may have ancient roots. The questions of when and how these AMSs originated, and what impact their use had on the evolution of human cognition, remain largely unanswered. In order to answer this question, we investigate the possibility of identifying AMSs from the archaeological record, in the absence of functional evidence, through the use of new statistical tools and empirical evidence.

### Artificial memory systems in the Upper Paleolithic

Numerous artefacts have been documented in the late Middle (60 − 40 ka) and Upper Palaeolithic periods in Europe (42 ka -10 ka), as well as Early Later Stone Age sites in Africa (45 − 23 ka), presenting series of markings created using different techniques that have been interpreted as the earliest instances of AMSs (Marshack. 1972, 1991; d’Errico and Cacho 1994; d’Errico 1995, 1998). Although these interpretations have been repeatedly challenged (d’Errico, 1989; Robinson 1992), they have recently been revisited and bolstered by novel theoretical and analytical foundations (d’Errico 1995, 2002; d’Errico et al. 2017; Hayden 2021; Castelli 2023). On the one hand an examination of presently or historically known AMSs from diverse human cultures worldwide has identified four distinct factors for encoding information (d’Errico 1995; d’Errico et al. 2017): (i) the quantity of marks, (ii) the accumulation of marks over prolonged periods of time, (iii) the spatial distribution and arrangement of marks, and (iv) the morphology of the marks themselves. On the other hand, experimental programmes developed for the study of osteological artefacts bearing sequential markings have made promising advances in determining criteria to infer past behaviour from their microscopic features (d’Errico 1991, 1995, 1998; d’Errico and Cacho 1994; d’Errico et al. 2017; Bello et al. 2013). These advancements include determining the technique and type of tool used to produce marks, their chronology, whether they were made by the same or different tools and cutting edges, and whether they were accumulated over time.

In the present study, we therefore use the term AMS to designate a specific class of marked artefacts identified in both ethnographic and archaeological contexts that present the following features; sequences of incisions arranged with regular or semi-regular spacing; multiple identifiable changes in the cutting edge, tool or technique used to produce the incision or modification; spatial features implying intentional layout and organization of the markings, and; a lack of utilitarian shaping or edge modifications indicative of use. All of these variables have been formally defined in prior studies, but more importantly operationalized here, as the basis for inclusion in our analysis. It is true, however that this term already carries functional implications about their use, assuming that “memory” is the only thing they could have been used for. In recent work, Kelly (2024) advocated for a more neutral label such as Sequentially Marked Objects (SMOs) for the classification of these artefacts, presenting a more neutral descriptor of such artefacts. Nevertheless, in many of the cases of SMOs proposed by these authors, the difference with the current definition of an AMS does not necessarily consider the potential changes in how the marks were produced, used in the past to propose potential accumulation of markings over time. In the case of artefacts where marks have been observed to fulfill this criteria, we are more inclined to use the term AMS as the accumulation of markings and incisions over time is more consistent with the idea of incremental notation, such as that observed in record keeping. In any case, it cannot be denied that there is some potential bias in use of such term, and therefore we advise caution on its over-interpretation.

Applying the aforementioned criteria to Upper Palaeolithic objects featuring sequential markings has revealed that a number of them can tentatively be interpreted as potential AMSs (d’Errico and Cacho 1994; d’Errico 1995, 1998; d’Errico et al. 2017), as opposed to other types of human activity, including the production of artefacts with modifications to facilitate hafting (O’Connor et al., 2014; Zhang et al. 2016), possible musical instruments (Bradfield and Wurz 2020), adornments (Bello et al. 2013; Rodríguez-Hidalgo et al. 2019), use of bones as tools (Mallye et al. 2012; Doyon et al. 2023), or complex funerary practices (Bello et al. 2017). In parallel, developments in taphonomic analyses have refined criteria to distinguish between anthropogenic markings produced on bone, as opposed to bone surface modifications produced by natural factors (Olsen and Shipman 1988; Backwell, L.R., 2012; Courtenay et al. 2020).

A recent study applying these criteria found on two proposed AMSs that such devices may have already been in use in Africa before the onset of the Upper Palaeolithic in Europe, and possibly by Neanderthals (d’Errico et al. 2017). A first problem with the applied criteria, however, lies in the fact that the method of analysis used is particularly effective when potential prehistoric AMSs include accumulations of marks made with different tools and added over time. The interpretation of AMSs as objects bearing sequential marks that do not involve a change of tool may be based on evidence indicating, for example, that the marks result from a utilitarian activity. This could potentially include skin piercing, or to facilitate the attachment of tools, rendering the marks subsequently invisible. However, there will be cases where it will be difficult, in the absence of tool changes, to distinguish marks made for ornamental purposes from an AMS. Secondly, the applied approach requires in-depth, first-hand analysis of sometimes fragile archaeological objects held in numerous museums and research institutions. The results of the analyses have to be validated by experimental protocols adapted to the type of markings studied, which reduces the number of pieces examined to date.

In order to overcome these limitations, it is essential to complement existing methods with criteria derived from investigating the distinct features that perceptually separate AMSs from other markings. For instance, when examining the earliest examples of notational systems from ancient Mesopotamia, one can observe a distinct pattern of interconnected spatial organization (Green 1981). These systems are characterized not only by the individual shapes and forms of each character or pictogram and their associations, but also by the arrangement of signs into larger, coherent units, creating alignments. This spatial structuring transforms the object’s surface into a canvas of pre-conceptualised compartments that are crucial for the reader’s perception and retrieval of the coded information. Processing this visual information therefore becomes a task that involves symbolic thinking, pattern recognition and memory organization (Fitzgerald and Shanahan 2000; Carlson et al. 2013).

At present, the means in which we can describe this spatial organisation is primarily based on *in-visu* criteria, and the general visual inspection of artefacts. While this approach is valuable, it remains inherently subjective and limited in its ability to quantify patterning in a consistent and replicable way– particularly for facilitating both intra- and inter-artefact comparisons. In contrast, statistical toolkits exist that can quantify and empirically describe the distribution of features in space. From this perspective, it is possible to derive metrics that can be used not only to compare artifacts of different natures and uses but also to compare artifacts within the same group. This would provide another complementary method that can help study and investigate the different type of markings that can be observed on bone, and explore new means of describing these materials, opening new means for describing these materials and gaining insights into the emergence of symbolic and notational behaviour.

**Fig. 1:**
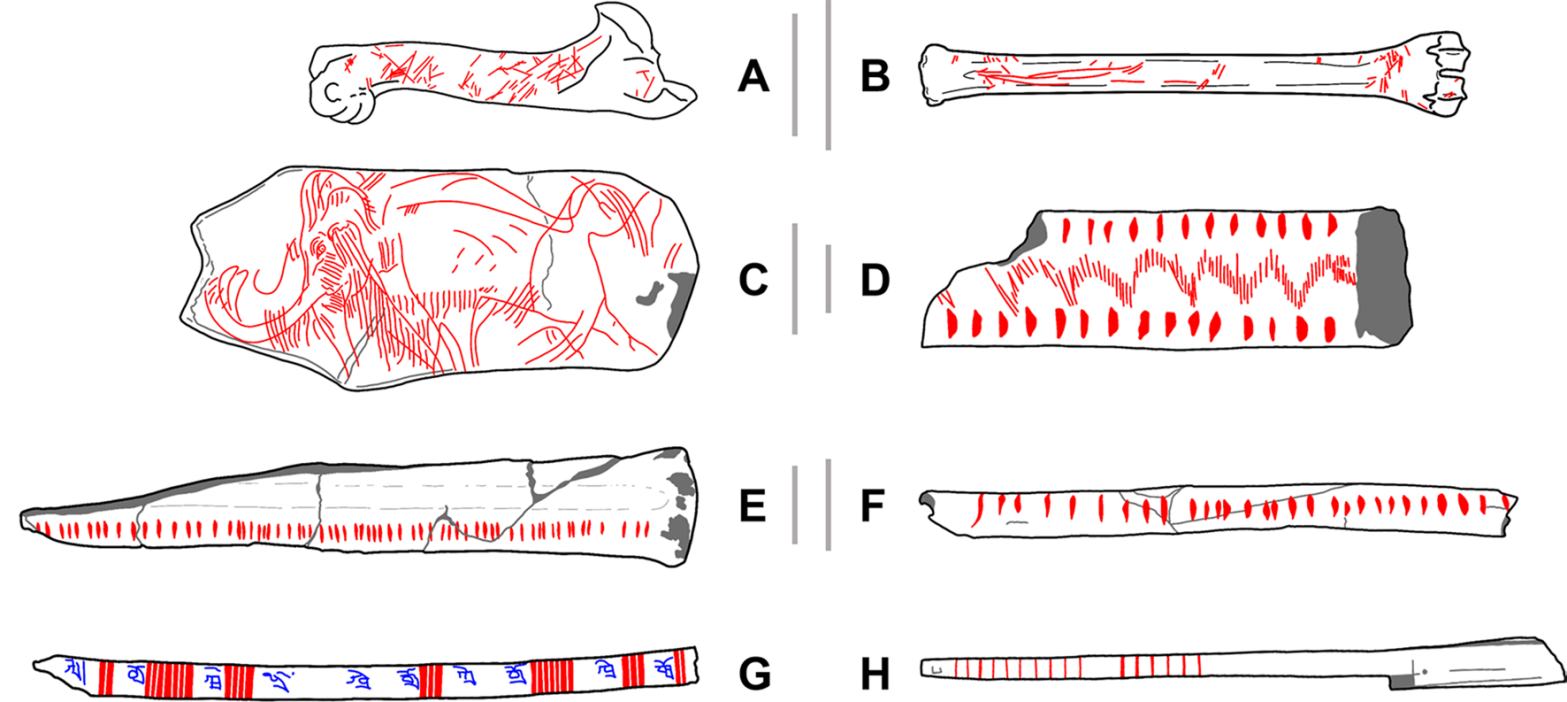
Examples of the different types of materials and engravings included in the present study, displaying (**A** & **B**) evidence of marks produced during modern butchery activities, (**C** & **D**) decorative engravings in the form of (**C**) zoomorphic and (**D**) geometric decorative motifs, (**E** & **F**) possible AMSs, and (**G** & **H**) known examples of AMSs. Red markings represent the modifications and engravings that were analysed for the purpose of this study. Scale bars are included if the scale of the artefact was documented. Scale bars A, B and C represent 5 cm, scale bar E represents 2 cm, and scale bars D and F represent 1 cm. See Supplementary Appendix A for more information on the artefacts and higher detailed illustrations

In this study we assess and quantify the degree of spatial organisation of different types of markings on bone with the aim of establishing criteria to discriminate them, thus contributing to the identification of prehistoric AMSs (Fig. 1). To achieve this goal we apply Point Pattern Process (PPP) to sequential and not sequential markings on ethnographic AMSs used to record and retrieve coded information (Fig. 1G and H), objects interpreted in the past as Palaeolithic AMSs on the basis of their technological analysis (Fig. 1E and F), Palaeolithic depictional representations on osseous material (Fig. 1C), Palaeolithic abstract, and arguably not notational, representations (Fig. 1D), and spatial patterning of cut marks produced accidentally during butchery experiments (Fig. 1A and B). Each artefact was encoded by taking the absolute position of each marking, as well as its orientation, in the context of the spatial window demarking the outline of the bone or artefact being studied. These coordinates were then studied using a number of techniques typical in spatial statistics. Through this, we attempt to quantify organisational and perceptual differences between these marking categories, with the aim of formally defining a methodology for the characterisation and identification of possible AMSs in the archaeological record. This thus provides a means of complementing traditional typological or visual assessments and helps reduce interpretive bias when relying solely on other more subjective criteria.

## Methods

### Sample

A total of 22 archaeological artefacts were analysed for the purpose of this analysis, presenting a total of up to 2840 markings, with a range from 7 to 584, and dating from between approximately 1.7 Ma to 6 ka BP. Three of these artefacts are bones with traces of butchery activities from the Lower, Middle and Upper Pleistocene. Nine bear depictional engraved representations. We acknowledge that visual categories such as “depictional” and “abstract” cannot be meaningfully assigned outside of their socio-cultural context. In this study, we use the term “depictional” simply to refer to artefacts that present more recognisably artistic or symbolic motifs, without implying a universal or culturally neutral classification (Gombrich 1984; Kelly et al. 2021). The total sample of previously hypothesized AMSs (*sensu* d’Errico and Cacho 1994; d’Errico 1995, 1998; d’Errico et al. 2017) include nine bones, dating between approximately 72 and 12 ka BP, accounting for a total of 534 marks. In addition to this sample, we have included examples of bones with traces inflicted by experimental butchery (see supplementary materials for details), including marks produced during defleshing and disarticulation activities on long bones, and tendon removal on metapodials. Considering the number of marks that would be typically found on a butchered bone, and the means in which the authors documented the spatial pattern of their experiments, we could only analyse these traces as a palimpsest of marks on an ideal bone outline. This disadvantage, however, is also advantageous as it provides us with a sufficient sample size in these cases for statistical evaluation of spatial patterns.

Our sample also includes nine documented examples of SMOs and AMSs consisting of elongated objects with sequential markings on sticks, dating from the 1st to the 20th Century CE. These examples are from North and Central America, to Africa, Europe, Asia, and Australasia. These artefacts display a total of 606 markings, with 15 to 341 marks per artefact. Here we have included; (1) two examples of documented native North and Central American calendars identified on wooden sticks and boards, used to document lunar cycles; (2) two aboriginal Australian message sticks, one used to document the number of days to travel from one place to another, and another containing a message regarding an invitation; (3) two tally sticks recovered from 13th Century CE England, documenting expenditures and taxes; (4) one tally stick from Tibet documenting deliveries of wheat and millet to a trading centre; and (5) two tally sticks from Angola documenting the number of days travelled.

While a much larger sample of known ethnographic SMOs and AMSs exist in the literature, especially in the case of the Australian message sticks, the current study intentionally focuses on smaller, well-documented subsets. This choice was made to demonstrate the methodology as a proof of concept, while avoiding potential statistical issues associated with oversampling certain artefact categories and undersampling others, which can introduce significant bias in multivariate analyses (Courtenay 2022). Future work, however, will have to expand the ethnographic sample in a more balanced and comprehensive manner, to understand the minute variations in different subclasses of SMOs.

Detailed drawings of each artefact (Fig. S1-S33), alongside descriptions and references are presented in Appendix A of the supplementary materials.

### Data collection

Drawings of the artefacts were digitised by means of a Wacom Intuos M graphics tablet (Wacom, Kazo, Japan). For each artefact, the coordinates denoting the 2D spatial window with anthropogenic modifications were recorded. In the case of osteological artefacts, this included the cortical perimeter or area of preserved cortical surface. This was then followed by the digitisation of the location of each of the markings observed on the artefact. The location was recorded depending on the type of mark. For linear marks, such as cut marks or incisions, they were digitised by recording the coordinates of the beginning and the end of each mark. Their general position was then calculated by taking the mid-point between these two coordinates. For circular marks, such as micro-incisions produced using percussive or boring techniques, the coordinates of a single point corresponding to the centre of the mark were recorded. For notches cut into the edge of the artefact, the deepest point of the mark was taken. For long linear marks with notable curvature, such as those observed in some decorations or depictions, multiple coordinates were recorded along the mark to comprehensively assess its location. Whenever possible, scale bars were also digitised so as to convert the pixel coordinates extracted from each drawing to scaled 2D coordinates in space.

For linear markings, additional information was extracted to analyse their orientation with respect to the primary axis of the bone or surface. This was performed by first computing the primary axis of the spatial window through the eigendecomposition of each outline. Eigenvectors and eigenvalues are then used to project the beginning and end coordinate of each mark onto the oriented transformed space of the spatial window. The orientation of the mark can then be evaluated through (Eq. 1);

1 $$\theta = \arctan \left( {{{y{\,_{begining}} - y{\,_{end}}} \over {x{\,_{begining}} - x{\,_{end}}}}} \right)$$

### Spatial analyses

Two powerful means of analysing the spatial patterns of Point Pattern Processes (PPPs) consider (1) the type of dispersion patterns that are present in a set of points (regular, random, or aggregated), and (2) the type of interaction between two sets of points (attraction, inhibition, or uncorrelation). As in most examples of statistical tests, spatial statistics attempts to accept or reject a null Hypothesis (*H*_*0*_) comparing an empirical set of observations to a theoretical model. In spatial statistics, the most common model for defining *H*_*0*_ is referred to as Complete Spatial Randomness (CSR), also referred to as a homogeneous *Poisson* point process (Ripley 1977; Diggle 1983). CSR describes a hypothetical PPP where points are distributed randomly and independently within a defined region. The outcome of the spatial distribution of points in one region will, under this premise, have no effect on the outcome of the spatial distribution of another set of points.

From this perspective, the alternative hypothesis, *H*_*a*_, would be that points are not distributed randomly in space, and that the position of one set of points does have an influence on the position of another. In light of this, PPP analyses consist in regarding dispersion patterns by comparing the empirical distribution of a set of points with CSR, and assessing the degree of either separation or attraction that occurs between points. The former type of PPP is typically referred to as a regular PPP, while the latter is known as clustering or agglomeration.

There are four main types of descriptive measures that can be used to characterise these patterns. These include Ripley’s *K* function, Besag’s *L* function, the Nearest-Neighbour distance *G* function, and the Empty Space *F* function (Besag 1977; Ripley 1977; Diggle 1983). Each method has advantages and disadvantages, and are usually used in unison so as to compare and contrast sources of information. The present analyses however have been based on the Empty Space *F* function, considering the separability between sets of marks in an AMS to be an important component of their definition (d’Errico 1998; d’Errico et al. 2017). This was complemented by the application of the Pair-Correlation Function (Ripley 1976; Illian et al. 2008), usually denominated by the lowercase symbol *g*, so as to provide a more complete overview of the spatial patterns at hand.

Both aforementioned methods consider the relationships between points at different scales and distances (*r*). *F(r)* can be considered the calculation of empty-space distances between points, and are presented as probability values with range [0, 1], giving the probability that there will be a point of the PPP lying within distance *r* from a given random point in the window. *g(r)*, on the other hand, assesses the relative density of points around a given point as a function of *r*, while these values are not bound between [0, 1]. For each of these analyses, the empirical distribution *F(r)* and *g(r)* are compared with the theoretical distribution of CSR in order to assess the type of distributions present. These comparisons are performed by plotting the theoretical *Poisson* line for each of the functions for each value of *r*, and seeing where the empirical line actually falls in comparison with this line. The interpretation of these results are different for *F(r)* and *g(r)* considering how values are computed. The *F(r)* function is interpreted as;

- If *F(r) > F*_*pois*_*(r)* then the PPP is characterised as presenting a distribution of regularly spaced points.
- If *F(r) < F*_*pois*_*(r)* then the PPP is characterised as presenting a distribution of points that are clustered together.
- If *F(r) = F*_*pois*_*(r)* then the PPP is characterised as fulfilling the criteria of CSR.

while *g(r)* is interpreted as;

- If g*(r) > g*_*pois*_*(r)* then the PPP is characterised as presenting a distribution of points that are clustered together.
- If *g(r) < g*_*pois*_*(r)* then the PPP is characterised as presenting a distribution of regularly spaced points.
- If *g(r) = g*_*pois*_*(r)* then the PPP is characterised as fulfilling the criteria of CSR.

The second type of analysis that can be performed on spatial data considers the interaction between different sets of points instead. In this case *H*_*0*_ is defined as the two sets of points having no spatial correlation with each-other, i.e. the position of one set of points has no influence on the position of another set of points, as would be assumed under CSR. *H*_*a*_, however, considers two separate scenarios; (1) the presence of one class of points inhibits the presence of another (inhibition), or (2) one type of point is more likely to cluster around another (attraction). By examining the spatial dependencies between distinct PPPs, cross-type analyses can provide an interesting perspective on how the presence of one PPP conditions the coexistence of another. This is performed by computing the expected number of points of type *j* lying within a distance *r* of a point of type *i*, and standardising these results by the intensity of points (Lotwick and Silverman 1982; Harkness and Isham 1983). The cross-type analysis used in this study is the *K*_*ij*_*(r)*, or *K-*cross analysis, and is interpreted as follows;

- If *K*_*ij*_*(r) > K*_*ij*, *pois*_*(r)* then a spatial dependency exists between the two PPPs (attraction).
- If *K*_*ij*_*(r) < K*_*ij*, *pois*_*(r)* then a repulsion between the two PPPs is occurring (inhibition).
- If *K*_*ij*_*(r) = K*_*ij*, *pois*_*(r)* then no correlation exists between the two PPPs.

For each of the PPPs *F(r)* and *g(r)* analyses were thus performed so as to characterise the spatial patterns of marks and modifications across the spatial window. *K*_*ij*_*(r)* calculations were additionally performed to either (1) characterise the relationship between two sets of different points in the same window (e.g. possible AMSs and other engravings or modifications found on the same surface, e.g. on artefacts from Solutré, La Marche and Tossal de la Roca), or (2) to assess the spatial relationship between different sets of marks included within an AMS (e.g. different sets and subsets in objects, e.g. from La Marche and Labattut).

These statistical approaches can be problematic due to biases inherent to spatial data. A potential bias relevant here is the border bias. This arises when studying point patterns that occur near the boundary of the study area, i.e. the edge or border. Estimations may be influenced in this case by our inability to detect points that occur outside the limits of our spatial window (Ripley 1988; Badeley and Gill, 1993). Likewise, points lying closer to the edge have a limited buffer zone, implying that the number of neighbours a point can have will be more restricted as opposed to points lying farther away from the edge. This is particularly relevant in studies of bone surface modifications occurring on incomplete and damaged specimens, in which our ability to document the full extent of a PPP across the original spatial window is reduced. To correct for this, the *F(r)* and *K*_*ij*_*(r)* functions were adjusted using the “reduced sample” estimator (Baddeley and Gill 1993; Baddeley, 1998), and *g(r)* used an isotropic correction (Ripley 1977). In addition, *g(r)* is typically estimated using a kernel smoothing function. For the present study, we used the Epanechnikov kernel (Stoyan and Stoyan 1994), applying a constant of 0.15 for the definition and correction of intensity (*sensu* Illian et al. 2008). The combined application of these methods is considered as a way to substantially minimize the border bias.

An additional source of error in analyses of this type is the parametric nature of these functions. All three functions *F(r)*,* g(r)*, and *K*_*ij*_*(r)*, assume the PPP to be stationary, i.e. the nature of the distribution is the same across the entire window and, as a consequence, the results are not conditioned by the arbitrary point where *r* is computed from. To provide non-parametric alternatives for each of these statistics, corrections are included that take into account the intensity of points in certain areas (Lieshout 2011), thus requiring the definition of an intensity parameter λ. When robust estimators of *F(r)*,* g(r)*, and *K*_*ij*_*(r)* were required, the present study automatically calculated λ using a leave-one-out kernel smoothing technique, *sensu* Baddeley et al. (2016). The nature of a PPP’s homogeneity was assessed using the χ^2^ test for homogeneity based on quadrat counts. In this case, *H*_*0*_ assumes that, after dividing the window into quadrats, the number of points in each section is roughly the same. For this purpose, the window is divided into sub-regions, and the number of points in each region is compared with the expected number of points given the intensity of points across the window after 10,000 permutations using Monte Carlo algorithms.

As pointed out by Greig-Smith (1952) and Mead (1974), the results of χ^2^ testing are directly related with the size of the quadrats chosen. This implies that care must be taken when choosing the size of the quadrat, which should not be too large or too small. For this purpose, a number of simulations were performed on different windows to find an optimal quadrat size. The relationship between the χ^2^ test statistic and the size in cm^2^ of each window was found to present an exponential relationship (Fig. S76), with the general consensus revealing a window size of between 6cm^2^ and 8cm^2^ to be the most suitable for χ^2^ calculations. In light of this, the quadrats along the *x* and *y* axis of each bone were calculated in a way that ensured the quadrats had an equal square area of ≈ 6-8cm^2^, while quadrat sizes for pieces that were considerably smaller were recomputed for those specific case-studies separately. For the known notation systems where scales were not available, quadrat sizes were roughly estimated to ensure they were square and roughly the same size as for the archeological finds. Supplementary Table 1 shows the results of these analyses for each of the artefacts studied.

Finally, for the evaluation of *F(r)*, *g(r)* and *K*_*ij*_*(r)* results with respect to the *Poisson* curve, the present study proposes the following means to evaluate results beyond simple visual inspection of curves: we computed the Area Under the Poisson Curve (AUPC), and the Area Over the Poisson Curve (AOPC), considering the signed difference (ϵ) for each value of *r* between the empirical and theoretical curves. Following this we can compute and quantify the degree of *F(r)* that falls both under and over the *Poisson* line using;

2 $$\eqalign{ AOPC & = {h \over 2}\left( {F\left( {r_0^ + } \right) + 2\int_{r_1^ + }^{r_{n - 1}^ + } {F\left( {{r^ + }} \right)d{r^ + } + } F\left( {r_n^ + } \right)} \right) \cr & = {h \over 2}\left( {F\left( {r_0^ + } \right) + 2\sum\limits_{i = 1}^n {F\left( {r_i^ + } \right)} + F\left( {r_n^ + } \right)} \right) \cr} $$

where *r*^*+*^ indicates only those values of ϵ that are positive, *n* is the number of *r* values, and *h* is the width of the segment under the curve using the composite trapezoidal rule, i.e. *h = (r*_*n-1*_ *– r*_*1*_*)/n*. For AUPC, then *r*^*+*^ is substituted by *r*^*-*^, while *F(r*^*+*^*)* values are substituted by *|F(r*^*-*^*)|*. So as to ensure the integrated values are invariant to the size of the spatial window, and thus comparable across specimens, *r* values are scaled to fall between 0 and 1. In cases where the computed empirical values do not fall between 0 and 1 (namely for *g(r)* and *K*_*ij*_*(r)*), then these values are also scaled to fall between 0 and 1.

All statistical analyses were performed using the R programming language (v.4.3), with the help of the spatstat (v.3.0) library (Baddeley et al. 2016).

### Orientation pattern analyses

Where marks’ orientation were measured, these values were used to calculate two metrics describing the general statistical circular tendencies of values, followed by a calculation of preferential orientation. For descriptive statistics the first step considered calculating the central orientation of marks with respect to the main axis of the bone. This was typically performed calculating the circular mean (θ), and was followed by a calculation of the Sample Circular Variance (*v*). *v* values have a range *v* ∈ [0, 1], with the lower limits of this range indicating a small amount and the latter indicating a large amount of variance around the circle. θ was initially calculated in radians. However, for interpretability reasons these values were simply converted to degrees by θº = θ^r^(π/180). In subsequent multivariate analyses where θ values were compared with other variables not lying within a circular topological space, values were projected into Euclidean space by taking θ values in radians and calculating θ_lin_ = cos(θ) + sin(θ). Calculation of preferential orientation were then performed according to the Rayleigh test (Watson and Williams 1956). For the Rayleigh test we assume *H*_*0*_ to indicate θ values to be uniformly distributed around the circle. Finally, when comparisons could be made between two sets of angles we additionally performed a randomised version of the Mardia-Watson-Wheeler test (Wheeler and Watson 1964), with *H*_*0*_ assuming two sets of angles belong to the same distribution.

### Multivariate statistical evaluation and ordination of results

Once spatial and orientation patterns had been recorded for each of the artefacts, multivariate analyses were performed to inspect the relationships between artefacts and understand the spatial characteristics of different types of markings.

First we used Principal Components Analysis (PCA) as an ordination technique, with the goal of efficiently visualizing the four different variables: AUPC or *F(r)*^*-*^, AOPC or *F(r)*^*+*^, θ_*lin*_ and *v*. Since all of the variables are unitless, and generally fall within a comparable scale, the use of the covariance matrix was considered sufficient for the computation of PC scores. Most of the variables, with the exception of θ_*lin*_, are theoretically bound within the same range of [0, 1]. The variable θ_*lin*_, while not strictly bounded, is also unitless, and it’s linear transformation is constrained within approximately [-2, 2], due to the angular definition [0, 2π] in radians. However, since the data follow a von Mises distribution with values concentrated within a single hemisphere ∈ [0, π], the effective spread and scale of θ_*lin*_ remains limited, making it broadly comparable to the other variables.

Needless to say, it is important to emphasize that PCA was used solely for the purpose of exploratory analysis, and not as the basis for formal analysis or interpretation. We have also refrained from using ordination techniques that are more oriented towards classification tasks, such as the visualisation of Canonical Variates or between-group Principal Components (Albrecht 1992; Mitteroecker and Bookstein 2011). While these methods can be useful, they have recently been subjected to significant scrutiny, particularly concerning their algebraic assumptions and epistemological limitations (Bookstein 2017, 2019). In particular, these tests assume a prior-knowledge of our data’s structure and distribution, while the objective of the current study is to let the data speak for itself, and see how patterns emerge based on the underlying mathematical and statistical properties of our data, as opposed to pre-conceptualised understanding of the materials at hand (Courtenay 2022, 2023; Courtenay et al. 2024). In other words, we have chosen an approach that allows patterns to emerge from the intrinsic statistical properties of the data without predefined expectations.

Given the above limitations and epistemological caveats, a more specific unsupervised pattern recognition approach was also used. Here we employed hierarchical clustering analyses by means of the Unweighted Pair-Group Method with Arithmetic Mean (UPGMA), fit on dissimilarity metrics measured using Euclidean distances. Subsequent evaluation of clustering outcomes entailed two key metrics; the cophenetic correlation coefficient, measuring the fidelity of dendogram representations of the original pairwise distances among data points (Sokal and Rohlf 1962), as well as the Adjusted Mutual Information (AMI) criterion, a measure used to assess the agreement between the clusters and the original labels (Kraskov et al. 2005). To ensure an optimal interpretation of these results, and validate our models, multiple theoretical simulations were executed to provide expected AMI values and correlation coefficients under multiple different hypotheses (see Supplementary Appendix E).

Finally, a Multivariate Analysis of Variance (MANOVA) was then performed, using the non-parametric Wilk’s Lambda test statistic, so as to assess statistical differences between groups based on these 4 variables. MANOVA results were permuted 1000 times to provide more robust estimations in the calculation of *p*-values.

Throughout this study, *p*-values have been evaluated considering *p* < 0.003 to be a threshold of rejecting *H*_*0*_. This was chosen considering the 28.9% probability of Type I statistical errors given the use of *p* < 0.05, using traditional prior probabilities of 0.5 (Colquhoun 2019). This is additionally associated with posterior odds of 1:1.22 in favour of *H*_*a*_. *p* < 0.003 (*sensu* Courtenay et al. 2021; Courtenay 2024), on the other hand, has a much more robust 4.5% probability of Type I statistical errors, with posterior odds of 1:10.6 in favour of the *H*_*a*_. For transparency, all *p*-values were reported alongside the probability of being a Type I statistical error, known as the False Positive Risk (FPR) value (Colquhoun 2019). All FPR values were computed using prior probabilities of 0.2, assuming a worst case scenario that the analysed samples are insufficient to withdraw firm conclusions (∴ *p* < 0.003, FPR < 15.9%).

## Results

### Characterising anthropogenic bone surface modifications from the Pleistocene

**Fig. 2:**
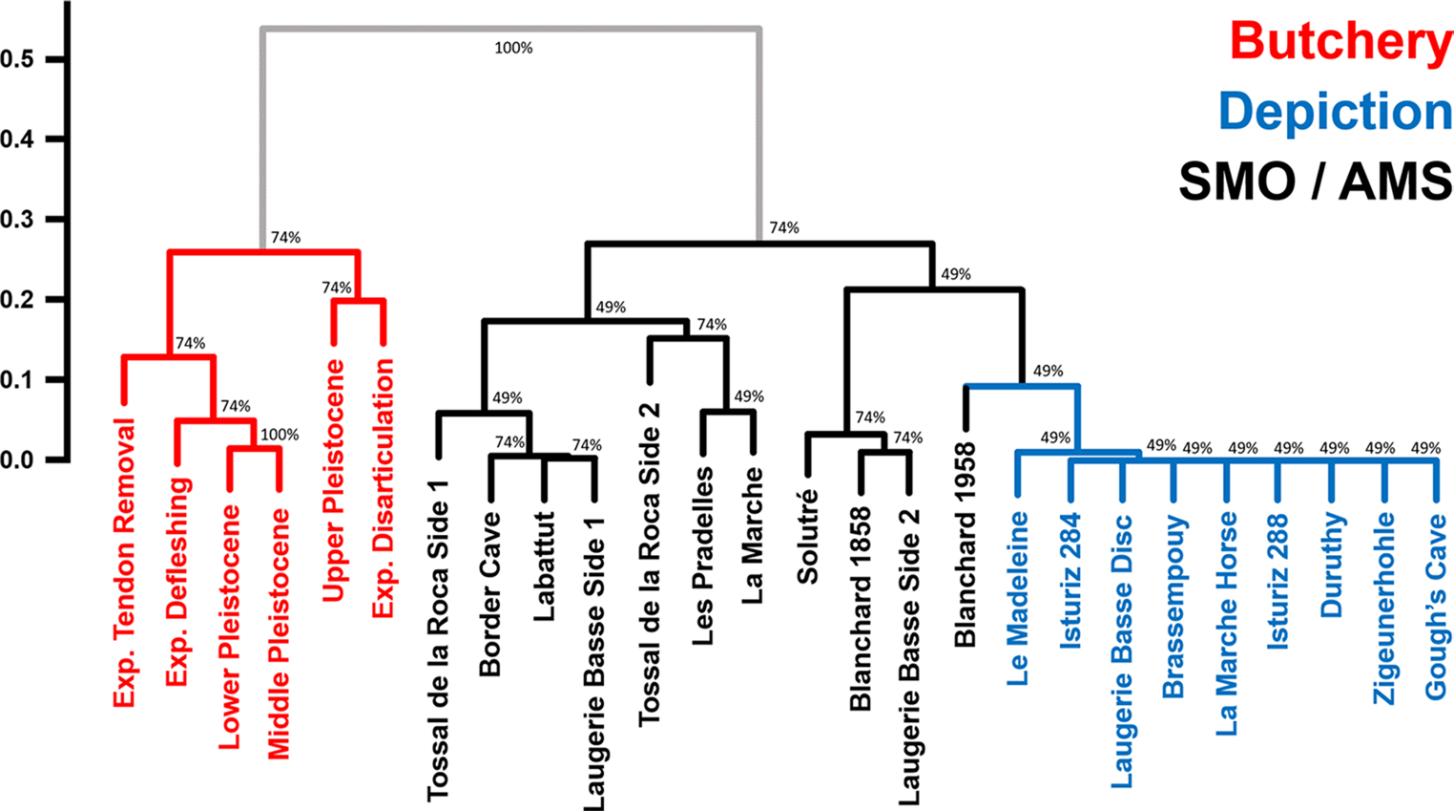
Hierarchical clustering results using the Unweighted Pair Group Method with Arithmetic Mean (UPGMA) algorithm. Clusters were calculated using the Euclidean distance between each individual using pure spatial statistical variables, such as the Area Under and the Area Over the Poisson Curve (AUPC and AOPC) values calculated using the Empty-Space F(r) function. Exp. = Experimental; AMS: Artificial Memory Systems

Upon examining the spatial distribution of marks on the analysed objects, distinct trends emerge, revealing specific spatial arrangements corresponding to various activities. Butchery traces indicates a pronounced tendency towards spatial clustering (Fig. S34-S39), depictional or abstract motifs demonstrate a trend towards Complete Spatial Randomness (CSR) (Fig. S40-S48), while the potential archaeological AMSs feature a tendency towards regular spatial patterns (Fig. S49-S67). Hierarchical clustering analysis reveals a clear separation between these groups based solely on spatial variables (Fig. 2), supported by a cophenetic correlation coefficient of 0.89, and Adjusted Mutual Information (AMI) criterion of 0.68, both falling within the range of what would be expected for non-overlapping cluster groups (Supplementary Appendix E). In general, all SMOs / potential AMSs fall away from other types of engraved and marked artefacts, with the exception of the Blanchard D38.23.1958 rib, which fall closer to depictions. Nevertheless, when the relationship between *F(r)*^*+*^ and *F(r)*^*-*^ variables (Fig. S78) is visualized, it appears that objects bearing depictions or abstract patterns generally occupy a reduced region of feature space, and appear clearly separable from any of the potential AMSs examined.

**Fig. 3:**
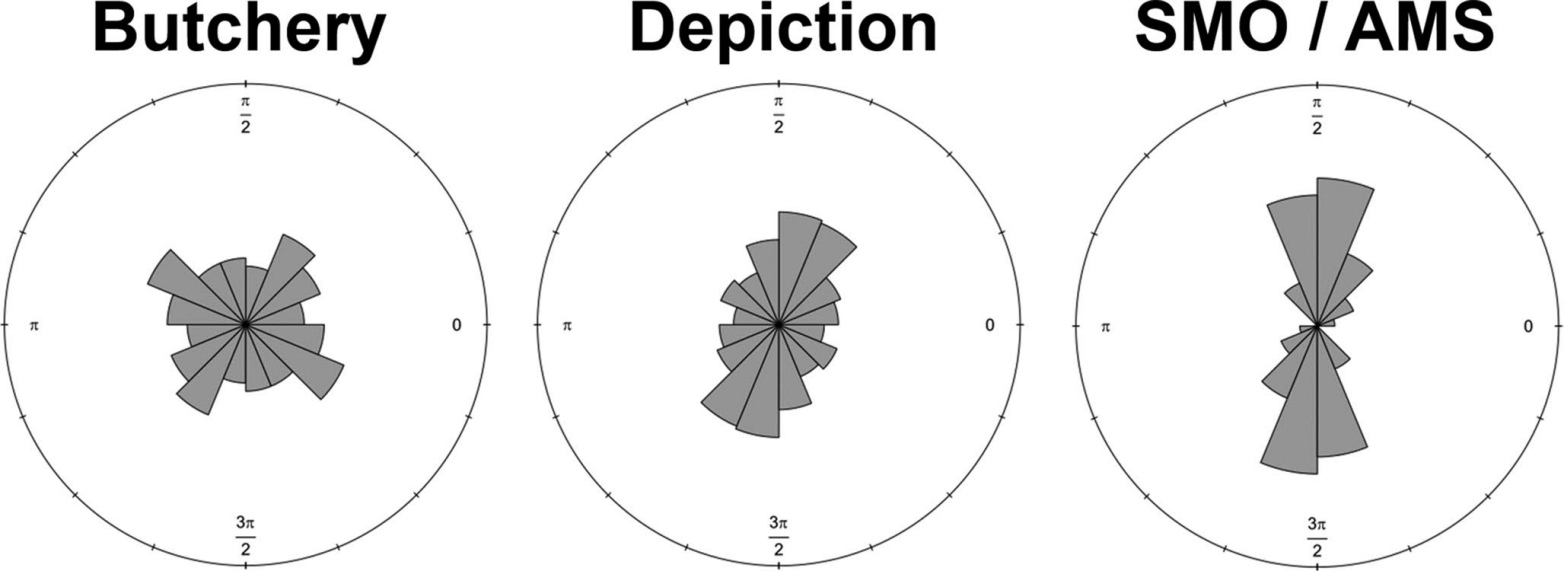
Rose diagrams representing the distribution of angles (in radians) according to the main axis of the bone on which the marks occur

Distinct trends also emerge when assessing mark orientations (Fig. 3). Butchery traces exhibit the highest variability (central *v* = 0.045; Table 1) with generally oblique angles (≈ 40º) to the primary axis of the bone. Depictional and abstract motifs present a slightly lower degree of variation (*v* = 0.048), with a trend towards a less marked oblique general orientation (≈ 60º). SMOs / AMSs are characterised by the lowest degree of variation (*v* = 0.01) with a predominant orientation almost perpendicular to the main axis of the bone (≈ 80º). These patterns are additionally reflected in results from the Rayleigh test: while all samples are found to present notable preferential orientations (Table S2; *p* < 2.8 × 10^–16^, FPR < 2.8 × 10^–12^%), the test statistic for each artefact are thus higher for AMSs (*z* ≈ 0.988) than they are for butchery (*z* ≈ 0.969) or depictions samples (*z* ≈ 0.96).

**Table 1:** Values for the variables taken into account in the analysis of pleistocene and experimental bones bearing marks. area under the Poisson curve (AUPC, i.e. F(r)^-^) and area over the Poisson curve (AOPC, i.e. F(r)^+^) are derived from the Empty-Space F(r) function, θ values indicate the central tendency of mark orientations (where present) in degrees (º), the linear transformation of these values θ_lin_, and the sample circular variance (v). Exp. = Experimental

Name	Sample	AUPC	AOPC	θ	θ_lin_	v
Exp. Defleshing	Butchery	0.570	0.004	41.0	1.411	0.042
Exp. Disarticulation	Butchery	0.430	0.000	67.2	1.309	0.016
Exp. Tendon Removal	Butchery	0.729	0.000	47.4	1.413	0.120
Lower Pleistocene	Butchery	0.612	0.000	25.9	1.336	0.055
Middle Pleistocene	Butchery	0.626	0.000	18.5	1.266	0.019
Upper Pleistocene	Butchery	0.382	0.193	42.9	1.413	0.015
Le Madeleine	Decoration	3.6 × 10^− 4^	0.008	59.6	1.369	0.099
La Marche Horse	Decoration	3.7 × 10^− 4^	2.8 × 10^–11^	29.8	1.364	0.065
Laugerie Basse Disc	Decoration	8.4 × 10^− 4^	7.6 × 10^− 9^	57.0	1.384	0.083
Zigeunerhohle	Decoration	3.2 × 10^–12^	0.000	46.9	1.413	0.049
Brassempouy	Decoration	0.001	8.4 × 10^− 5^	69.2	1.29	0.035
Isturitz 288	Decoration	2.1 × 10^− 9^	1.6 × 10^− 4^	81.9	1.131	0.018
Isturitz 284	Decoration	3.8 × 10^− 9^	0.002	74.9	1.226	0.036
Duruthy	Decoration	2.8 × 10^–13^	4.9 × 10^− 8^	60.9	1.36	0.027
Gough’s Cave	Decoration	0.000	0.000	78.2	1.184	0.016
Blanchard 1858	SMO/AMS	0.198	0.000	87.1	1.049	3.2 × 10^− 4^
Solutré	SMO/AMS	0.234	0.006	75.2	1.223	0.012
Labattut	SMO/AMS	0.000	0.179	86.9	1.053	0.001
Les Pradelles	SMO/AMS	1.2 × 10^− 4^	0.395	77.2	1.197	0.016
Border Cave	SMO/AMS	0.000	0.183	84.0	1.1	0.006
Tossal de la Roca (1)	SMO/AMS	5.0 × 10^− 4^	0.238	73.5	1.243	0.016
Tossal de la Roca (2)	SMO/AMS	0.140	0.315	64.9	1.33	0.040
Blanchard 1958	SMO/AMS	1.9 × 10^–18^	0.093			
La Marche	SMO/AMS	2.4 × 10^− 8^	0.336			
Laugerie Basse (1)	SMO/AMS	1.3 × 10^− 7^	0.178			
Laugerie Basse (2)	SMO/AMS	0.207	0.000			

**Fig. 4:**
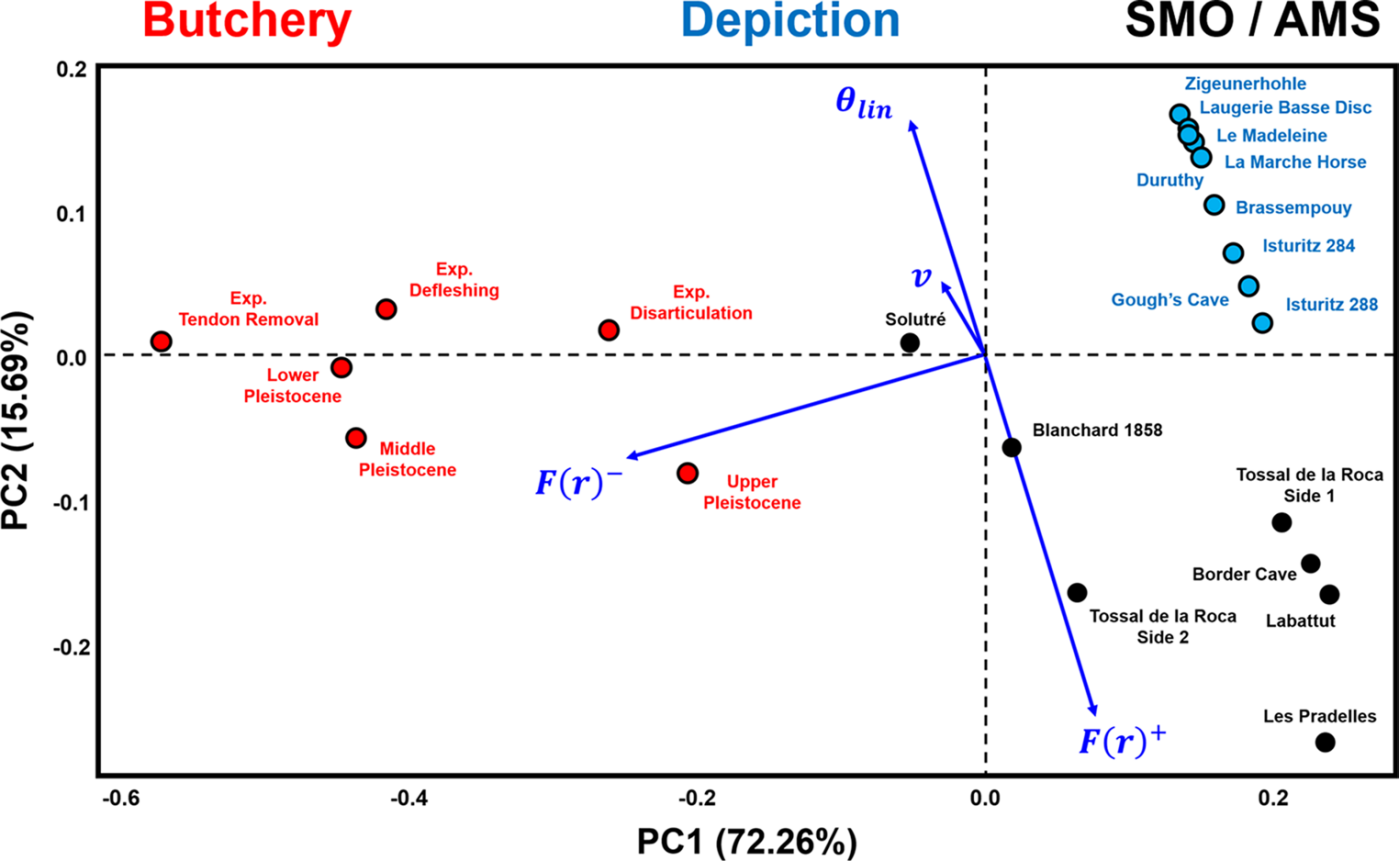
Principal Components Analysis biplot of three categories of markings, analysed by spatial and orientation related variables. Area Under the Poisson Curve values are represented by the symbol F(r). Area Over the Poisson Curve values are represented by the symbol F(r)^+^. θ_lin_ refers to the linear transformation of central tendency values, while v is the sample circular variance. Exp. = Experimental

The combination of variables describing both spatial and orientation patterns (Fig. 4) reaffirms the distinct separation between different types of markings based on spatial variables alone. Each possible AMS category exhibits a regular spatial distribution with perpendicular marks concentrated around the circular mean. In contrast, butchery traces present a much larger variability characterised by *F(r)* curves that fall below the *Poisson* line, with higher sample circular variability. Depictional representations, including geometric and zoomorphic motifs, occupy a restricted portion of the PCA feature space, mostly determined by low *F(r)* values. Interestingly, although geometric engravings found on artefacts such as the Isturitz lissoirs and the Gough’s Cave human radius, plot at the very limit of their category distribution, a clear divide separate them from artefacts interpreted as bearing AMSs.

In sum, our results reveal SMOs, or artefacts bearing markings interpreted as AMSs, to be characterised by a distinct spatial organization. This is especially evident in the case of the La Marche incised antler. Its surface is divided into distinct sections– one dedicated to engraved depictions, the other devoted to notational markings (Fig. S57). Limited associations are detected between these two marking categories, indicating a clear premeditated organisation of the available space. The more compelling evidence for the deliberate nature of this choice is seen through cross-type interactions between sets along Side 1 (Fig. S60), a feature that is also observable on both faces of the Laugerie Basse spatula (Fig. S63). Another example of this is found on the Tossal de la Roca Pendant, where cross-type interactions show a spatial attraction between the baseline markings produced during the preparation of the surface, so as to act as guidelines for the later recording of information (d’Errico and Cacho 1994).

A closer examination of the potential AMS group reveals that the Solutré and Blanchard D38.23.1858 ribs stand out as relatively anomalous instances. Results from clustering analysis (Fig. S79) also indicate that these findings are somewhat distinct compared to other SMOs / AMSs. This result is consistent with d’Errico’s observations (d’Errico 1998) according to which the Blanchard rib may have also been used in knapping activities and not necessarily or uniquely as an AMS. The Solutré rib does exhibit an arrangement of marks deviating from the usual clear and regular point patterns seen on other artifacts interpreted as AMSs. This object shows a tendency for the marks to cluster together, making the “regular” point patterns much less clear compared to other artifacts. These patterns are highlighted to a different degree when considering the *g(r)* curves (Fig. S49 & S51), presenting a clearer tendency towards clustering at points falling in the range of 0.1 and 0.3 mm from each other, with the example of Solutré extending this pattern to even 0.75 mm distances. It is clear here that this is conditioned by the relative concentration of the Solutré engravings, leaving a considerable amount of space across the rest of the artefact, quite possibly for more room to add incisions, or to make room for the more decorative incisions that are located towards the other end of the rib.

### Comparison with known examples of SMOs and AMSs

Comparisons between historically known SMOs and AMSs, as well as Pleistocene objects interpreted as such, reveal striking similarities in spatial organization and orientation patterning. Irrespectively of their actual function (calendars, message stick, tally sticks), these objects bear regular spatial distributions characterised by high *F(r)*^*+*^ values (Table 2), and highly preferential orientation patterns (Table S2; *z* ≈ 0.999, *p* < 2.8 × 10^–16^, FPR < 2.8 × 10^–12^%) of minute variation (*v* = 0.001), almost perfectly perpendicular (≈ 84º) to the main axis of the artefact.

**Table 2:** Values for the variables considered when analysing historically known artificial memory systems and sequentially marked objects. Variables include the area under the Poisson curve (AUPC) and area over the Poisson curve (AOPC) values derived from the Empty-Space F(r) function, θ values indicating the central tendency of mark orientations (where present) in degrees, the linear transformation of these values θ_lin_, and finally the sample circular variance (v)

Name	Sample	AUPC	AOPC	θ	θ_lin_	v
Winnebago	Calendar	0.000	0.118	81.3	1.140	0.023
Chamula	Calendar	0.043	0.223	74.5	1.231	0.004
Aboriginal Australian	Message	0.000	0.099	88.6	1.024	1.1 × 10^− 4^
Medieval (English)	Tally	0.000	0.182	84.7	1.088	0.003
Medieval (Jewish)	Tally	0.000	0.142	88.4	1.028	2.3 × 10^− 4^
Mirān	Tally	0.034	0.128	87.6	1.042	4.8 × 10^− 4^
Muacapenda	Tally	7.9 × 10^− 6^	0.165	86.8	1.055	0.001
Aboriginal Australian 2	Message	0.000	0.141			
Muatchondo	Tally	0.000	0.145			

**Fig. 5:**
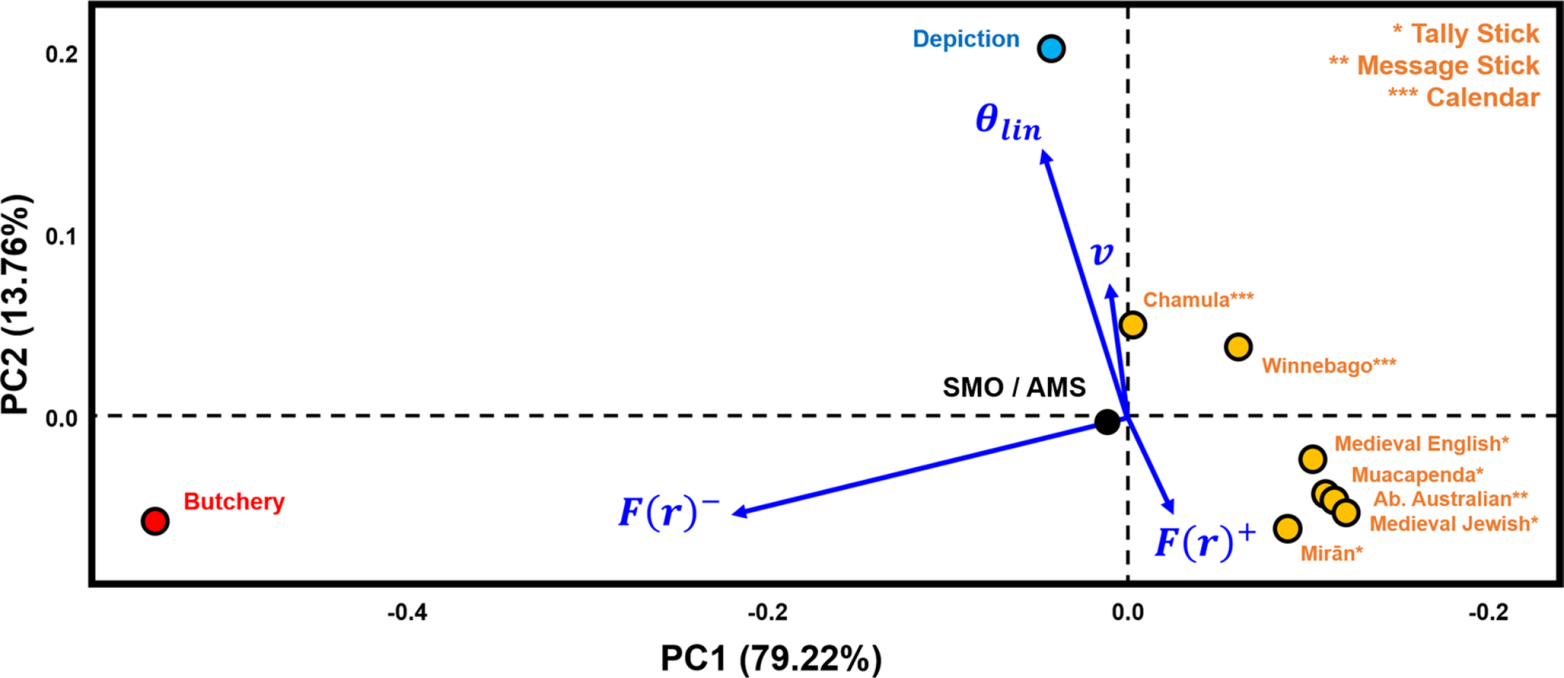
Principal Components Analysis biplot comparing examples of known Artificial Memory Systems and Sequentiall Marked Objects with the median values of three categories of markings on Palaeolithic and experimental artefacts. Area Under the Poisson Curve values are represented by the symbol F(r)^-^, while Area Over the Poisson Curve values are represented by the symbol F(r)^+^. θ_lin_ refers to the linear transformation of central tendency values, while v is the sample circular variance

**Fig. 6:**
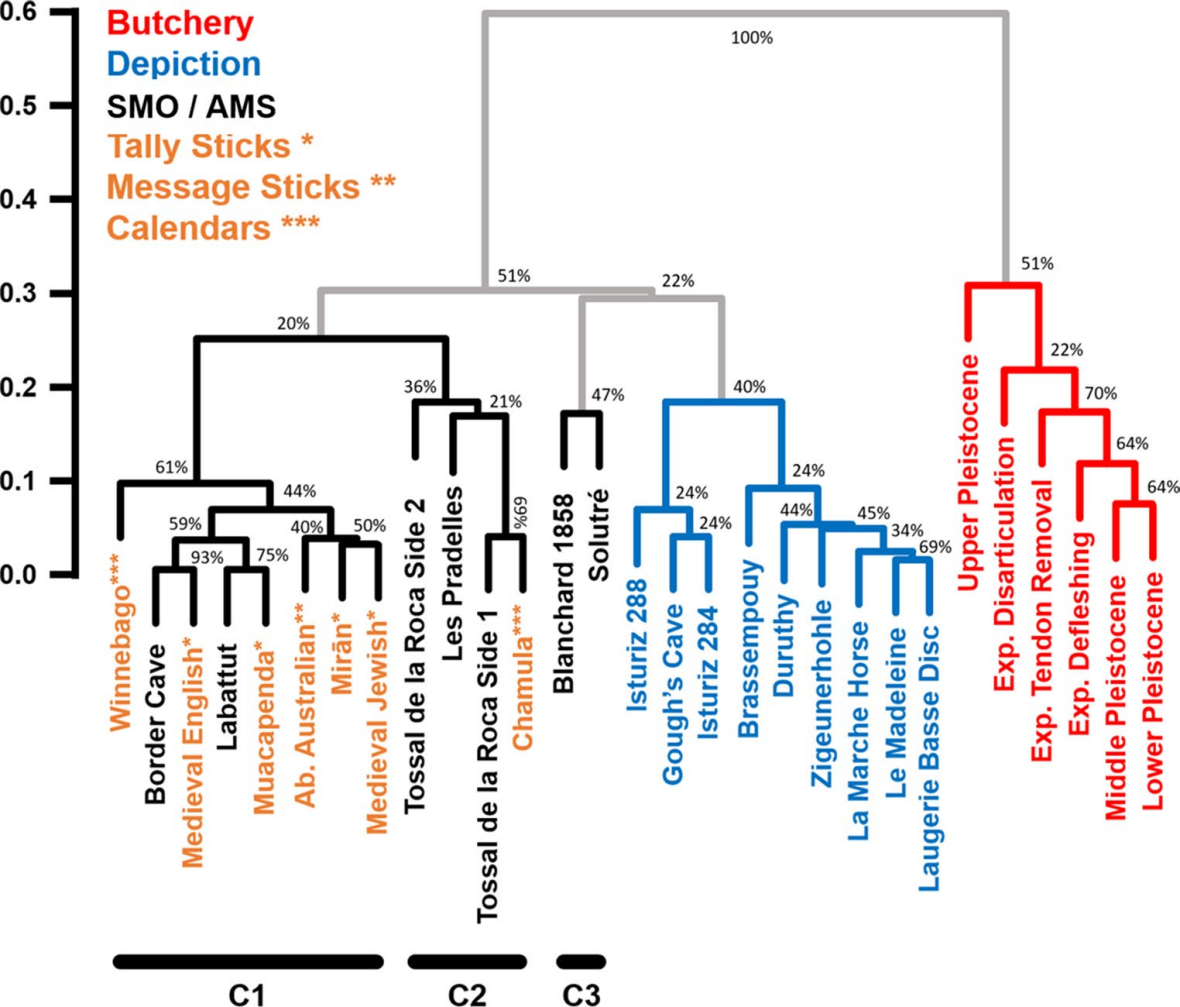
Hierarchical clustering of different categories of markings using the Unweighted Pair Group Method with Arithmetic Mean (UPGMA) algorithm. Clusters were calculated using the Euclidean distance between each individual using pure spatial statistical variables, the Area Under and the Area Over the Poisson Curve (AUPC and AOPC) values calculated using the Empty-Space F(r) function. C1, C2 and C3 identify the 3 main clusters detected through the analysis related with AMSs. Exp. = Experimental. Ab. = Aboriginal

Multivariate analyses reveal that ethnographic SMO / AMS cluster with the prehistoric examples (Fig. 5 & S80), an observation confirmed by hierarchical clustering analysis (Fig. 6). These results are additionally supported by a cophenetic correlation coefficient of 0.89 and AMI of 0.72, which fall in our simulated range of what would be expected if three distinct clusters were to exist (Supplementary Appendix E). UPGMA results identify three main clusters of AMSs, two of which are represented by both ethnographic and Pleistocene artefacts (Fig. 6: C1 & C2), and a third cluster (C3) which confirms the slightly different nature of the Blanchard 1858 and Solutré ribs in comparison with other potential AMSs. Each of the recorded divergences between groups are reinforced by permuted MANOVA results (Table 3), with AMSs presenting notable differences with both butchery and artistic samples. The probability of this observation being a Type I statistical error accounting to 8.07%. In contrast, no differences are observed between ethnographic and archaeological SMOs / AMSs. These analyses show that ethnographic SMOs / AMSs mix perfectly with those from the Pleistocene. The baboon fibula from Border Cave and the reindeer metapodial from Labattut, in particular, closely match patterns seen on various tally sticks from both Africa and Europe.

**Table 3:** Multivariate analyses of variance (MANOVA) results using wilk’s lambda as the test statistic. p-Values and F values were computed using 1000 permutations so as to adjust for possible errors due to small sample sizes. False positive risk (FPR) values, reported as percentages, were additionally calculated to adjust for possible sample size errors, using 0.2 priors in favour of the null hypothesis

		Palaeolithic SMO/AMS	Depiction	Butchery
Depiction	*F*	23.5		
	*p*	0.0012		
	FPR	8.07		
Butchery	*F*	18.6	116.3	
	*p*	0.0012	0.0012	
	FPR	8.07	8.07	
Modern SMO/AMS	*F*	1.78	54.3	80
	*p*	0.195	0.0012	0.0012
	FPR	77.6	8.07	8.07

**Fig. 7:**
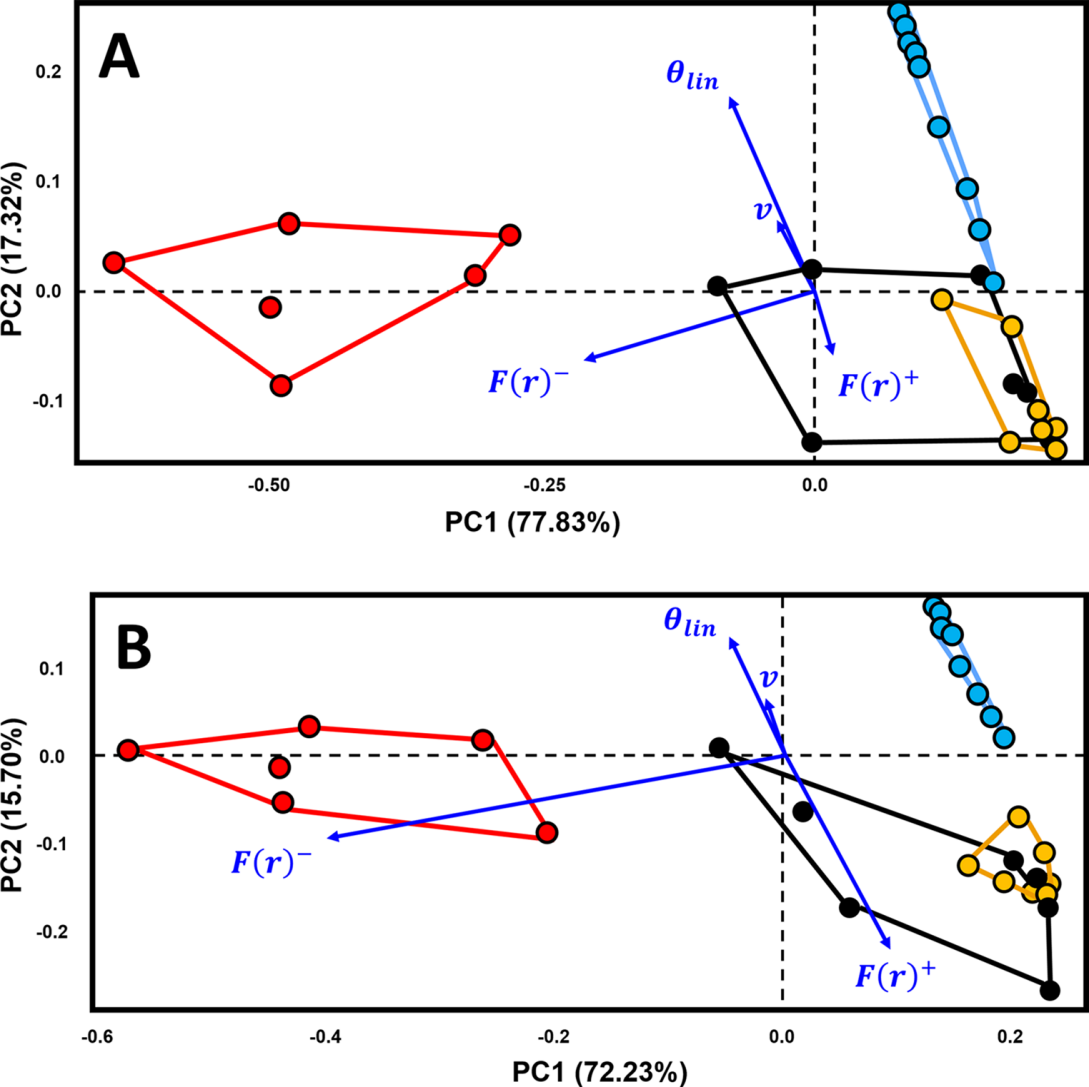
Principal Components Analysis bi-plots computed (**A**) excluding Palaeolithic samples of potential AMSs (black) from the calculation of PCA, and projecting them onto the constructed PC scores, and (**B**) excluding ethnographic SMOs and AMSs (orange) from the calculation of PCA, and projecting these samples later onto the constructed PC scores

The only exceptions to the observed patterns are found when considering Mardia-Watson-Wheeler test results for angles, which reveal notable differences between marked artefacts (Table S3; *W*_*g*_ >34.3, *p* < 3.7 × 10^− 8^, FPR < 0.001%). The extreme concentration of θ values along the perpendicular axis of the bone (Fig. S81) lead known examples of SMOs and AMSs to present a smaller amount of variation and thus appear different to their Palaeolithic counterparts (Table S3). Nevertheless, regardless of these differences in orientation, if we re-evaluate the relationship between samples by removing either the potential Palaeolithic AMSs or ethnographic AMSs from the original calculations, and later project these samples onto the constructed feature spaces (Fig. 7), it is observed that, in all cases, the two samples have a tendency to occupy the same region of each PC score.

## Discussion

The present analysis has shown that the spatial distribution of different types of markings on bone are separable, with distinct patterns emerging for butchery activities, figurative or abstract representations, and potential AMSs; whether the latter be ethnographic examples, or Palaeolithic instances interpreted as such from previous studies. These studies have proposed that four distinct factors, in isolation or combination, may play a role in creating codes allowing for the storage of information in an AMS; the number of marks, the accumulation of marks over time, their spatial organisation and arrangement, as well as their morphology. To date, the identification of potential Palaeolithic AMSs has been based mainly on the technological analysis of marks, the identification of tool changes, and thus the possible accumulation of marks over time (d’Errico 1991, 1995, 1998; d’Errico and Cacho 1994; d’Errico et al. 2017). The spatial organisation, orientation, and morphology of the marks have normally been taken into account to discuss the type of code used by the potential Palaeolithic AMSs in terms of the quantity and variety of information stored, but not as a formal criterion for identifying potential AMSs in the archaeological record (d’Errico 1995, 1998). Likewise, these patterns have mostly been based on *in-visu* descriptions. The method applied here, on the contrary, efficiently quantifies these features, and thus presents a means of identifying the compartmentalisation and organisation of markings in space. While this method is independent of the technological analysis of the marks, the combination of these sources of information in future analyses should be beneficial for the identification and interpretation of early instances of SMOs and AMSs.

In the SMO / AMS sample analysed here, efficient storage of information is achieved by developing marking techniques adapted to the size and morphology of the object, the nature and amount of information to be stored, and the specific function of the SMO. Efficient retrieval is made possible by readily perceptually discriminating patterns, such as those described by the Gestalt Principles (Pelli et al. 2009), in particular; proximity, similarity, symmetry and continuity. The variability observed within modern and Palaeolithic SMOs and AMSs, exemplified by multivariate analyses, probably reflects greater or lesser compliance to these perceptual rules. It certainly also depends on variations in social learning processes, including efficiency in the transmission of codes and attached meanings, degree of conventionalization, size of the practitioners’ community, as well as successful embodiment of the cognitive functioning of each SMO/AMS type (Morin et al. 2020; Gweon 2021).

Although the sample to which the method has been applied is still relatively small, the results are robust, supported by simulation based approaches, and open up new perspectives for the recognition and characterisation of the earliest potential AMSs. The applied methodology was able to independently identify clear commonalities between, on the one hand, ethnographic AMSs used for different purposes and developed in different cultural and economic contexts and, on the other hand, Palaeolithic objects interpreted in the past as AMSs. Additionally, the inclusion of Palaeolithic figurative representations and butchery marks adheres to the falsifiability criterion, allowing for a comparison of our data with instances clearly identified as non-AMS objects, thus consolidating that these artefacts bear distinct features.

According to our findings, the consistent distribution and standardized orientation of markings play a crucial role in the design of these proposed AMSs. These devices typically display regularly spaced marks or incisions, perpendicular to the primary axis of the canvas where information is being stored. Compartmentalisation is also often employed to separate distinct groups of markings. This can be statistically detected through an inhibition of two sets of markings occurring in the same space, whether these distinct sets are produced by different tools and techniques (e.g. La Marche), to separate entities being recorded (e.g. the Medieval English tally sticks), or by their association with pictographic symbols (e.g. the 1st -century Mirān tally stick). The examined ethnographic datasets included examples of notched sticks, some of which exhibit quantity-related representations, and some that strongly suggest some form of expressive quantification. They are documented from 20th -century artefacts from Muacapenda and Muatchondo (Angola), tally sticks from Medieval England, as well as 19th -century notched wooden artefacts from the Yakunbura community of the Dawson river (Australia). This raises a plausible interpretation for these artefacts, related with the emergence of quantitative cognition in our species. Although these artefacts present no additional discernible information about their function based solely on the markings beyond the data that has been collected ethnographically or historically, our statistical analyses show that they share many properties with the potential Palaeolithic AMSs in our sample. An important remaining question, however, is to determine what aspects of expressive quantification might be involved in Palaeolithic AMSs, since quantification is a multifaceted phenomenon that encompasses a wide range of features and cognitive mechanisms.

### On AMSs and the possible emergence of quantification

Quantification is a complex topic that involves a number of cognitive functions in order to reach certain degrees of precision. These cognitive mechanisms may vary in terms of formal complexity, levels of abstraction, and socio-cultural developments; going from mere perceptual discrimination to linguistically supported number concepts, counting procedures, and eventually arithmetic and elaborated mathematical concepts as well (Núñez 2017).

Expressing quantification goes far beyond the perceptual discrimination of items observed in non-human animals and human infants at an early age. Numerous species, spanning non-human and human domains, possess neurally supported psychophysical capacities for perceptually discriminating quantities of items under specific conditions (Beran et al. 2008; Cantlon 2012; Rugani et al. 2015; Benson-Amram et al. 2017). These capacities are biologically evolved and operate in a non-symbolic manner. One such capacity allows individuals of various species to instantaneously, effortlessly, and without mistakes, perceptually discriminate small collections of objects containing up to about three items—a phenomenon referred to as *subitizing* (Mandler et al., 1982; Feigenson et al. 2004). Another biologically evolved psychophysical capacity shared by many species is that of being able to perceptually discriminate between groups of items when they stand in a distinct enough ratio (Xu and Spelke 2000), known as Large Quantity Discrimination (LQD). This is hypothesized to be neurally instantiated via the so-called *Approximate Number System* (ANS) (Piazza 2010). Termed “quantical capacities” (Núñez 2017), these biologically evolved capacities, with adaptive value, are observed in both extant primate and non-primate species, strongly suggesting their presence in all extinct hominin species. Crucially, however, these quantical capacities do not involve *symbolic reference*, and since they operate under biologically imposed strong working memory constraints, manifest themselves with a reduced scope in amount-handling (subitizing) and exactness (LQDs). Importantly, the acquisition of specific instances of symbolic reference (e.g., referring to the quantity in a pair of items with the word “two”) must occur through learning (in ontogeny) as it cannot be biologically evolvable, lacking the necessary natural associations and corresponding trans-generational reproductive consequences (Deacon 2011). This pivotal characteristic opens the door for cultural evolution, distinct from purely biological evolution.

Nevertheless, these fundamental biologically evolved quantical capacities serve as essential building blocks for the development of symbolic forms, enabling the off-loading of cognition and overcoming the limitations of working memory. Symbolic forms, coupled with other fundamental and generic cognitive functions such as 1–1 correspondence mappings or the production of ordered lists (i.e., the enumeration of items, which may stimulate tally production), operate on these capacities when supported by appropriate cultural preoccupations, motivations, and inventions. They therefore have the potential of eventually leading to much more complex forms such as those that have given rise to the concept of number, counting, arithmetic and mathematics (Núñez 2017). If expressive quantification function was attached to these potential Palaeolithic AMSs, it must have used the fundamental trait of symbolic reference. Marks would not be purely perceptually processed; instead, they would signify some quantity-related content. Material expressive quantification can manifest at various levels, ranging from marks that orderly list events or people without specific numerical reference,as seen in many Aboriginal Australian message sticks (Kelly 2020, 2024), to marks that visually express discernable contrasts of approximate relative quantities (discriminable via LQD mechanisms), to marks that express actual counting procedures implemented via linguistic resources that include a specific numeral lexicon. What is important, however, is that most of these levels of expressive quantification require, at least in intention, the establishment of 1-to-1 correspondence mappings between physical marks and external items, words, or ideas, a characteristic that the Palaeolithic SMOs and AMSs studied here appear to exhibit.

It is important to point out that in the case of Australian message sticks, these artifacts function primarily as tools of social cognition (see Overman, 2023; Kelly 2024). It would therefore be important to highlight that because the present study only considered a subsample of message sticks, more developed analyses in the future are necessary to potentially understand the minute variations that might exist in potential subsets, helping differentiate what might be considered an SMO from an AMS more clearly.

A number of artefacts recovered from the Palaeolithic such as those from Les Pradelles (72–60 ka, France), Border Cave (44–42 ka, South Africa), and Solutré (22 − 17 ka), exhibit a lack of discernible variations in the markings morphology that could be used to attribute different meanings to different marks. Given their homologous nature, whether representing lists of people or other natural items, or symbolizing magnitudes partitioned in discrete units (e.g., days), if these artefacts were to be considered an AMS, their markings would appear to have conveyed their meaning symbolically primarily through abstract quantity-related marks, rather than through distinct variations in their appearance. In other words, these devices could have only been used to record and retrieve numerical information related to items or events of the same nature. Other Palaeolithic AMSs such as the Blanchard ivory spatulas (39–34 ka, France), the Laugerie-Basse rib (14–12 ka, France), the Tossal de la Roca pendant (11 ka, Spain), and the La Marche antler (15 ka, France), exhibit groups of marks deliberately produced with different techniques and tools in order to facilitate their visual discrimination. Along these lines, however, it is important to clarify the definition of 1-to-1 correspondence. While this is a necessary cognitive resource for counting, it is not exclusive to numerical tasks. For instance, 1-to-1 mapping can support non-numerical listings where each mark corresponds to an item or event without assigning it a number, as in the naming of family members. In such a context, the proposed Palaeolithic AMSs may have functioned as storage devices for sequences of distinct elements, even if they did not encode exact quantities.

It is important to point out, therefore, that inscribing marks on an object, even deliberately and across multiple sessions may constitute instances of AMSs with various symbolic functions, but it doesn’t necessarily prove that the manufacturer intended to store *quantity-related* information. Examples of this can be found in the case of multiple message sticks recovered from Australia (see Overman, 2023; Kelly 2024). Additionally, it doesn’t indicate whether the manufacturer possessed the cognitive and linguistic tools to engage in expressive quantification, or to “count” the marks precisely; either during their creation or afterwards. Linguistic anthropological evidence from a multitude of global languages, particularly concentrated in the Amazon basin and in Australia, reveal that many of these languages have a strikingly limited lexicon (spoken numerals) for exact quantification, typically restricted to quantities of one to about three to four items —i.e., within the subitizing range (Epps et al. 2012; Bowern and Zentz 2012; Zariquiey et al. Accepted). These languages have been referred to as anumeric languages, spoken by individuals from anumeric cultures (Everett and Madora 2012). In these socio-cultural linguistic environments, counting procedures — i.e., involving the assignment of a specific numeral to each item in a collection beyond subitizing range — are not possible. These relevant data from contemporary human societies challenge the notion that *counting* is an innate trait (contra some popular claims; Butterworth 2022) and highlights its complex cultural nature. Nevertheless, these anumeric languages, although being rich, subtle and sophisticated in many other dimensions, do possess natural quantifiers equivalent to the English terms “few”, “several”, or “many”, as well as other grammatical resources to handle their function. While they are not numerically precise, they are nonetheless semantically precise (i.e., their *meanings* can be distinguished unambiguously), and are highly functional and efficient in handling a wide range of activities in everyday life. The largely universal presence of specific spoken numerals for exact small quantities (e.g. “one”, “two”, “three”) in languages globally, including Amazonia and Aboriginal Australia, aligns with the frequent perceptual experience provided by subitizing. Similarly, the universal presence of spoken natural quantifiers, that distinguish between “few”, “several” and “many”, is consistent with the frequent and ubiquitous perceptual experience that Large Quantity Discrimination (LQD) provides, facilitating contrastive quantity-related comparative functions, such as “more”, “less”, “greater”, “smaller”, and so on.

While it remains unknown what linguistic resources the Palaeolithic SMO / AMS manufacturers had at their disposal, or whether they were members of anumeric or numeric cultures, it is certain that they had subitizing and LQDs in their perceptual-cognitive repertoire (as these are manifested in many extant non-human species today). If these manufacturers had developed some kind of cultural preoccupations that prompted them to overcome working memory constraints associated with quantification, it is conceivable that they employed symbolic reference to support further resources, like 1–1 correspondence, for materially expressing quantification involving collections beyond the subitizing range. This is suggested by the systematic and homogeneous marks present in several Palaeolithic AMSs analysed here, all displaying markings in quantities larger than the subitizing range. It is therefore plausible that these marks were produced and monitored through careful visuo-haptic actions closely coordinated with underlying cognitive mechanisms, including those involved in working memory and attentional control (Coolidge and Wynn 2005; Cooliedge and Overmann, 2012). However, the impact of working memory on such fundamental cognitive behaviours remains underexplored in empirical terms and has yet to be experimentally tested in relation to archaeological artefacts and how they may have been handled, perceived, or conceptualised.

For the sake of scientific accuracy, we must also consider other constraints for interpreting the possible expressive quantification in Paleolithic SMOs and AMSs. In addition to insights from linguistic anthropology, research by cognitive developmentalists and child psychologists have produced compelling evidence challenging the assumption that concepts like “number,” “counting,” or “numerical equivalence” are innate or easily acquired (Piaget 1968). Numerous findings indicate that these concepts are complex, composite and multi-faceted, requiring years to be acquired and mastered by the human mind, even with extensive cultural scaffolding, intentional teaching, and enculturation. For instance, research in these fields has shown that children can manifest spatial enumeration without involving counting (Potter et al., 1968), be able to deploy 1–1 correspondence without having acquired the conception of exact number or numerical equality (Izard et al. 2014; Schneider et al. 2022), and that learning (and being able to recite) the list of counting numbers does not imply being able to understand the Cardinal Principle (CP), i.e., the capacity to accurately label and construct collections of items via counting (Sarnecka and Carey 2008). Studies have also shown that the knowledge of the list of counting words, even when having acquired the CP, does not imply the understanding of exact numerical equality (Schneider et al. 2022). Moreover, they have shown that the generalization of the successor function (that allows for a genuine comprehension of the numerical structure of counting numbers) is only achieved years after having learned counting procedures (Cheung et al. 2017). Finally, longitudinal studies in cognitive developmental neuroscience have been able to characterize the plasticity and constraints operating on the neural correlates of the enculturation process that underlie the gradual piecemeal quantity-related developments (Ansari 2008). Taken together, and considering that all these findings have been obtained with populations of children from the modern industrialized world with extended exposure to systematic written-based schooling practices, these and other similar results provide an ontogenetic baseline for what would have been possible for, and what challenges would have been faced by, Paleolithic SMO / AMS manufacturers when handling and developing expressive quantification.

A growing body of interdisciplinary literature emphasizes the significance of the devices investigated in our study, and of more ephemeral modalities for storing or just transmitting coded information as cognitive tools. These tools are stimulated by specific cultural preoccupations (Núñez 2017), shaped by social and economic necessities (Schlimm and Neth 2008; Bender et al. 2015; Bender and Beller 2018; Colagè and d’Errico 2020), and supported by cognitive linguistic resources for abstraction, imagination, and invention (Lakoff and Núñez 2000). Consequently, they can hardly be understood as being solely driven by innate “number”-specific human predispositions (Butterworth et al. 2008). Indeed, the practices of quantification — whether employing conventionalized numeral words, natural quantifiers, various grammatical constructions with their prosodic cues, finger counting, or exosomatic tools like AMS or written number symbols — have evolved culturally, and manifest themselves in diverse and complex ways across different cultures (Bender and Beller 2012, 2018; Beller et al. 2018).

In order to produce and retrieve information inscribed on the ethnographic SMOs and AMSs, users of these objects would have had to deploy symbolic reference to materialize external representations. In the case of many Aboriginal Australian message sticks (Kelly 2020, 2024), marks can unambiguously denote quantities within the subitizing range, signifying terms like “pair” (a term that is not a counting numeral). However, given the widespread and well-documented absence of spoken (or written) numerals beyond that subitizable range in traditional Aboriginal Australia, counting, in the proper sense, is not directly achievable. Nevertheless, larger amounts of marks, although not symbolically numerical, could still be produced for other purposes requiring, for instance, 1–1 correspondence such as ordered listings (enumarations) that evoke events, places to be reached during a trip, or names of people. In contrast, in the case of ethnographic AMSs occurring in cultures with spoken numerals for actual counting and writing technology (e.g., Mirān tally stick or the Medieval “English” tally stick), quantity-related marks can be considered “numerical”. Manufacturers in these cultures likely had in their repertoires not only 1–1 correspondence mappings, but also a well-structured numeral lexicon and the mastery of the cardinal principle (CP), so elusive in young children, which makes functional counting efficient and reliable. Since language does not fossilize we have no factual evidence to confirm or disconfirm that Palaeolithic users possessed number words to count the marks when recording and retrieving information on their AMSs. But it is perfectly possible, in the light of our results and above discussion, that these users may have denoted quantity-related information by deploying cognitive resources such as 1–1 mappings, listings (enumerations) of ordered events, or LQD to iconically convey information about the relative size of collections of items.

At this stage we cannot truly speculate about the exact nature of these population’s precise linguistic repertoire. For this reason, we can neither confirm nor deny the possibility that these individuals may have possessed number words allowing them to count the marks that have been deliberately accumulated over time on these objects. Nevertheless, the recording of information by marking objects is a form of external representation, defining a communication technology that implies the transmitting of knowledge within a community, necessitating a shared understanding of the processes behind the production and use of these devices. Given that the specifics of symbolic reference are not biologically evolvable (Deacon 2011), it is difficult to conceive how such technological and cognitive advancement could have not relied on the previous developments of corresponding practices, motivating cultural preoccupations, and linguistic resources and would have not prompted their further development by a process of inevitable coevolution. Regarding expressive quantification, in the work of d’Errico et al. (2017) and Schlaudt (2020) the authors discuss how the emergence of AMSs during the Upper Pleistocene could support a five-stage scenario that has the potential to elucidate how prehistoric cultures transitioned from raw quantical cognition (e.g., subitizing and large quantity discrimination), that humans share with numerous other species, to develop more elaborated quantity-related notions. This scenario would involve successive layers of enculturation and cultural exaptation’s, defined as the repurposing of existing cultural elements for novel functions.

## Electronic supplementary material

Below is the link to the electronic supplementary material.

Supplementary Material 1

Supplementary Material 2

## Data Availability

Data can be provided upon reasonable request to the corresponding author or Francesco D'Errico. Detailed documentation of all artefacts studied, however, have been included in supplementary materials, alongside sample R code.

## Declarations

### Materials and correspondence

All correspondence should be directed to the corresponding author, Lloyd A. Courtenay (ladc1995@gmail.com).
