# Supplementary material for: Identifying potential palaeolithic artificial memory systems via Spatial statistics: Implications for the origin of quantification
Source: Archaeol Anthropol Sci. 2025 Jul 23;17(8):171. doi: 10.1007/s12520-025-02286-4 (PMC12287167; doi:10.1007/s12520-025-02286-4)
Supplement: Supplementary file 2 — Supplementary Material 2 [file 12520_2025_2286_MOESM2_ESM.pdf]

SUPPLEMENTARY APPENDICES FOR "IDENTIFYING POTENTIAL PALAEOLITHIC ARTIFICIAL MEMORY SYSTEMS VIA SPATIAL STATISTICS. IMPLICATIONS FOR THE ORIGIN OF QUANTIFICATION AND WRITING"

QUANTA PROJECT

Lloyd A. Courtenay
Préhistoire à l'Actuel Culture, Environment et Anthropologie (PACEA UMR 5199)
University of Bordeaux & CNRS, France
ladc1995@gmail.com

Francesco D'Errico
Préhistoire à l'Actuel Culture, Environment et Anthropologie (PACEA UMR 5199)
University of Bordeaux & CNRS, France
& Centre for Early Sapiens Behaviour (SapienCE)
University of Bergen, Norway
francesco.derrico@u-bordeaux.fr

Rafael Núñez
Eigdenössische Technische Hochschule (ETH), Zürich
rafael.nunez@gess.ethz.ch

Damián Blasi
Catalan Institute for Research and Advanced Studies (ICREA), Spain
& Center for Brain and Cognition
Pompeu Fabra University, Spain
dblasi@fas.harvard.edu

June 24, 2025

ABSTRACT

The following document contains all of the supplementary information related to our paper "Identifying Potential Palaeolithic Artificial Memory Systems via Spatial Statistics. Implications for the Origin of Quantification and Writing". These include the updated drawings of each artefact, all $F(r)$, $g(r)$, $K_{ij}(r)$, and rose diagrams for each artefact, supplementary figures and tables that form part of the results, and a section justifying the validity of our clustering results.

Keywords Spatial Statistics · Point Pattern Processes · Artificial Memory Systems

Contents

1 Appendix A: Technical Drawings of the artefacts studied **3**
1.1 Butchery Samples . . . 3
1.2 Decorative / Depiction / Artistic Samples . . . 6
1.3 Potential Pleistocene AMS Samples . . . 11
1.4 Holocene C.E. Notation System Samples . . . 16

2 Appendix B: Supplementary Figures ($F(r)$, $g(r)$, $K_{ij}(r)$, Rose Diagrams) **20**
2.1 Butchery Results . . . 20
2.2 Decorative / Depiction / Artistic Results . . . 22
2.3 Potential Pleistocene AMS Results . . . 25
2.4 Holocene C.E. Notation System Results . . . 33
2.5 Additional Results . . . 37

3 Appendix C: Supplementary Figures (PCAs and Cluster Plots) **37**

4 Appendix D: Supplementary Tables **40**

5 Appendix E: Validation of Clustering Results **42**

References **49**

1 Appendix A: Technical Drawings of the artefacts studied

1.1 Butchery Samples

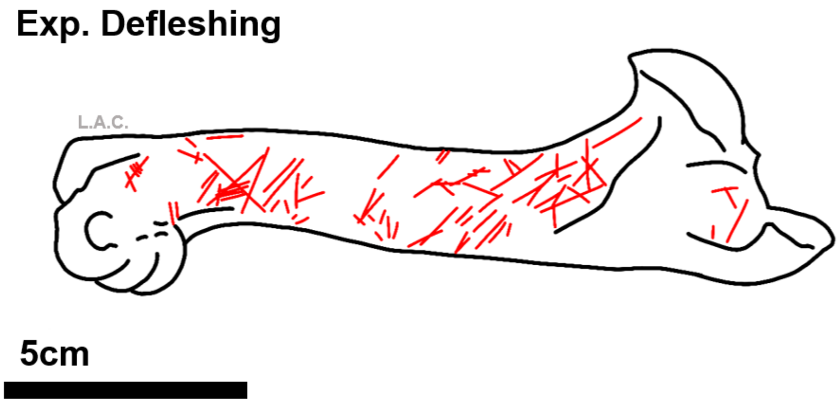

Supplementary Figure 1: *Illustrated drawing adapted from Costamagno et al. (2018) displaying the distribution of marks produced during experimental butchery defleshing activities. Scale bar in the original drawing by Costamagno et al. (2018) was not supplied, therefore an approximate reference of scale taking the average size of deer humeri.*

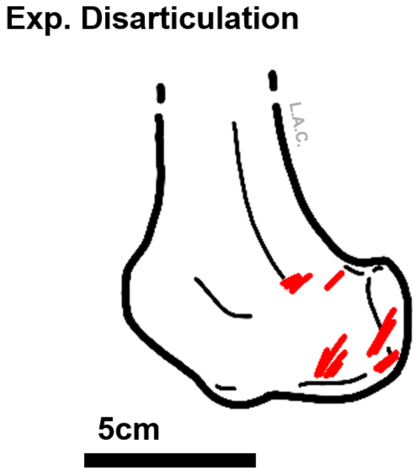

Supplementary Figure 2: *Illustrated drawing adapted from Costamagno et al. (2018) displaying the distribution of marks produced during experimental butchery disarticulation activities. Scale bar in the original drawing by Costamagno et al. (2018) was not supplied, therefore an approximate reference of scale taking the average size of deer femora.*

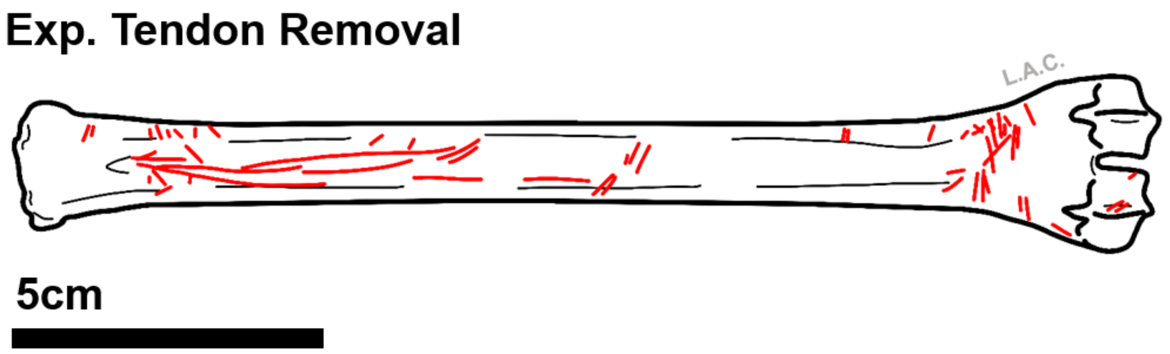

Supplementary Figure 3: *Illustrated drawing adapted from Costamagno et al. (2018) displaying the distribution of marks produced during experimental butchery tendon removal activities. Scale bar in the original drawing by Costamagno et al. (2018) was not supplied, therefore an approximate reference of scale taking the average size of deer metapodials.*

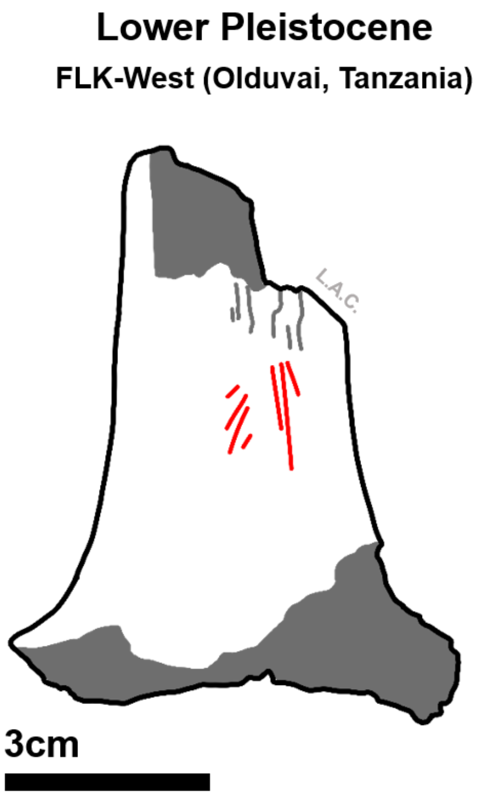

Supplementary Figure 4: *Illustrated drawing adapted from Courtenay (2019), displaying the marks found on the distal epiphysis of a size 3 bovid femur from the 1.7 Ma site of Frida Leakey Korongo West*

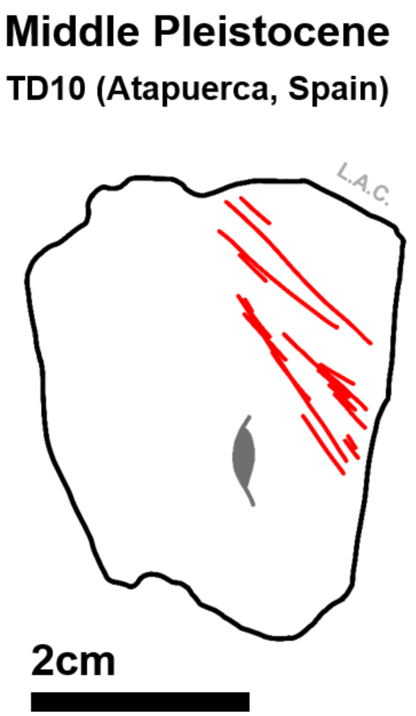

Supplementary Figure 5: *Illustrated drawing adapted from a photograph published in Figure 10 of Rodríguez-Hidalgo et al. (2015), depicting cut marks on a femoral fragment from Gran Dolina (Atapuerca, Spain)*

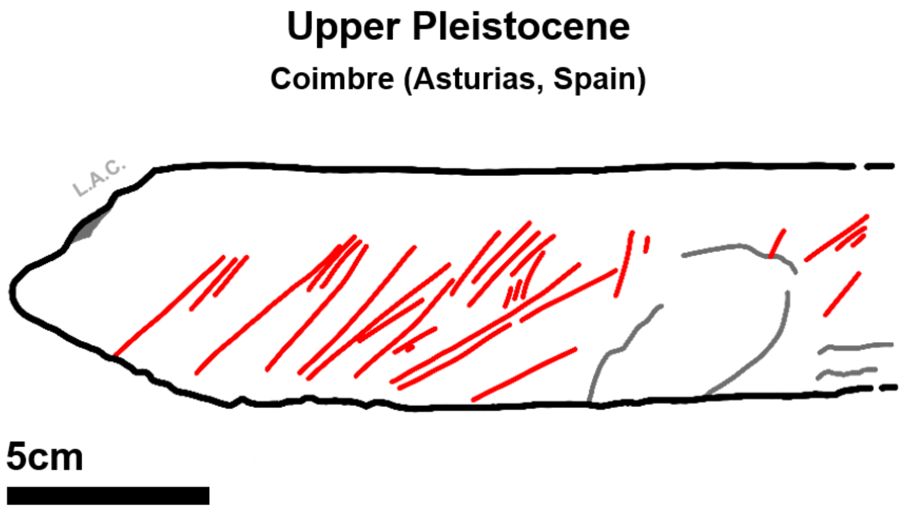

Supplementary Figure 6: *Illustrated drawing adapted from a photograph published in a figure found on page 571 of López Cisneros (2020), depicting a diaphyseal fragment recovered from the Upper Pleistocene site of Coimbre (Asturias, Spain)*

1.2 Decorative / Depiction / Artistic Samples

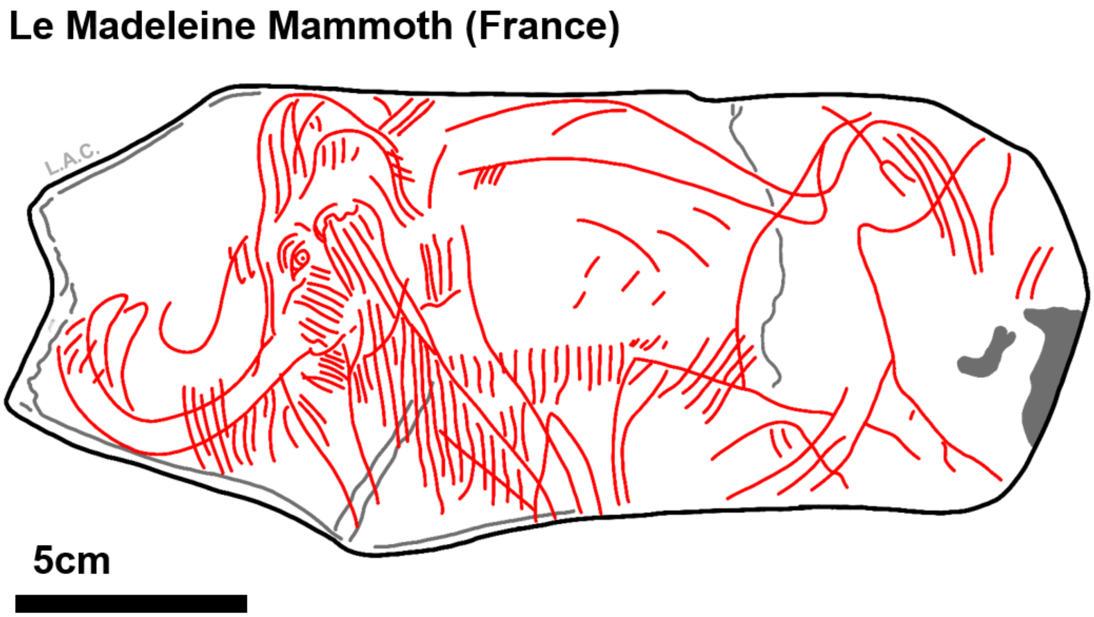

Supplementary Figure 7: *Illustrated drawing adapted from Lyell (1873) of the fragment of ivory from Abri de la Madeleine bearing an engraving of a Mammuthus primigenius individual*

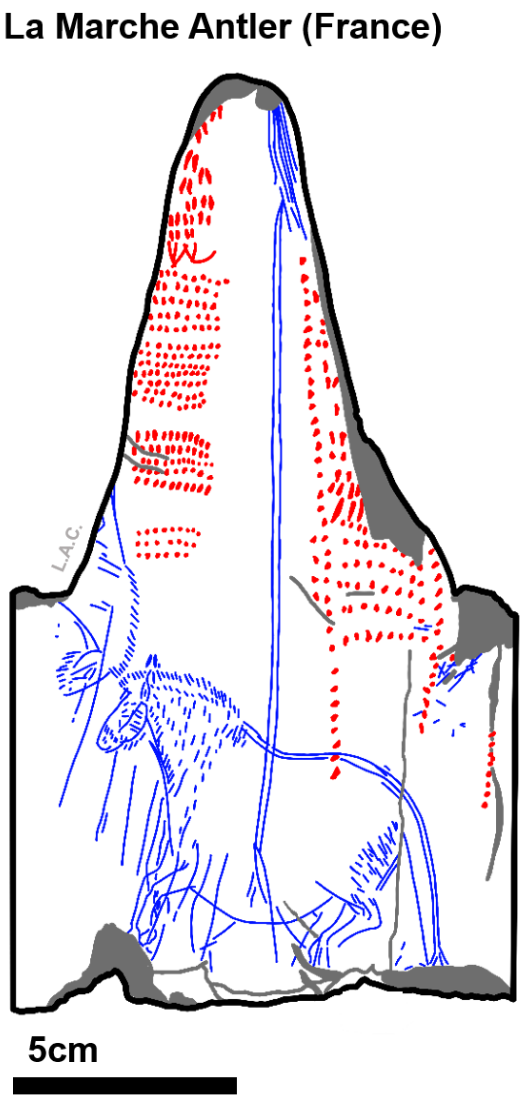

Supplementary Figure 8: *Illustrated drawing adapted from D'Errico (1995) of the fragment of antler recovered from La Marche, bearing an engraving of two horses towards the base of the antler. The decorative markings found on this artefact are marked in blue.*

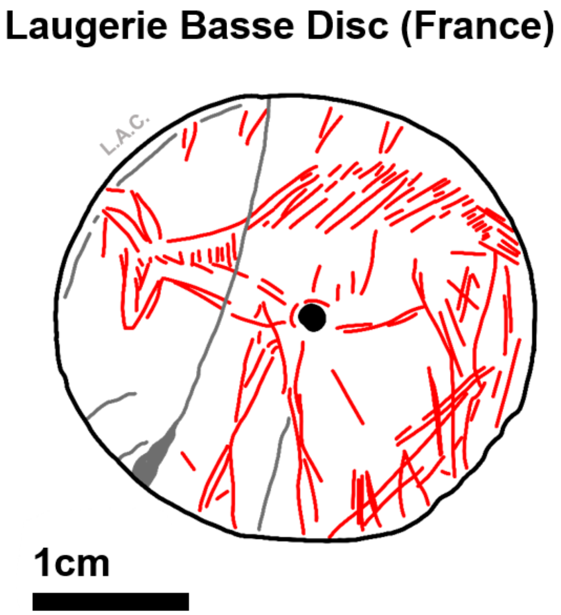

Supplementary Figure 9: *Illustrated drawing of one of the faces of an artefact recovered from the French site of Laugerie Basse (Hardy, 1868)*

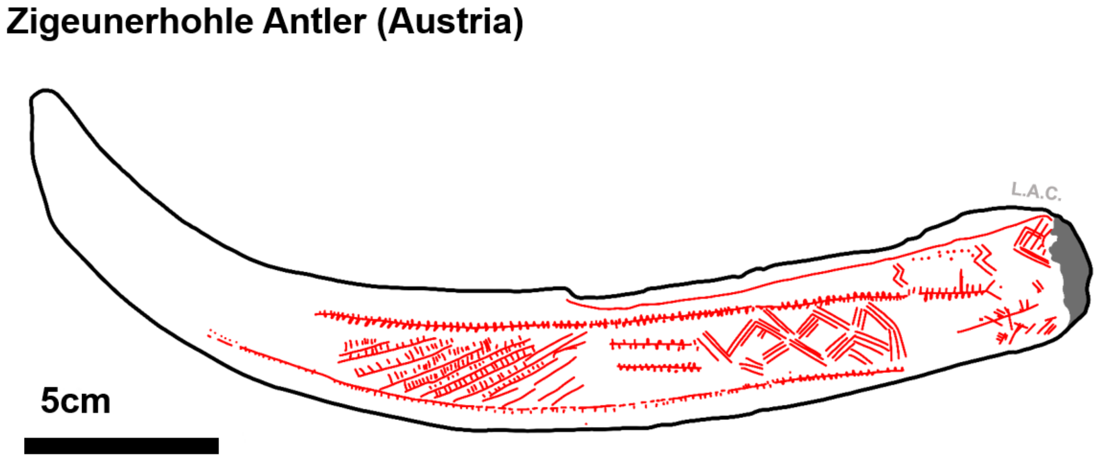

Supplementary Figure 10: *Illustrated drawing adapted from Marshack (1987) of the point of a deer antler from Zigeunerhohle (Austria), bearing a a series of geometric forms engraved along the surface.*

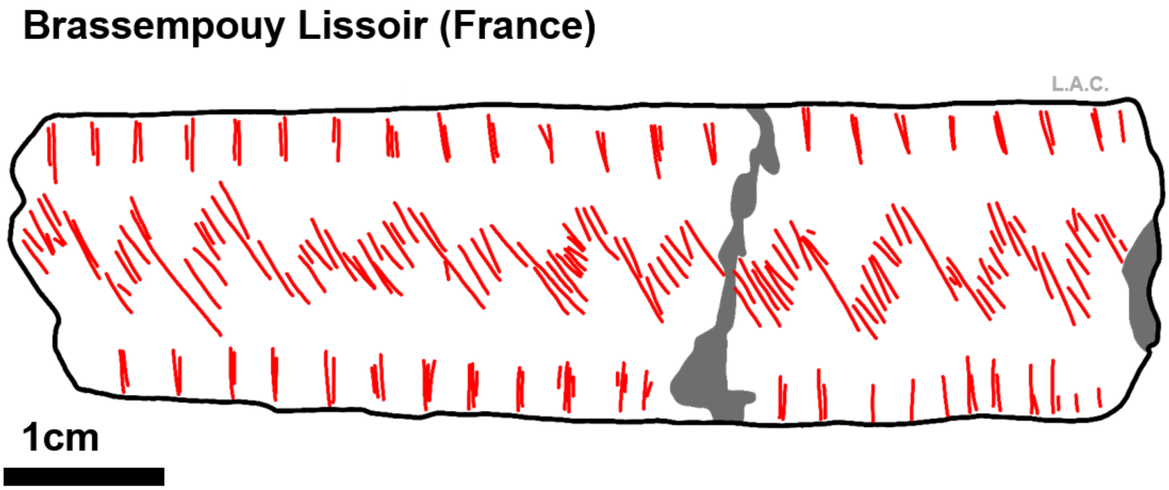

Supplementary Figure 11: *Illustrated drawing adapted from Bello et al. (2017) displaying geometric art motifs found on a bone (MAN 48716) from the site of Brassempouy, France.*

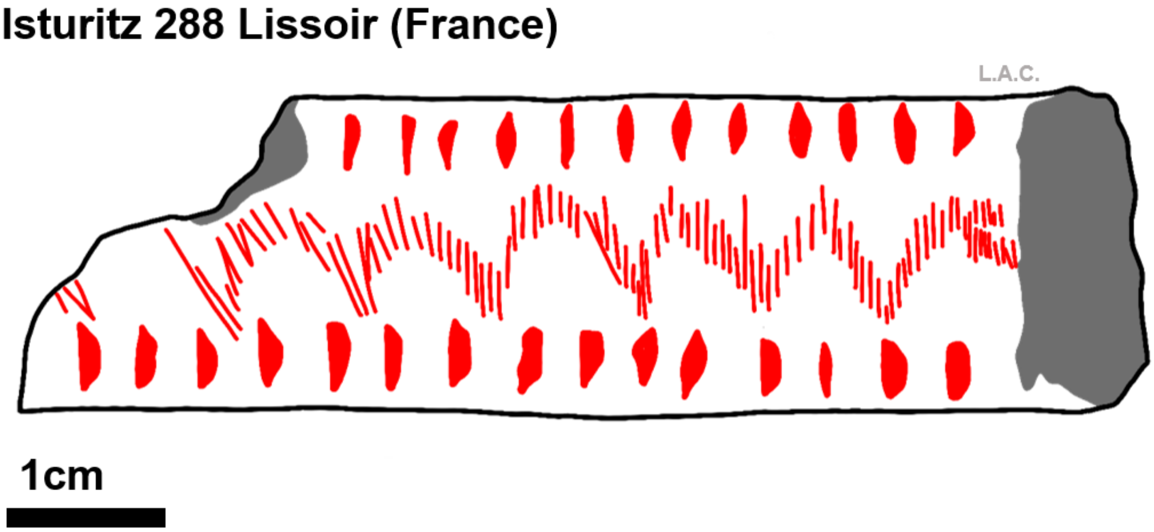

Supplementary Figure 12: *Illustrated drawing adapted from Bello et al. (2017) displaying geometric art motif found on a bone (Ist 288) from the site of Isturitz, France.*

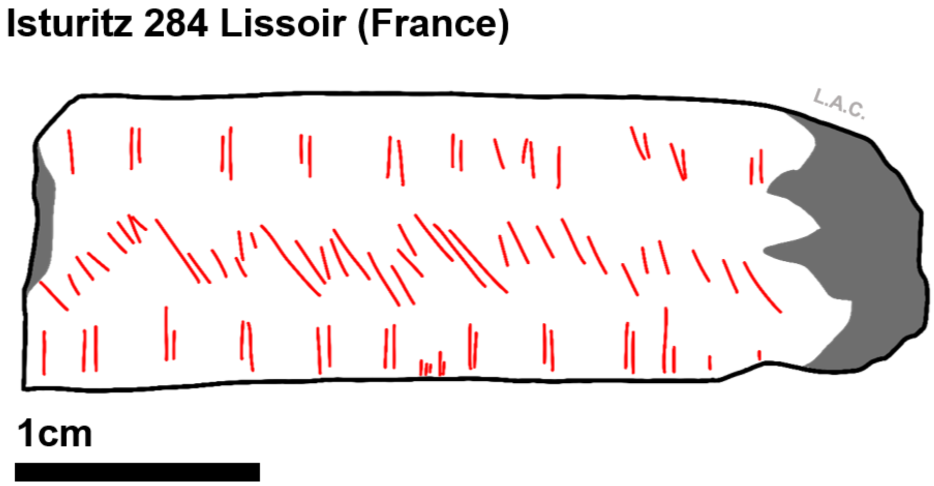

Supplementary Figure 13: *Illustrated drawing adapted from Bello et al. (2017) displaying geometric art motif found on a bone (Ist 284) from the site of Isturitz, France.*

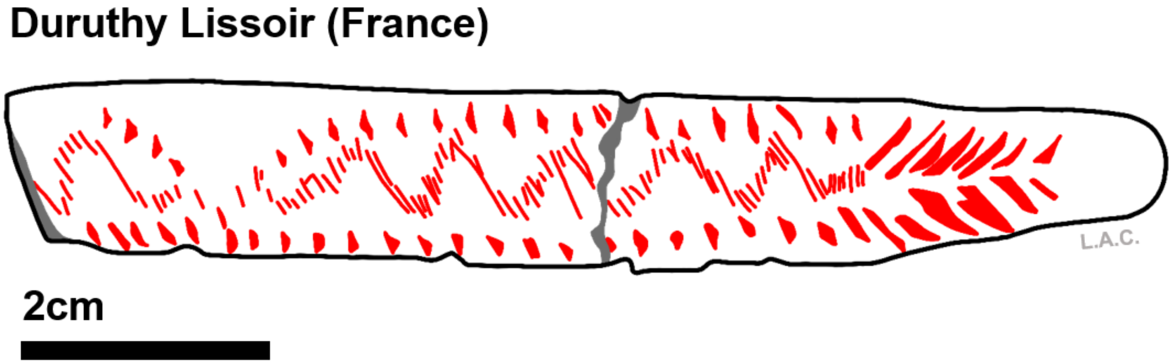

Supplementary Figure 14: *Illustrated drawing adapted from Bello et al. (2017) displaying geometric art motifs found on a bone (Duru 35) from the site of Duruthy, France.*

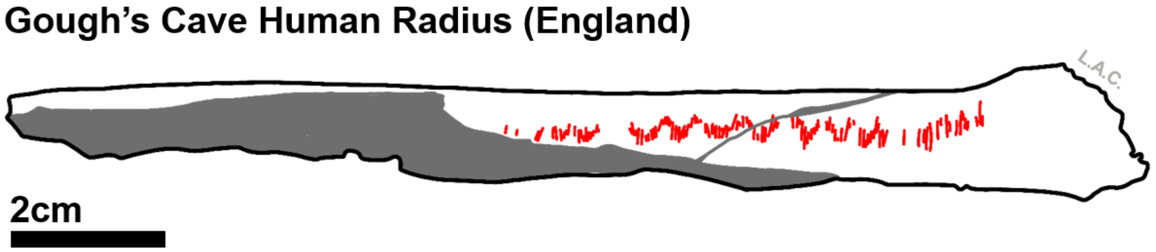

Supplementary Figure 15: *Illustrated drawing adapted from Bello et al. (2017) displaying geometric art motifs found on a cannibalised human bone (M54074) from the site of Gough's Cave, England.*

1.3 Potential Pleistocene AMS Samples

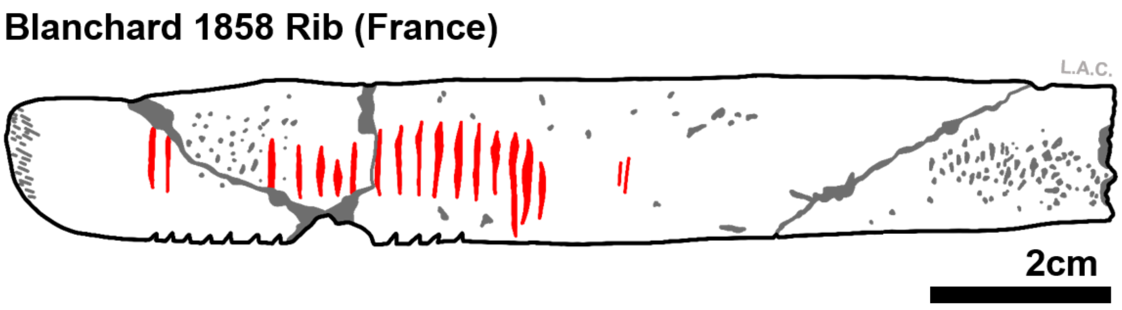

Supplementary Figure 16: *Illustrated drawing adapted from D'Errico (1998) of one of the ribs (D38.23.1858) recovered from Blanchard, France. This rib presents a series of incisions that are too small to have a functional use, such as shafting, and have therefore been classified as being a possible Artificial Memory System (AMS) (D'Errico, 1998)*

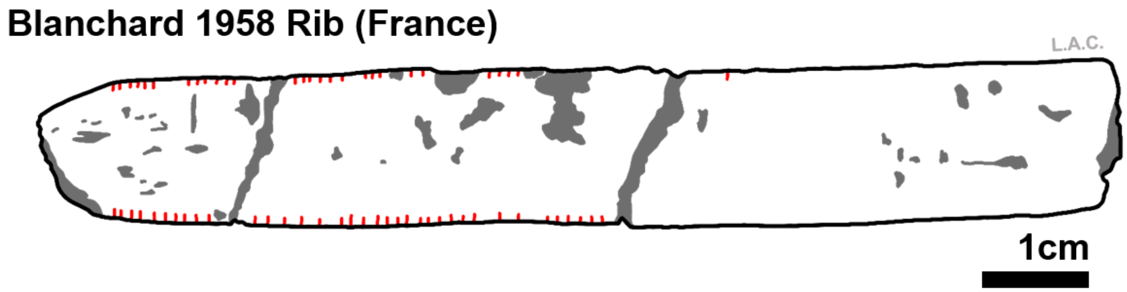

Supplementary Figure 17: *Illustrated drawing adapted from D'Errico (1998) of one of the ribs (D38.23.1958) recovered from Blanchard, France. This rib has been observed to present a series of incisions, that cannot be discarded as having a possible function, maybe linked in some form to knapping activities (D'Errico, 1998)*

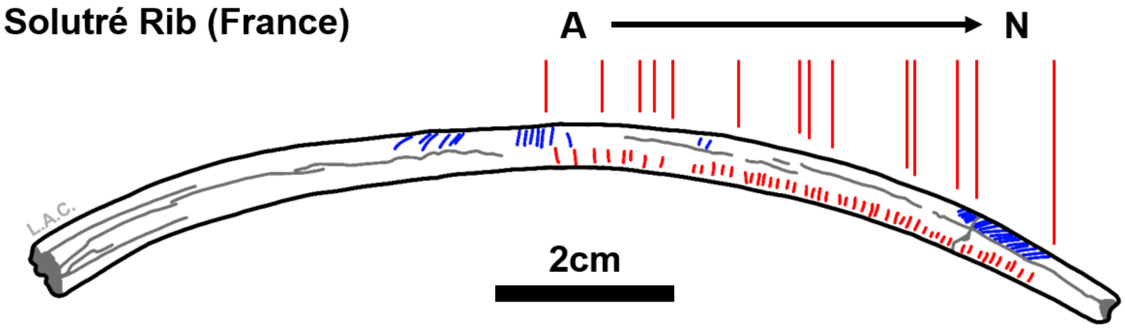

Supplementary Figure 18: *Illustrated drawing adapted from D'Errico (1998) of the Rhino rib recovered from Solutré, presenting in blue, the engravings that have not been identified by D'Errico (1998) of possible Artificial Memory Systems (AMS), and in red the identified engravings considered to be a AMS. Sets of marks within the AMS A through to N, identified by D'Errico (1998), are indicated by vertical lines. A total of 53 notches have been identified on this face of the Solutré rib, produced by a multitude of unretouched cutting edges, most likely over an extended period of time.*

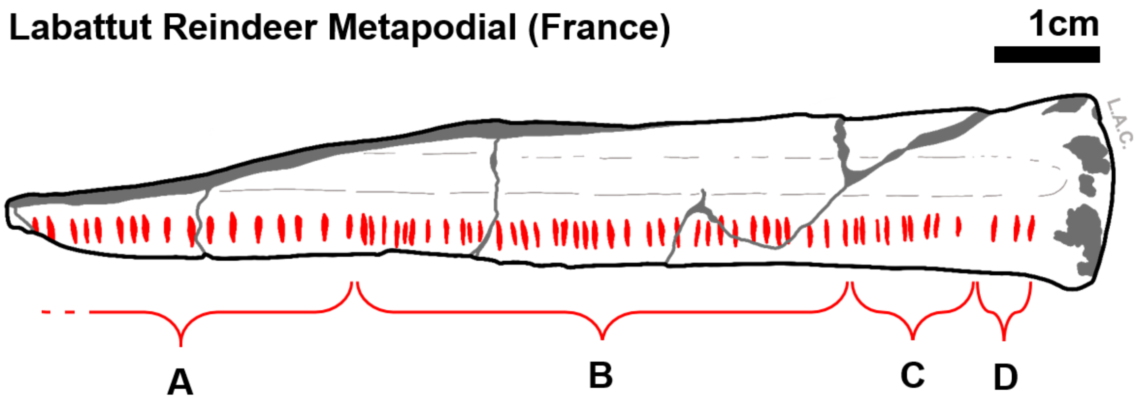

Supplementary Figure 19: *Illustrated drawing adapted from D'Errico (1998) of the reindeer metapodial preserving 4 sets of engravings, interpreted by D'Errico (1998) as an example of a potential Artificial Memory System (AMS), recovered from Labattut. This artefact presents at least 65 notches produced by possibly three or four different unretouched cutting edges D'Errico (1998).*

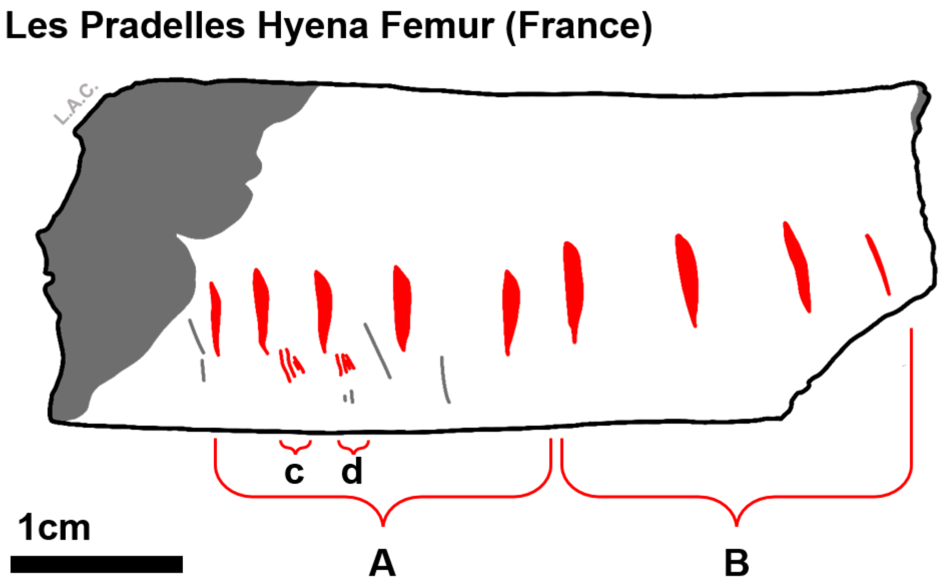

Supplementary Figure 20: *Illustrated drawing adapted from D'Errico et al. (2017) of the hyena femur from Les Pradelles, presenting two main sets (A and B) of engravings considered a potential Artificial Memory System (AMS) by D'Errico et al. (2017), as well as two subsets (c and d). The majority of marks have been interpreted as having been engraved by the same lithic implement, while the division between the two main sets (a and b) has been noted due to a slight change in the orientaiton of the marks. Cross-type interaction analyses between the different sets, however, are not possible as not enough marks are present (D'Errico et al., 2017).*

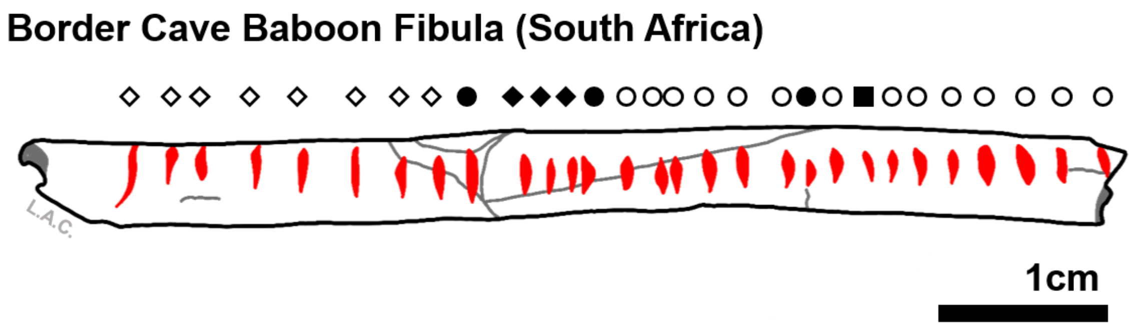

Supplementary Figure 21: *Illustrated drawing adapted from D'Errico et al. (2017) of the baboon fibula from Border Cave. This artefact presents a total of 29 notches along the interosseous crest. D'Errico et al. (2017) identified a total of 5 possible tool changes across the series of engravings along this artefact, symbols above each mark indicate the marks attributed to having been produced by the same tool.*

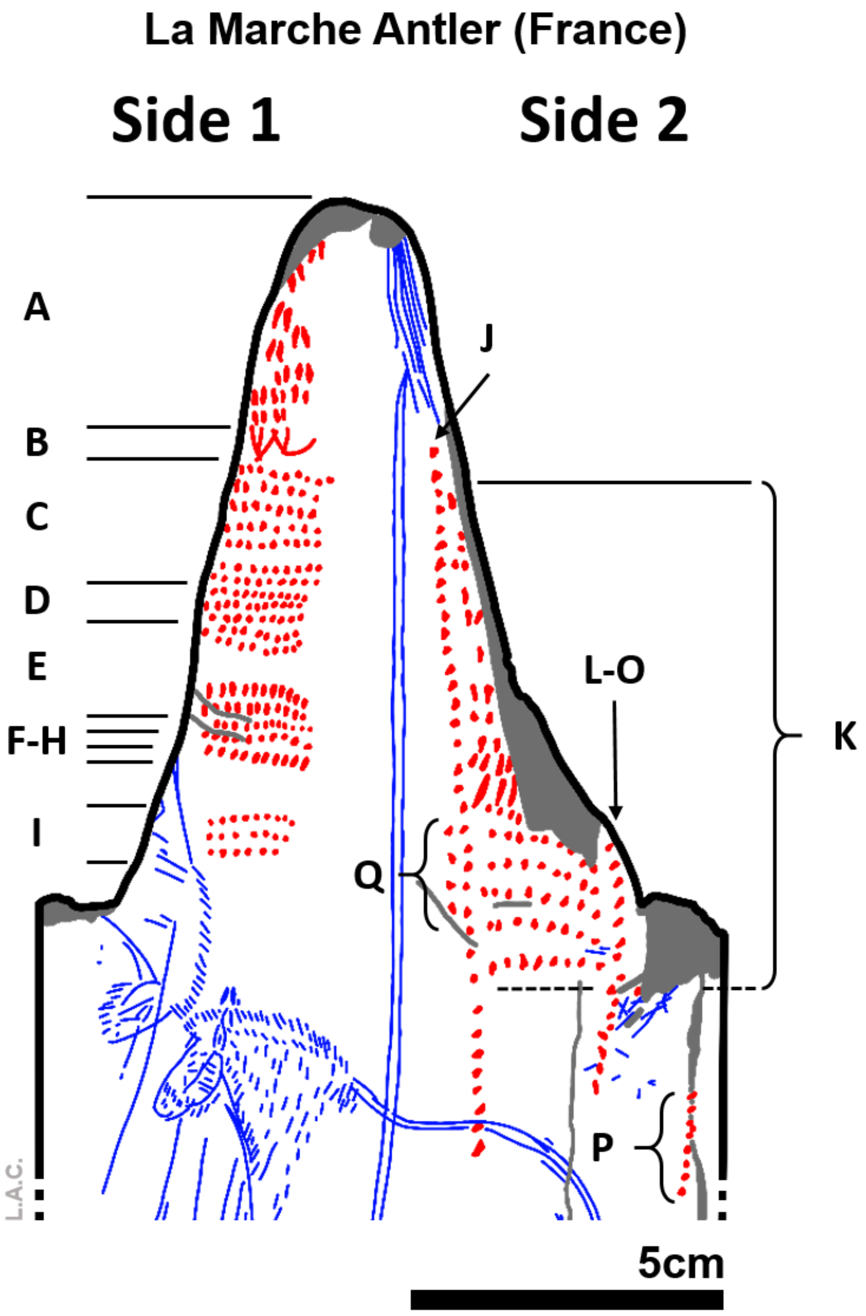

Supplementary Figure 22: *Illustrated drawing adapted from D'Errico (1995) of the fragment of antler recovered from La Marche, bearing a series of markings identified as a potential Artificial Memory System (AMS) by D'Errico (1995). The markings identified as AMSs have been drawn in red, while annotations indicate the division of the marks into main faces, or here-within referred to as sides. In the original publication of this piece by D'Errico (1995), the left hand face of the antler was referred to as Face 2, and the right hand face was referred to as Face 1. In this study we have changed slightly the nomenclature to refer to both "faces" as "sides", while we have switched the numbering round so that set A of markings lies on Side 1 or Face 1, which seems more intuitive. The antler from La Marche displays a complex series of markings interpreted as an AMS by D'Errico (1995), who identified a total of 9 sets with 12 subsets on Side 1, and 8 sets with 14 subsets on Side 2. The indepth analysis by D'Errico (1995) concluded that the engraver aimed to produce the largest number of marks presenting morphological differences while attempting to use the smallest number of tools. In his analysis, the author identified this particular AMS to present a code based on the morphology and spatial distribution of marks.*

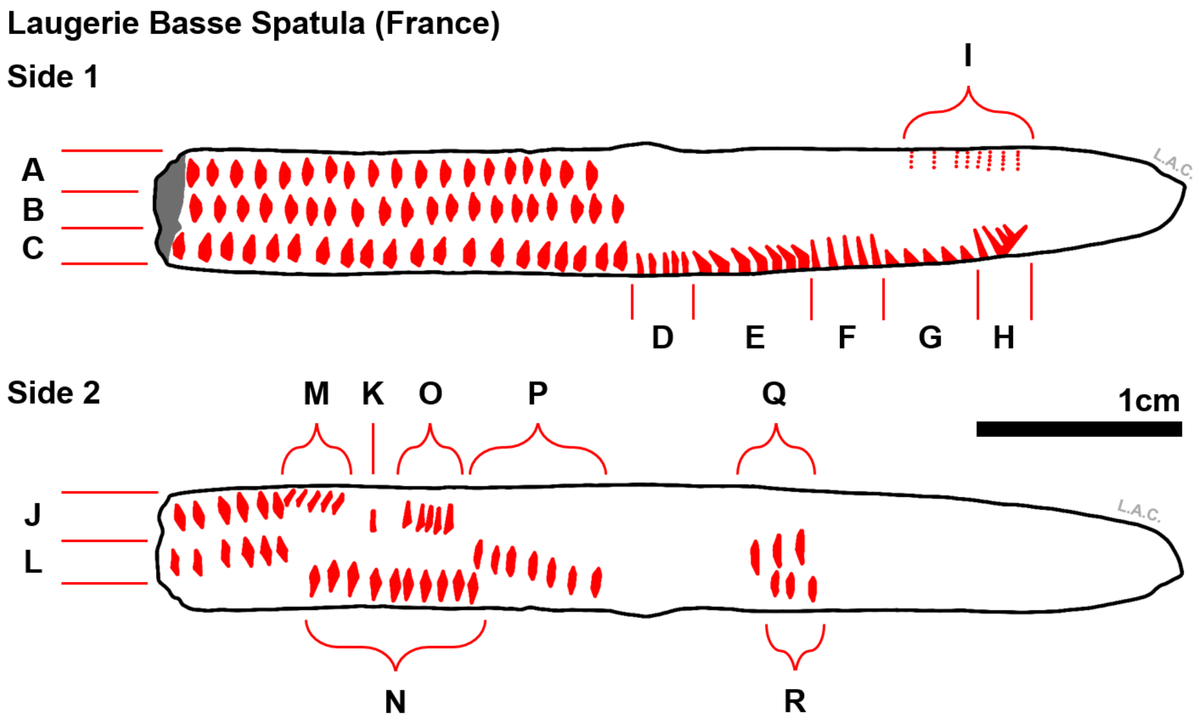

Supplementary Figure 23: *Illustrated drawing adapted from D'Errico (1998) of the spatula from Laugerie Basse. The spatula from Laugerie Basse is an object fabricated from the polishing of a piece of a long bone blade removed from a rib D'Errico (1998). The main face of this spatula presents a large series of marks organised in a total of 9 sets. The opposite side presents a much smaller number of marks divided in 8 sets. One of the sets on side 1 (set I) was additionally erased using scraping. Due to the turning of the piece during the production of marks, some marks present different morphological features although they were probably made by the same tool.*

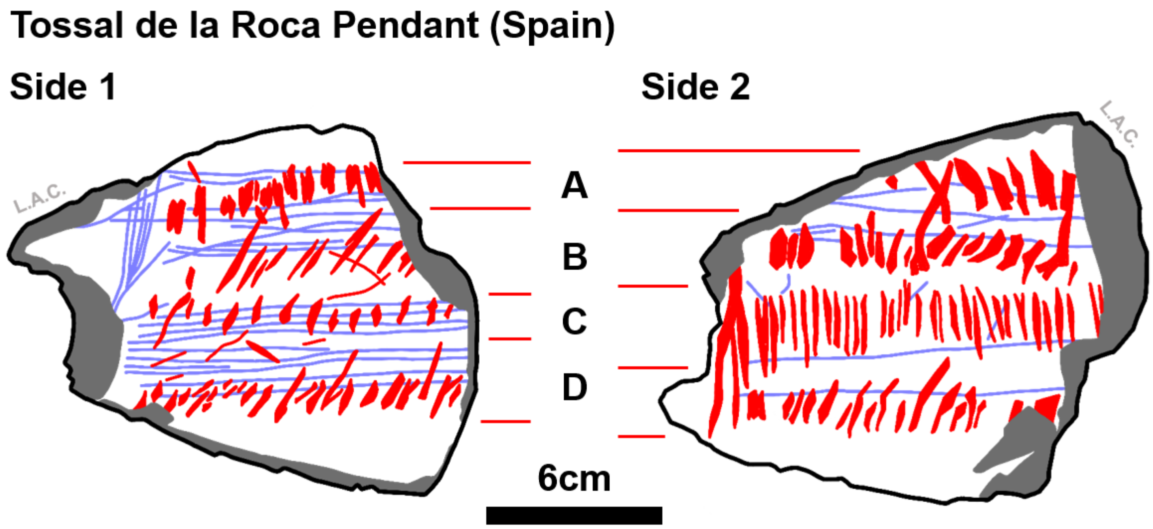

Supplementary Figure 24: *Illustrated drawing adapted from D'Errico and Cacho (1994) of the pendant from Tossal de la Roca. The pendant from Tossal de la Roca is a particularly interesting example of a possible AMS, described in detail by D'Errico and Cacho (1994). This pendant is a small artefact presenting 4 sets of incisions on either side. Each of these sets of incisions are additionally found to be made on top of a series of horizontal thin incisions, here referred to as "baselines" that were possibly used to help guide the engraver in the creation of each set of notches.*

1.4 Holocene C.E. Notation System Samples

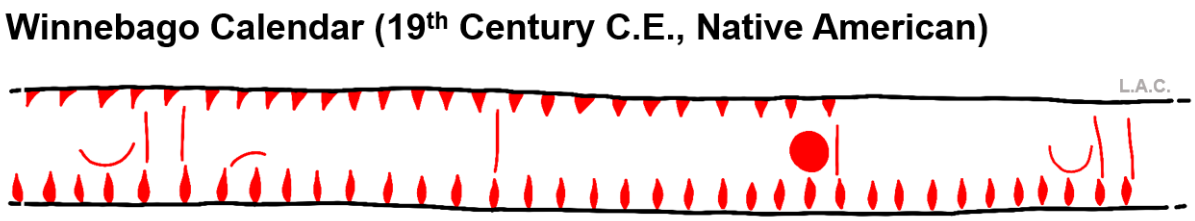

Supplementary Figure 25: *Illustrated drawing adapted from (Marshack, 1988, 1985) identifying a series of markings located on a wooden calendar stick from the 19th century, recovered from the Winnebago Native American communities.*

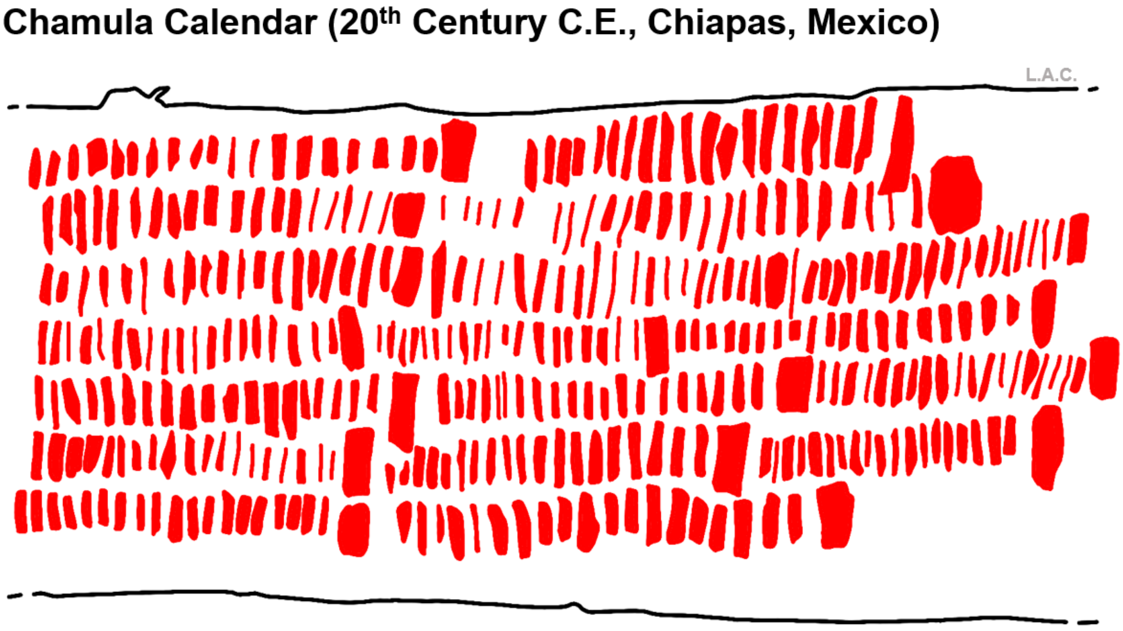

Supplementary Figure 26: *Illustrated drawing adapted from (Gossen, 1972; Marshack, 1972) identifying a series of markings located on a wooden board from the 20th century, recovered from the Chamulan township of Chiapas, Mexico.*

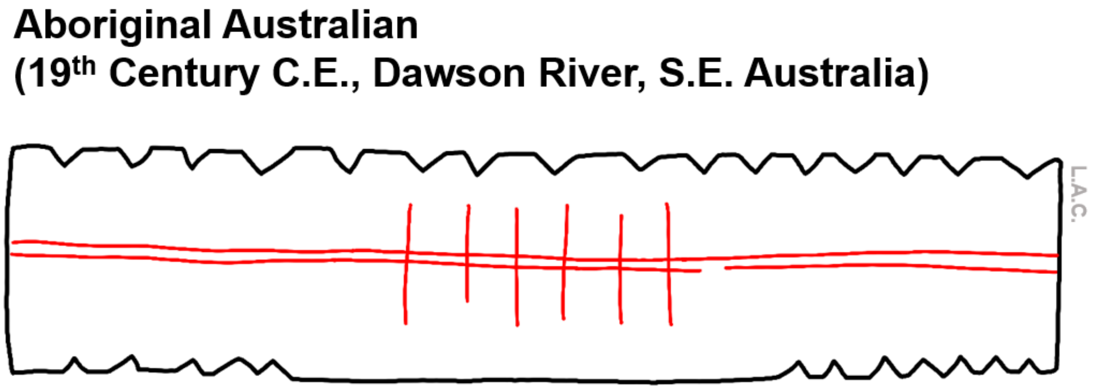

Supplementary Figure 27: *Illustrated drawing adapted from (Howitt, 1904) identifying a series of markings located on a wooden message stick from the 19th century, recovered from the Yakunbura community of the Dawson river, South-east Australia. The message stick presents a series of notches and lines, the lines indicate the number of days the messenger has traveled to deliver the stick, providing an indication to the receiver how long it will take to travel from one place to the other. The notches are to remind the messenger of different parts of the message (ibid).*

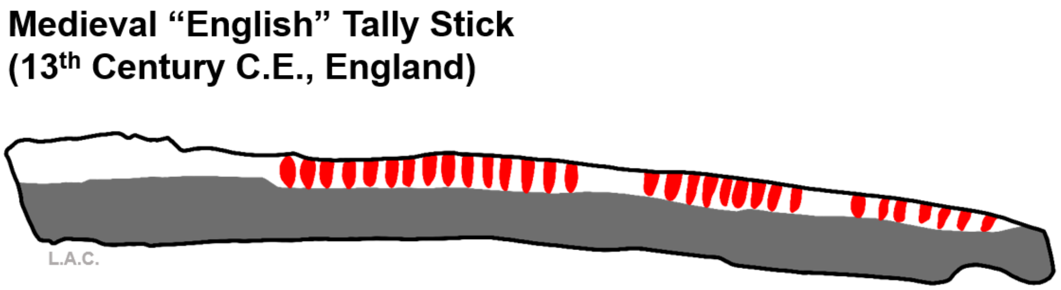

Supplementary Figure 28: *Illustrated drawing adapted from (Ifrah, 1985) identifying a series of markings located on a wooden tally stick from the XIIIth century in England. Documentation infers that these items were used to record income, expenditures, and taxes up until the 19th century at least.*

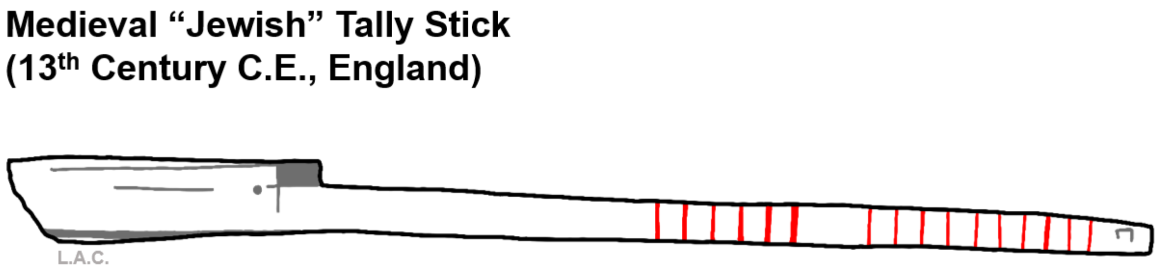

Supplementary Figure 29: *Illustrated drawing adapted from (Jenkinson, 1925) identifying a series of markings located on a wooden tally stick from the XIIIth century in England, reported and published as a Jewish Tally Stick from the Exchequer tally stocks.*

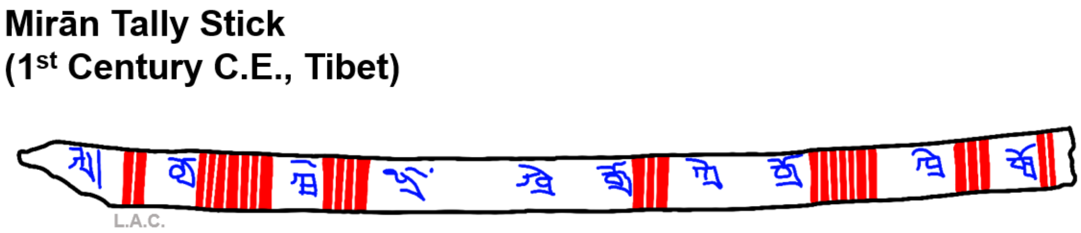

Supplementary Figure 30: *Illustrated drawing adapted from (Tas, 1956) identifying a series of markings located on a wooden tally stick from the approximately 1st millenium CE, near the city of Mirān, Tibet. These sticks bear two words among the notches, the first meaning wheat, and the second millet. Each incision next to the words supposedly indicate the number of deliveries to this important trading center.*

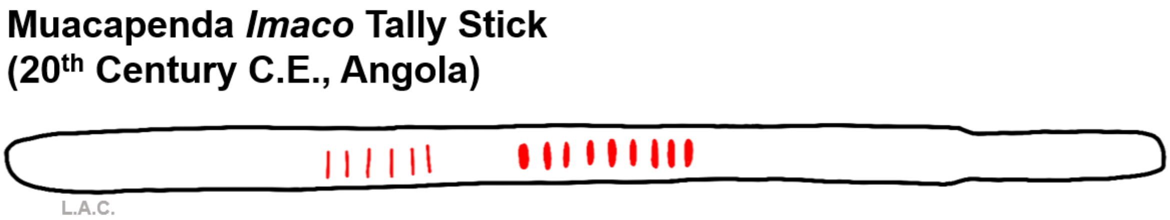

Supplementary Figure 31: *Illustrated drawing adapted from a photograph included in (Lagercrantz, 1973) presenting an example of a tally stick recovered from Cokwe in Muacapenda, Angola. This artifact is supposedly a tooth brush with a series of markings that indicate the number of days travelled or away, with the word Imaco referring to the use of the object as a means of quantifying something (Lagercrantz, 1973).*

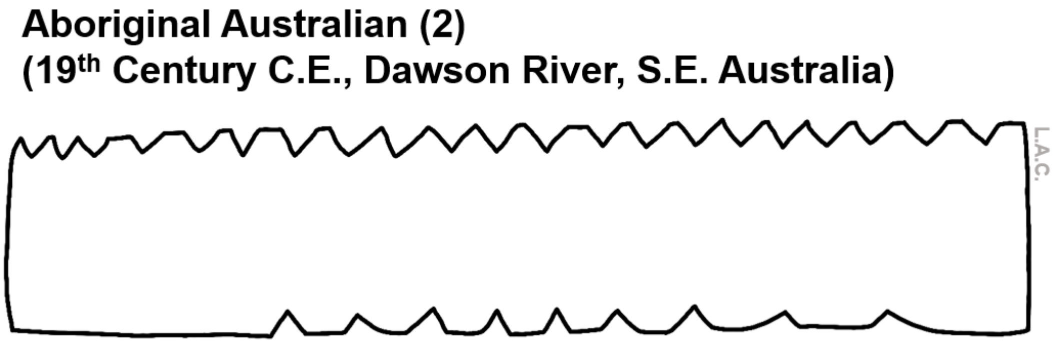

Supplementary Figure 32: *Illustrated drawing adapted from (Howitt, 1904) identifying a series of markings located on a wooden message stick from the 19th century, found in South-east Australia. The message stick presents a series of notches used to transmit a message inviting the receiver to an initiation ceremony. The notches are to remind the messenger of different parts of the message (ibid).*

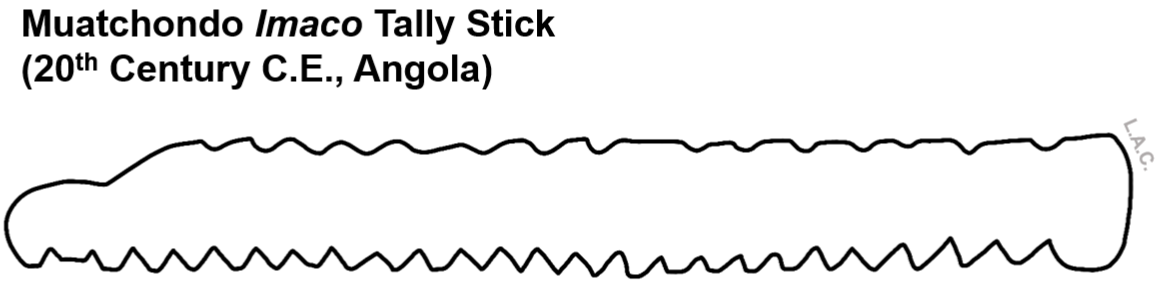

Supplementary Figure 33: *Illustrated drawing adapted from a photograph included in (Lagercrantz, 1973) presenting an example of a tally stick recovered from Muatchondo, Angola. The word Imaco refers to the use of the object as a means of quantifying something (Lagercrantz, 1973).*

2 Appendix B: Supplementary Figures ($F(r)$, $g(r)$, $K_{ij}(r)$, Rose Diagrams)

2.1 Butchery Results

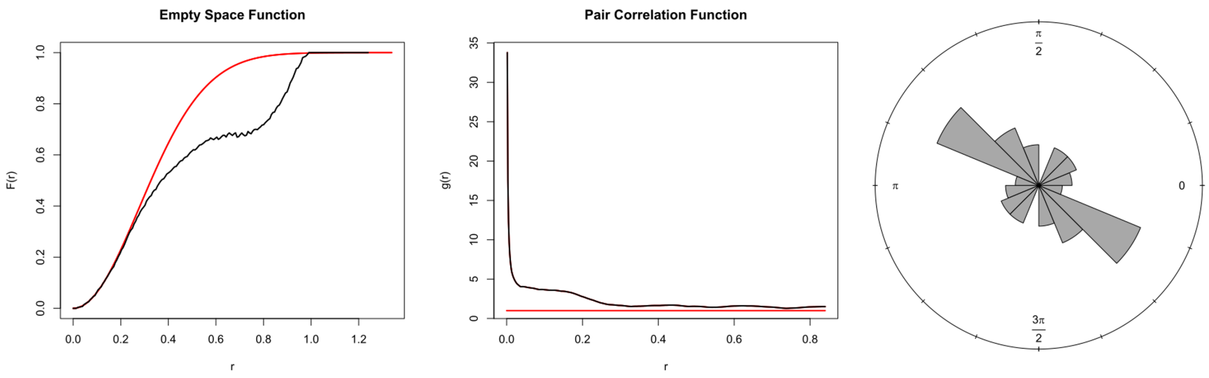

Supplementary Figure 34: *$F(r)$, $g(r)$ and Rose Plots for the spatial distribution of marks produced during experimental defleshing activities.*

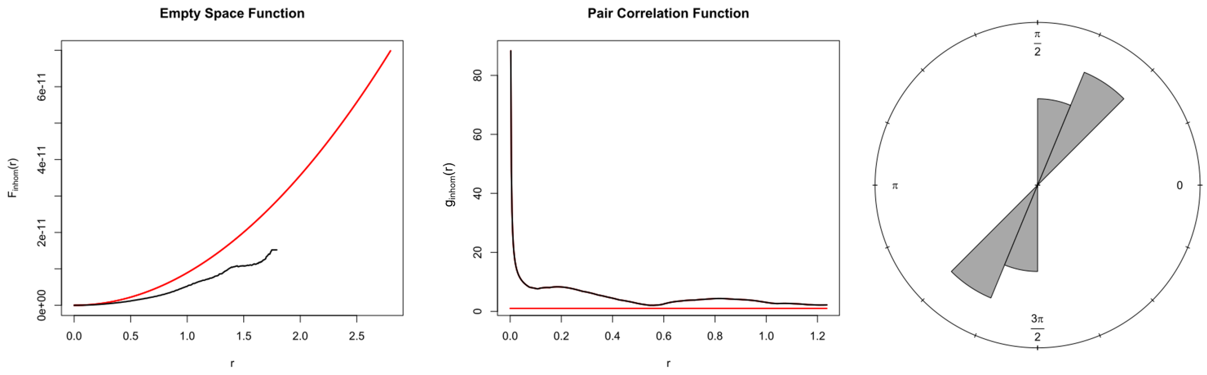

Supplementary Figure 35: *$F_{inhom}(r)$, $g_{inhom}(r)$ and Rose Plots for the spatial distribution of marks produced during experimental disarticulation activities.*

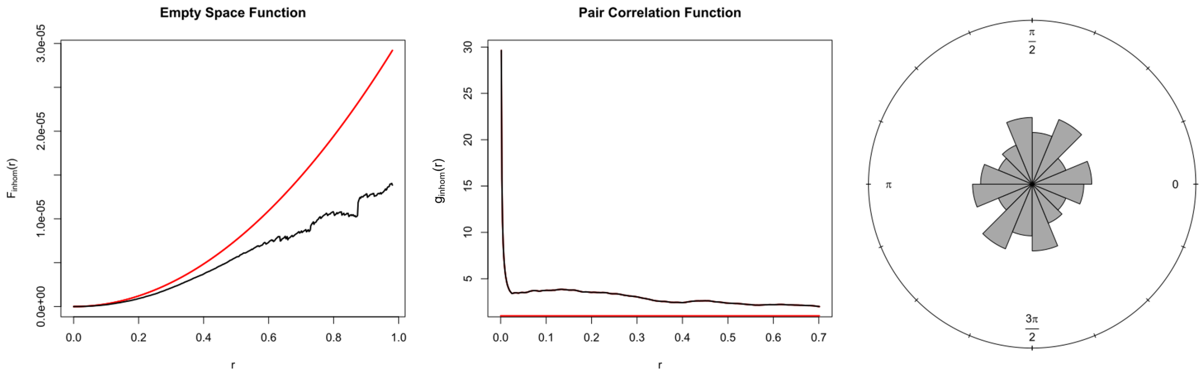

Supplementary Figure 36: *$F_{inhom}(r)$, $g_{inhom}(r)$ and Rose Plots for the spatial distribution of marks produced during experimental tendon removal activities.*

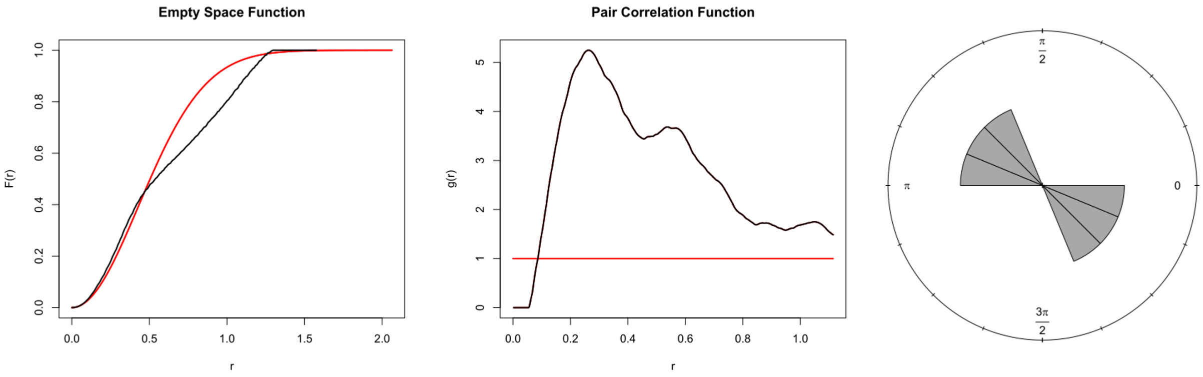

Supplementary Figure 37: *$F(r)$, $g(r)$ and Rose Plots for the spatial distribution of butchery marks observed on a bone recovered from the Lower Pleistocene site of FLK-West (Olduvai Gorge, Tanzania).*

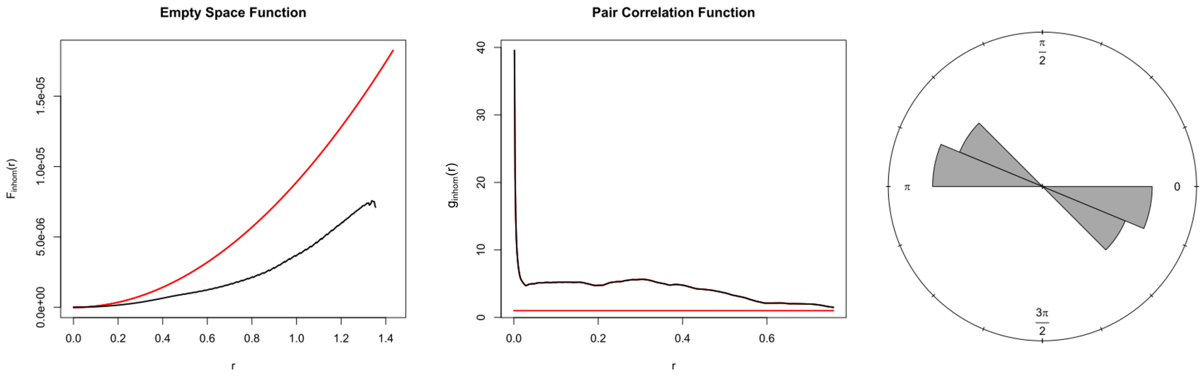

Supplementary Figure 38: *$F_{inhom}(r)$, $g_{inhom}(r)$ and Rose Plots for the spatial distribution of butchery marks observed on a bone recovered from the Middle Pleistocene site of Gran Dolina (Atapuerca, Spain).*

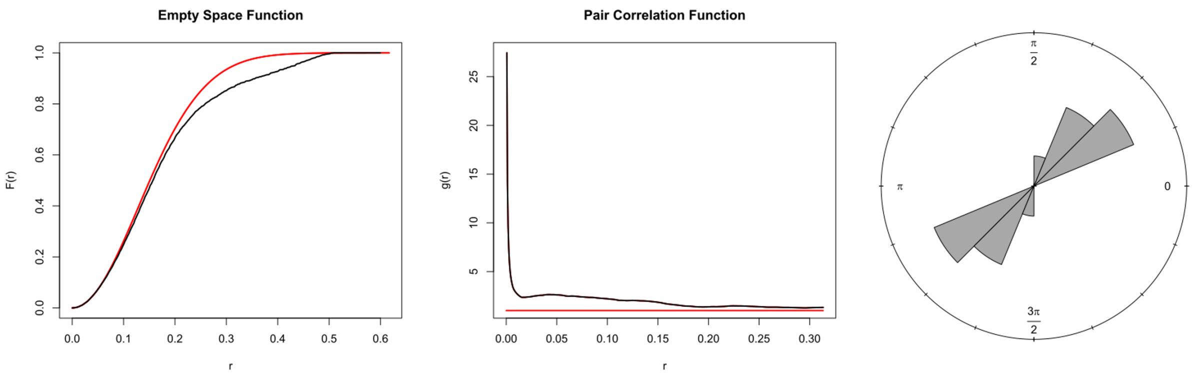

Supplementary Figure 39: *$F(r)$, $g(r)$ and Rose Plots for the spatial distribution of butchery marks observed on a bone recovered from the Upper Pleistocene site of Coimbre (Spain).*

2.2 Decorative / Depiction / Artistic Results

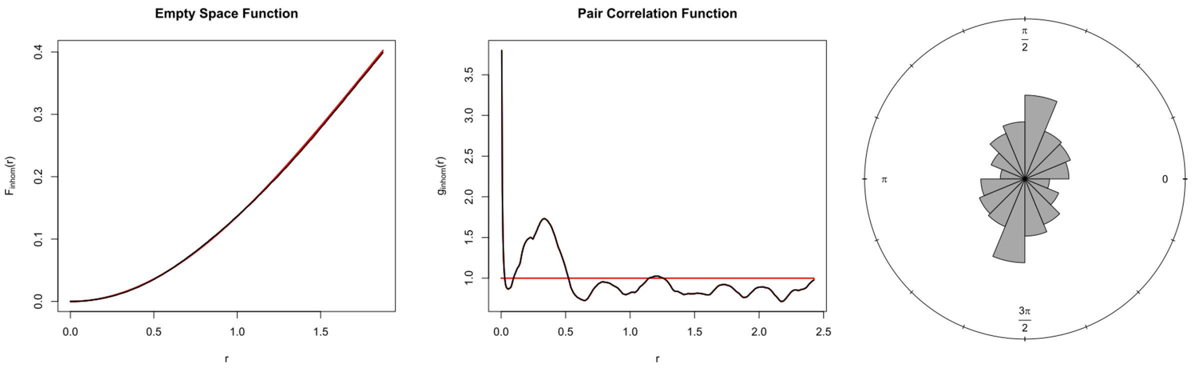

Supplementary Figure 40: *$F_{inhom}(r)$, $g_{inhom}(r)$ and Rose Plots for the spatial distribution of decorative engravings depicting a mammoth observed on a fragment of mammoth ivory recovered from the site of Le Madeleine (France).*

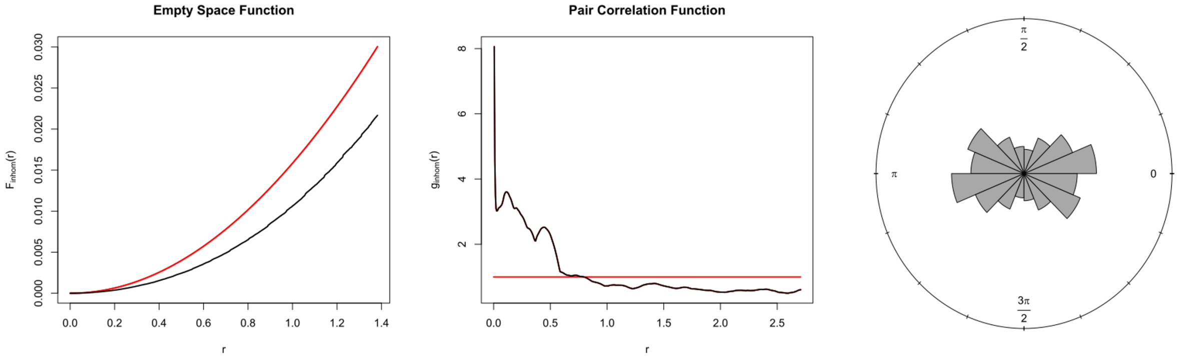

Supplementary Figure 41: *$F_{inhom}(r)$, $g_{inhom}(r)$ and Rose Plots for the spatial distribution of decorative engravings depicting two horses observed on a fragment of reindeer antler recovered from La Marche (France). Note how the $F_{inhom}(r)$ curves appear to fall below the Poisson line, however AUPC values are able to confirm that the scale and degree of this deviation from the Poisson line is unimportant (AUPC = 3.7×10^{-4}).*

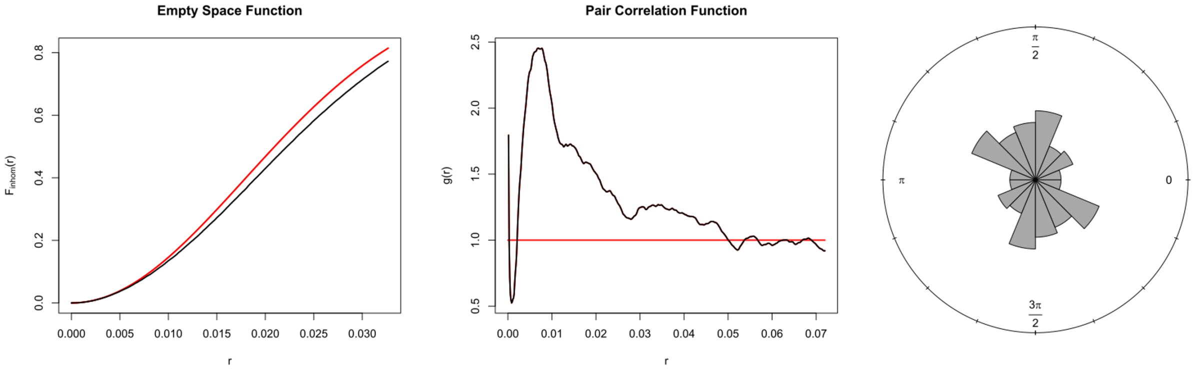

Supplementary Figure 42: *$F(r)$, $g(r)$ and Rose Plots for the spatial distribution of decorative engravings depicting a deer observed on a fragment of perforated bone in the shape of a disc recovered from Laugerie Basse (France).*

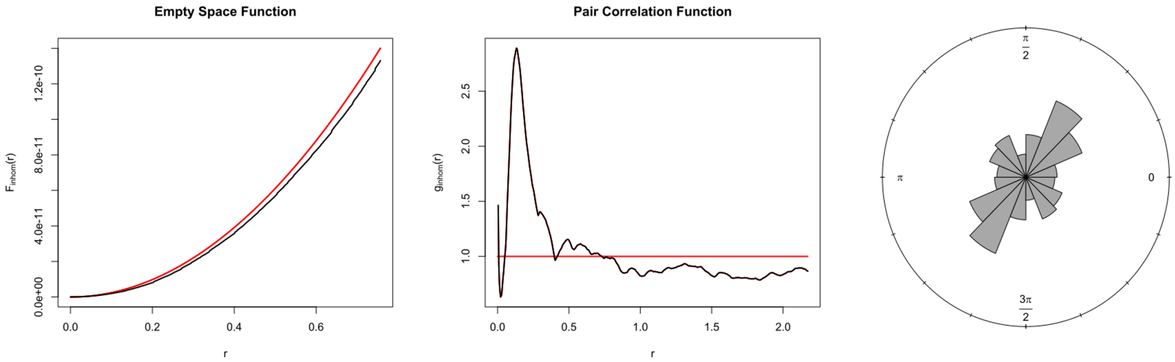

Supplementary Figure 43: *$F_{inhom}(r)$, $g_{inhom}(r)$ and Rose Plots for the spatial distribution of decorative geometric engravings on a fragment of deer antler recovered from Zigeunerhohle (Austria).*

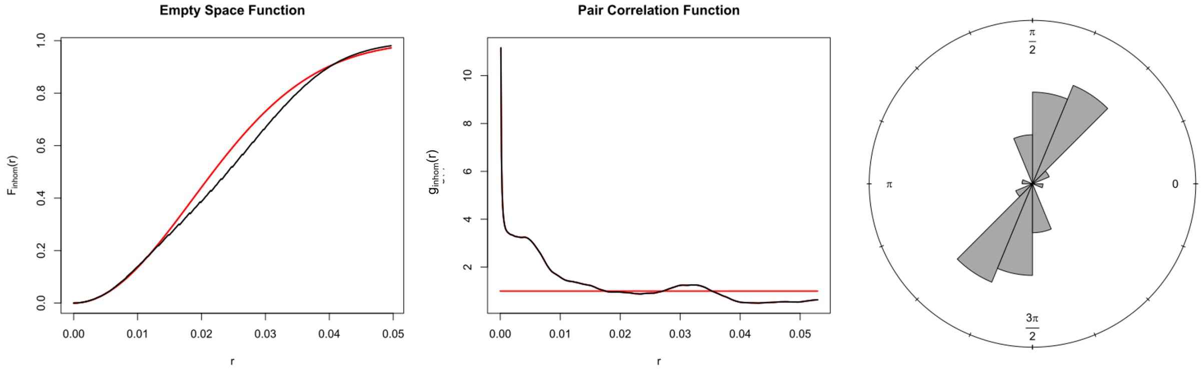

Supplementary Figure 44: *$F_{inhom}(r)$, $g_{inhom}(r)$ and Rose Plots for the spatial distribution of decorative geometric engravings on a lissoir recovered from Brassempouy (France).*

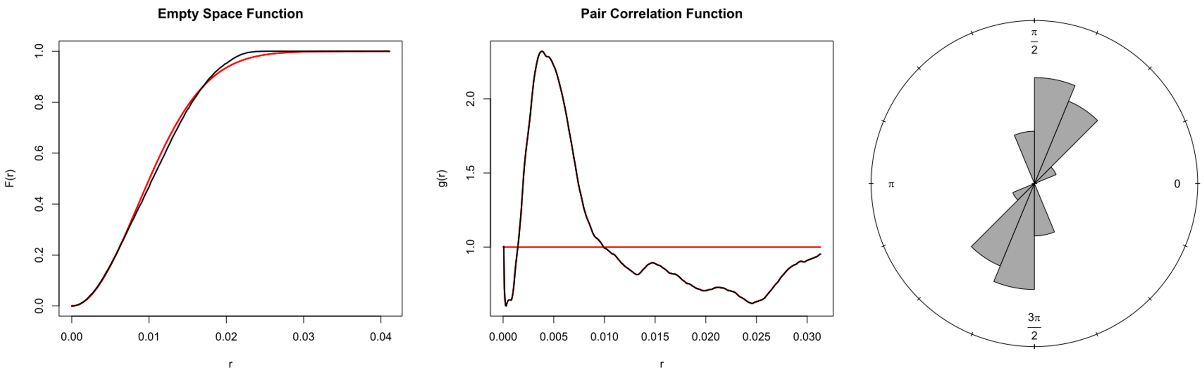

Supplementary Figure 45: *$F(r)$, $g(r)$ and Rose Plots for the spatial distribution of decorative geometric engravings on a lissoir recovered from Isturitz (288, France).*

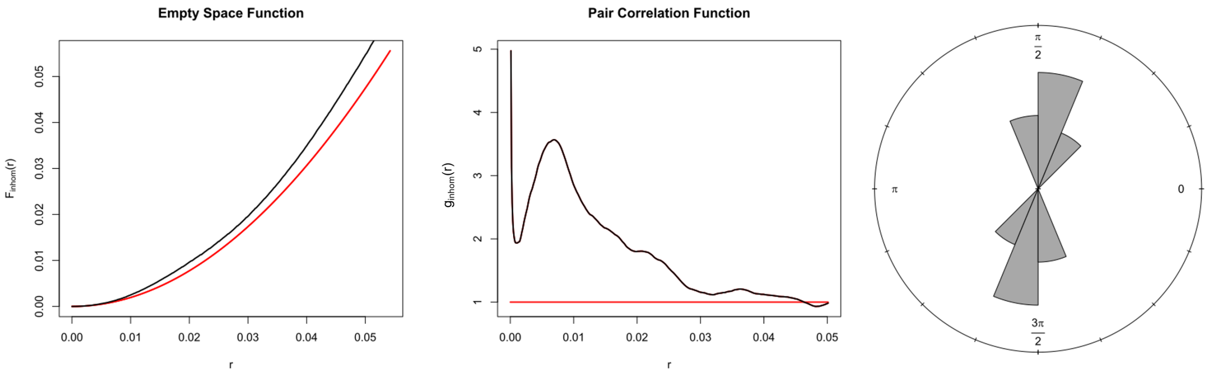

Supplementary Figure 46: *$F_{inhom}(r)$, $g_{inhom}(r)$ and Rose Plots for the spatial distribution of decorative geometric engravings on a lissoir recovered from Isturitz (284, France).*

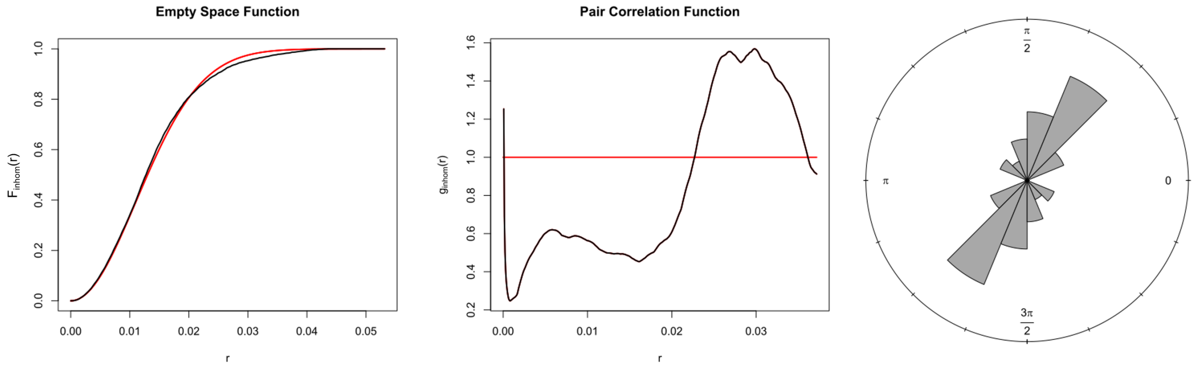

Supplementary Figure 47: *$F_{inhom}(r)$, $g_{inhom}(r)$ and Rose Plots for the spatial distribution of decorative geometric engravings on a lissoir recovered from Duruthy (France).*

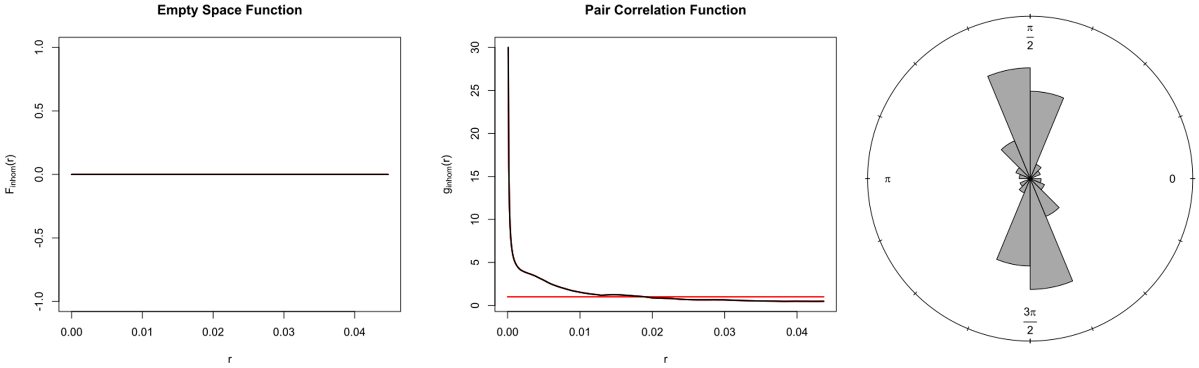

Supplementary Figure 48: *$F_{inhom}(r)$, $g_{inhom}(r)$ and Rose Plots for the spatial distribution of decorative geometric engravings on the distal fragment of human radius recovered from Gough's Cave (England).*

2.3 Potential Pleistocene AMS Results

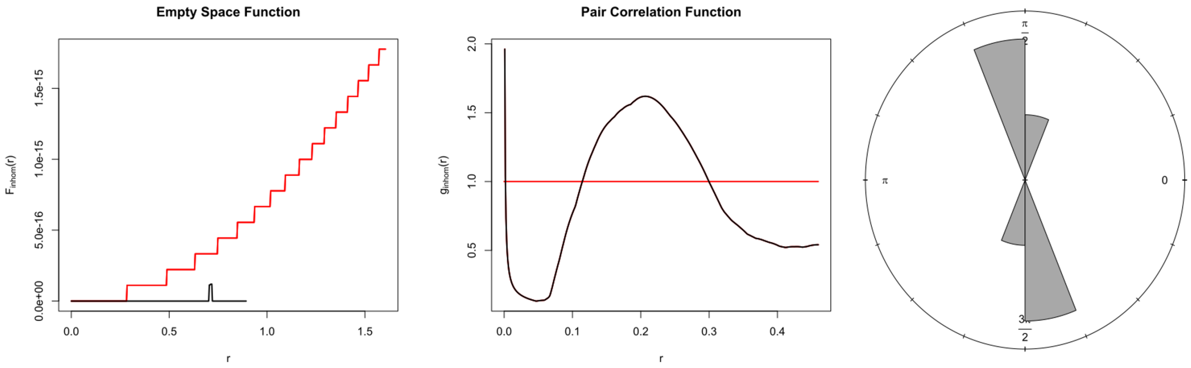

Supplementary Figure 49: *$F_{inhom}(r)$, $g_{inhom}(r)$ and Rose Plots for the spatial distribution of notches depicting a possible Artificial Memory System on a fragment of rib recovered from Blanchard (D38.23.1858, France).*

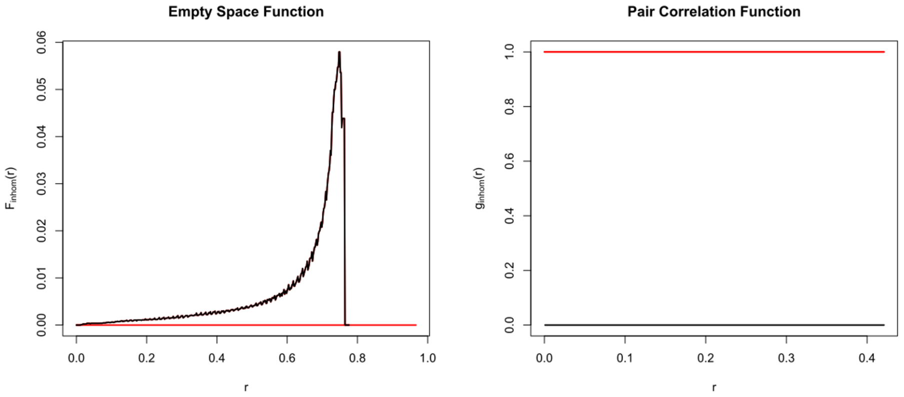

Supplementary Figure 50: *$F_{inhom}(r)$ and $g_{inhom}(r)$ for the spatial distribution of notches depicting a possible Artificial Memory System on a fragment of rib recovered from Blanchard (D38.23.1958, France).*

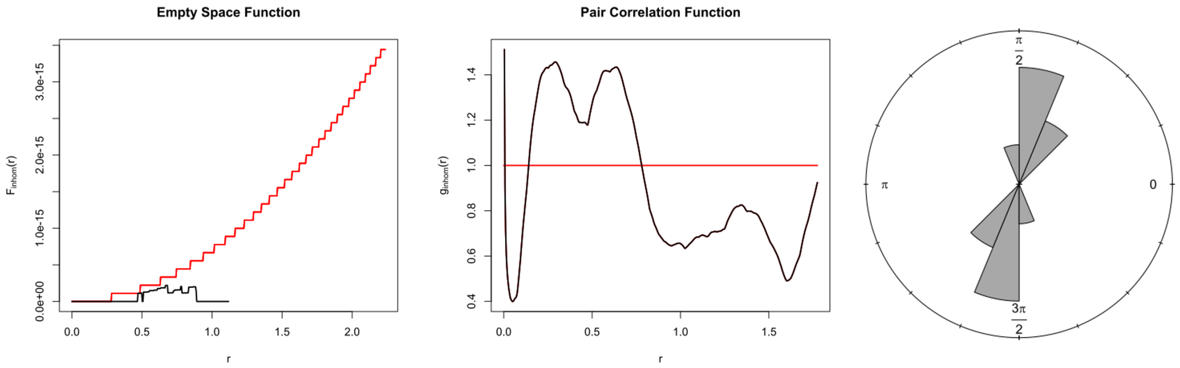

Supplementary Figure 51: *$F_{inhom}(r)$ and $g_{inhom}(r)$ for the spatial distribution of notches depicting a possible Artificial Memory System on a fragment of rib recovered from Solutré (France).*

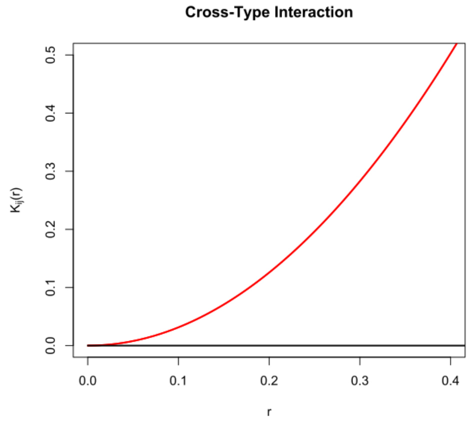

Supplementary Figure 52: *$K_{ij}(r)$ results for cross-type interactions between the notches identified as possible AMSs on the rhino rib from Solutré and the rest of the engravings (possibly more decorative) observed on this side of the rib.*

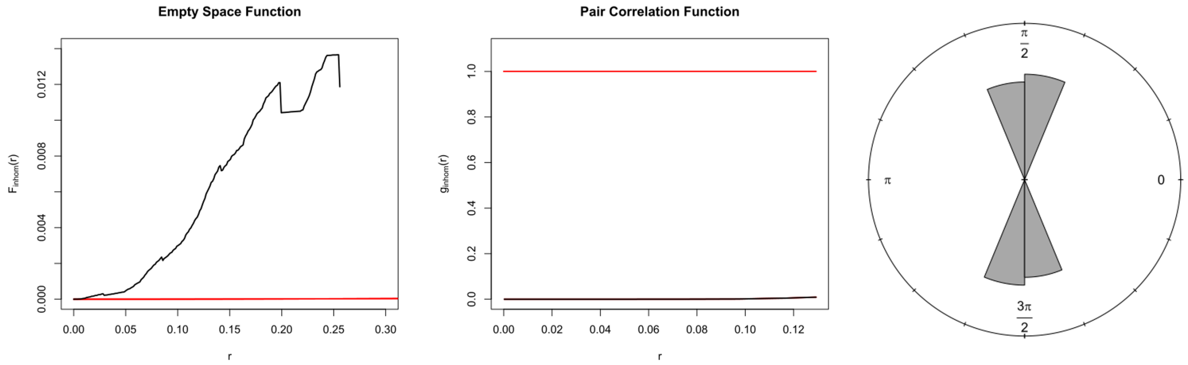

Supplementary Figure 53: *$F_{inhom}(r)$ and $g_{inhom}(r)$ for the spatial distribution of notches depicting a possible Artificial Memory System on the metapodial of a reindeer recovered from Labattut (France).*

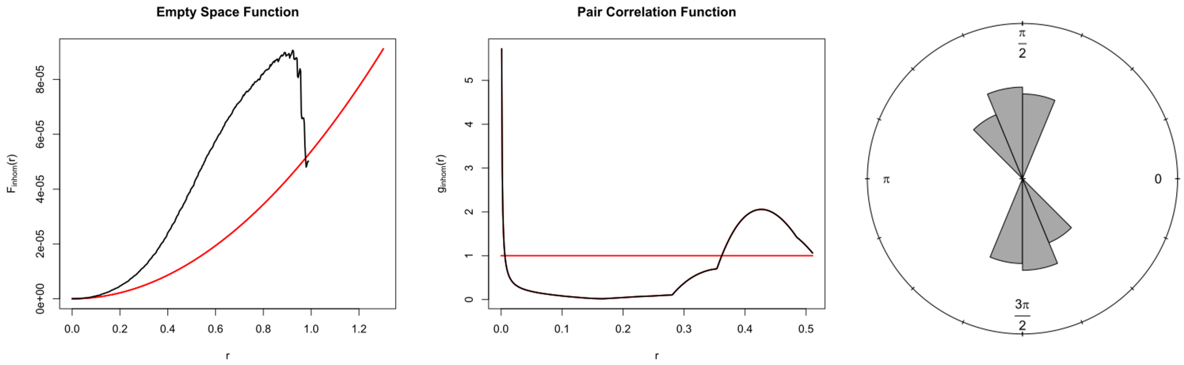

Supplementary Figure 54: *$F_{inhom}(r)$ and $g_{inhom}(r)$ for the spatial distribution of notches depicting a possible Artificial Memory System on the femur of a hyena recovered from Les Pradelles (France).*

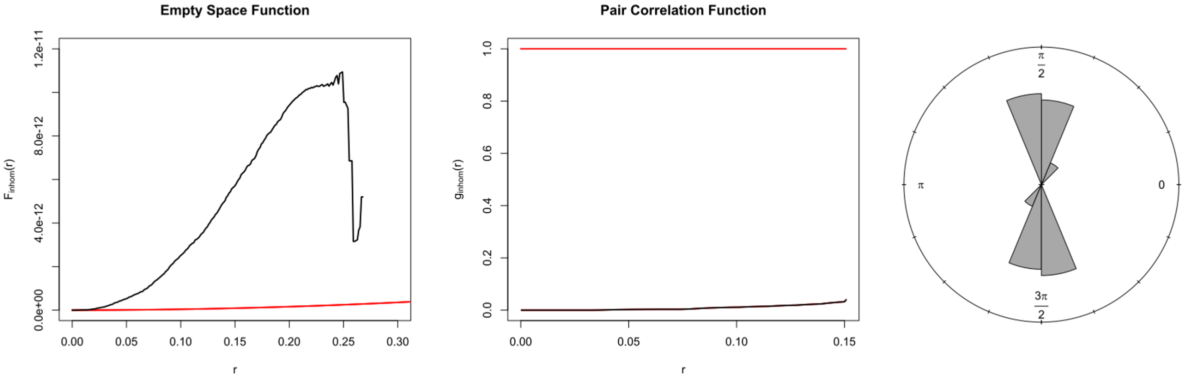

Supplementary Figure 55: *$F_{inhom}(r)$ and $g_{inhom}(r)$ for the spatial distribution of notches depicting a possible Artificial Memory System on the fibula of a baboon recovered from Border Cave (South Africa).*

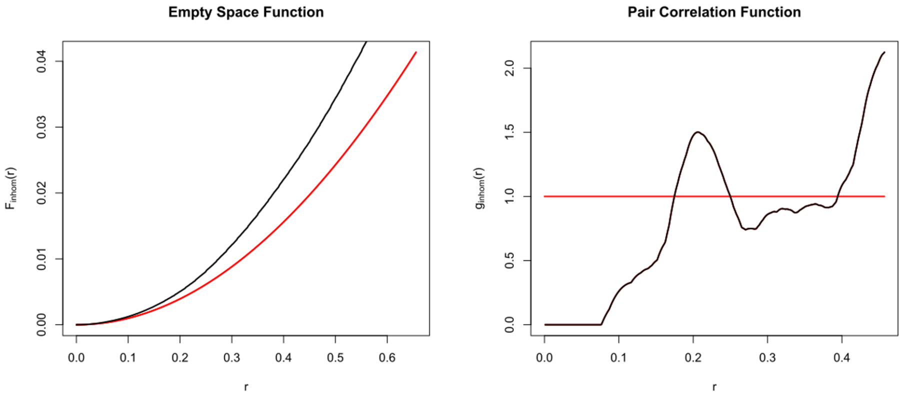

Supplementary Figure 56: *$F_{inhom}(r)$ and $g_{inhom}(r)$ for the spatial distribution of markings depicting a possible Artificial Memory System on the La Marche Antler (France).*

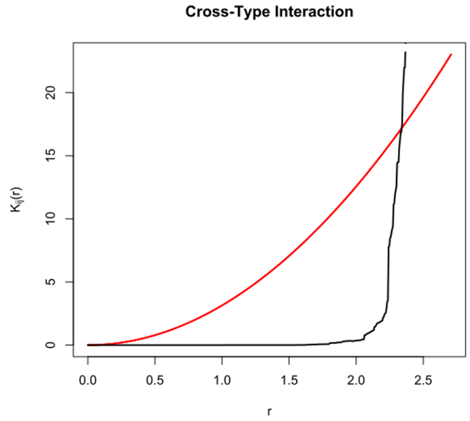

Supplementary Figure 57: *$K_{ij}(r)$ results for cross-type interactions between the markings identified as possible AMSs on the La Marche Antler, and the decorative and figurative engravings of horses observed on the rest of the angler.*

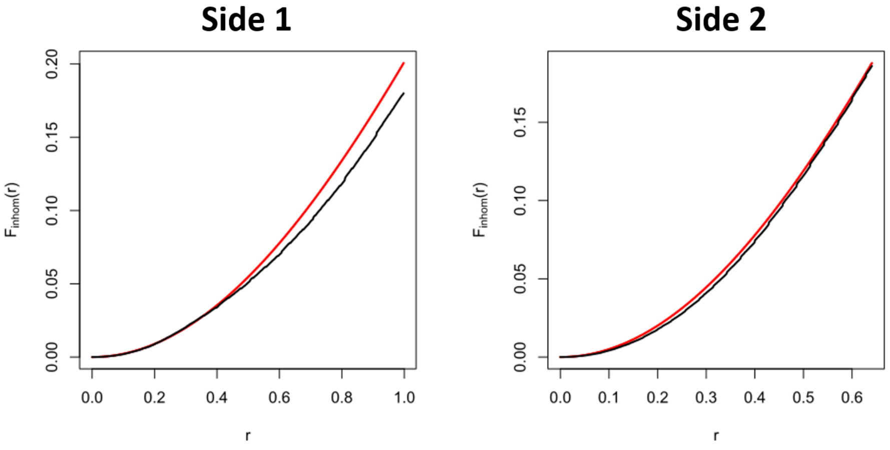

Supplementary Figure 58: *$F_{inhom}(r)$ results for each of the sides of the La Marche antler separately.*

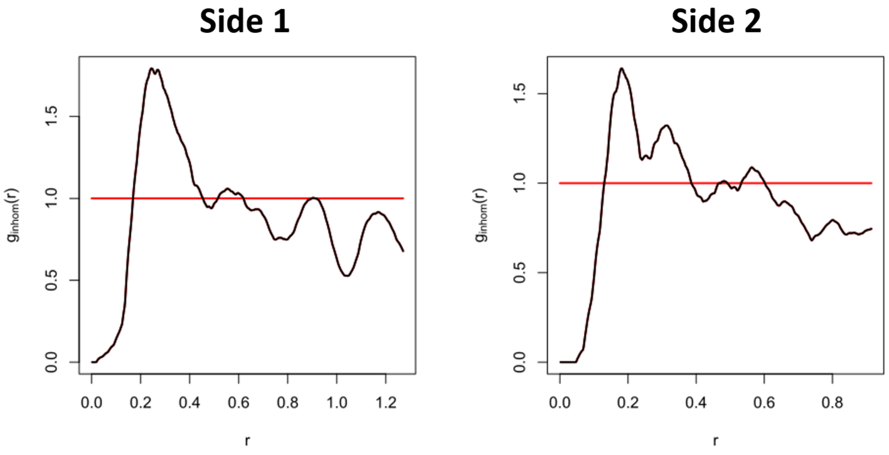

Supplementary Figure 59: $g_{inhom}(r)$ *results for each of the sides of the La Marche antler separately.*

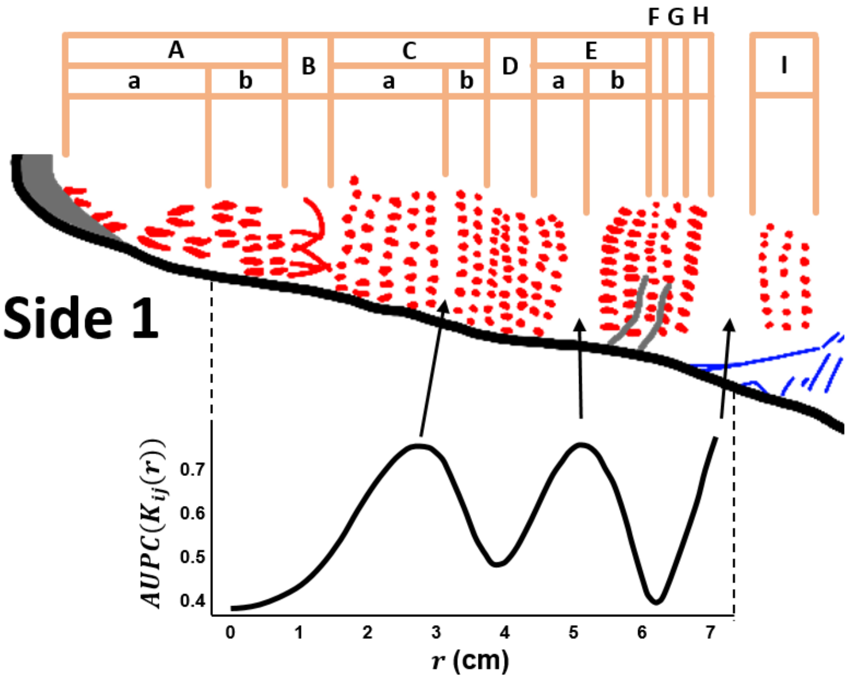

Supplementary Figure 60: *Figurative graph demonstrating the AUPC values for* $K_{ij}(r)$ *functions when assessing the cross-type interactions between different subsets and sets across Side 1 of the La Marche antler. Subsets are defined according to D'Errico (1995). The spatial separation and cross-type interactions between each of the sets and subsets shows interesting patterns for certain degrees of mild to more extreme inhibition, yet never present patterns of attraction. Here it can be seen how* $K_{ij}(r)$ *values vary when comparing neighbouring sets along the length of Face 1. Evidently 3 strong piques occur separating sets Ca and Cb, sets Ea and Eb, as well as set H with set I. While visually the separation between sets Ca and Cb appears to be the smallest, statistically this division of space is just as important as the case of Ea with Eb. When considering Set 2, not all sets have sample sizes large enough to calculate these values, however set the AUPC of set J with set K was calculated at 0.22, set K with set L at 1.99, and finally the most marked differences are evidently between sets N and O with set P, calculated at 2.15.*

 QUANTA PROJECT

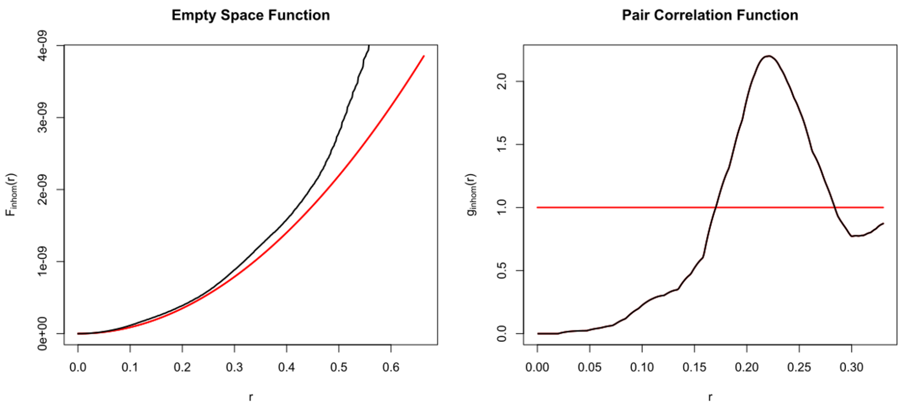

Supplementary Figure 61: *$F_{inhom}(r)$ and $g_{inhom}(r)$ for the spatial distribution of marks depicting a possible Artificial Memory System on Side 1 of the Laugerie Basse Spatula (France).*

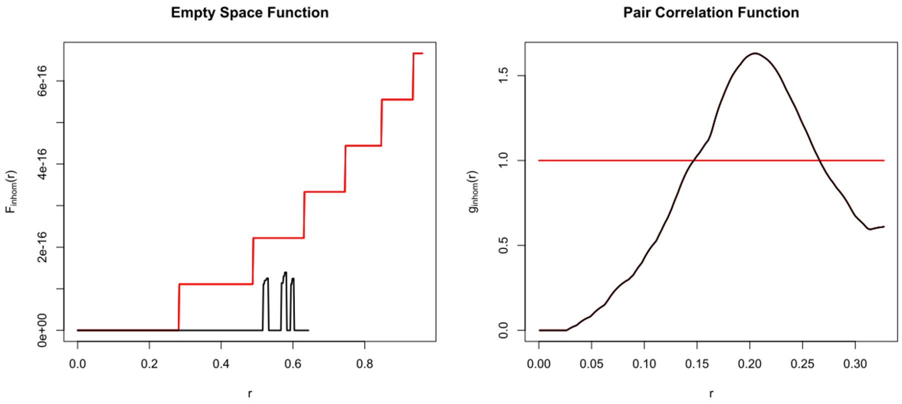

Supplementary Figure 62: *$F_{inhom}(r)$ and $g_{inhom}(r)$ for the spatial distribution of marks depicting a possible Artificial Memory System on Side 2 of the Laugerie Basse Spatula (France).*

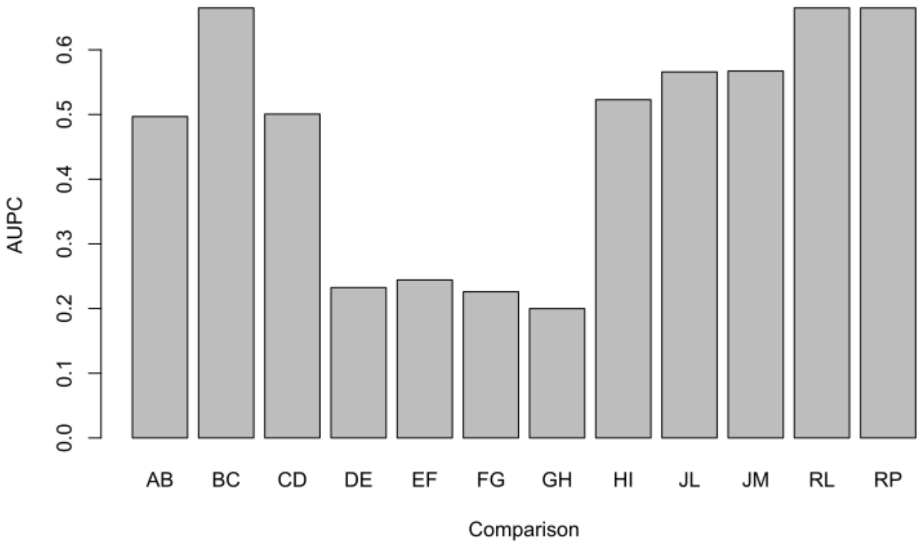

Supplementary Figure 63: *AUPC values for $K_{ij}(r)$ values calculating cross-type interactions between sets of marks on both faces of the Laugerie Basse spatula*

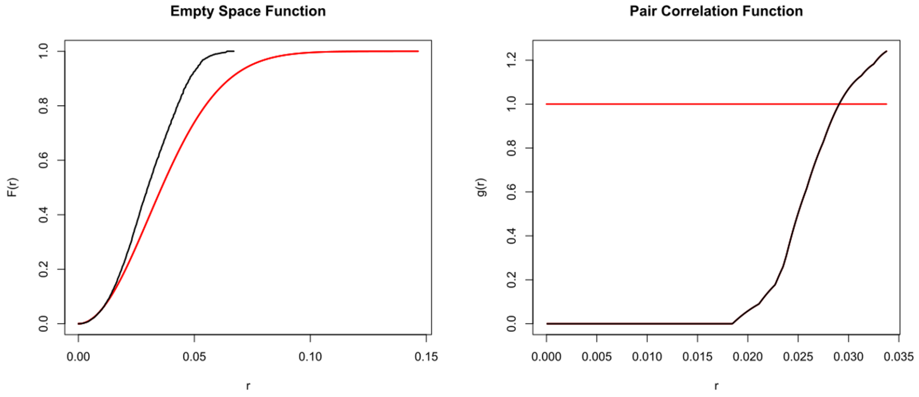

Supplementary Figure 64: *$F_{inhom}(r)$ and $g_{inhom}(r)$ for the spatial distribution of marks depicting a possible Artificial Memory System on Side 1 of the Tossal de la Roca Pendant (Spain).*

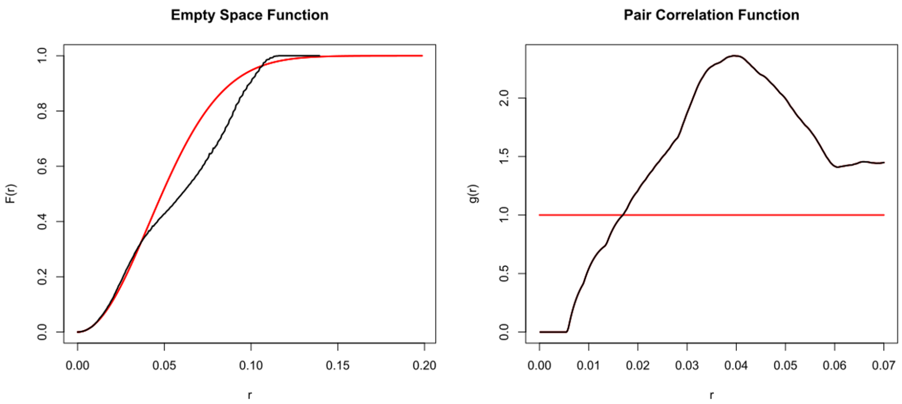

Supplementary Figure 65: $F_{inhom}(r)$ *and* $g_{inhom}(r)$ *for the spatial distribution of marks depicting a possible Artificial Memory System on Side 2 of the Tossal de la Roca Pendant (Spain).*

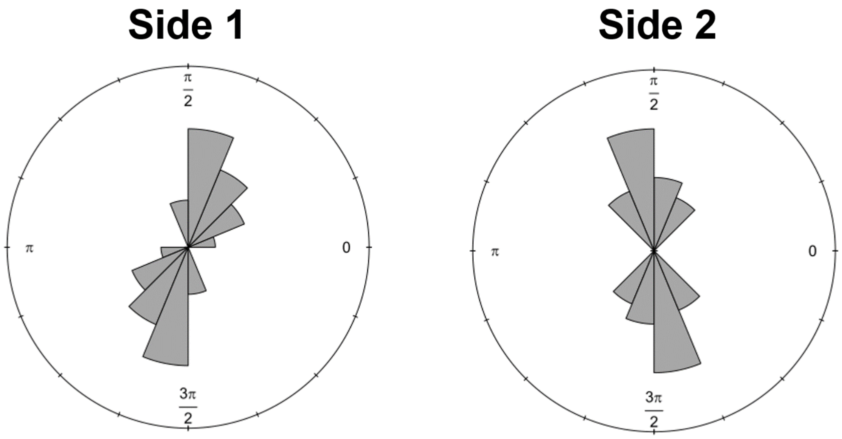

Supplementary Figure 66: *Rose diagrams for both side of the possible Artificial Memory System from Tossal de la Roca (Spain).*

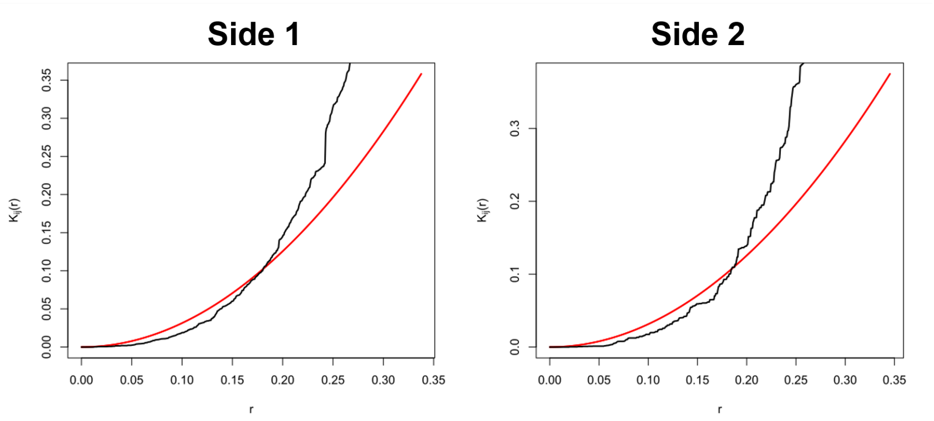

Supplementary Figure 67: *$K_{ij}(r)$ values assessing cross-type interactions between the baseline markings and the main overlapping notches on both faces of the pendant from Tossal de la Roca. Note: Two different patterns emerge depending on the scale being assessed. For smaller values of r ($r < 0.175$ cm) inhibition is present for both face A (AUPC = 0.018) and face B (AUPC = 0.011). Values above this, however, see a drastic increase in $K_{ij}(r)$ values for both faces indicating attraction between the baseline and the overlying engravings (AOPC Face A = 0.26, Face B = 0.22), thus statistically and spatially confirming the observations by D'Errico and Cacho (1994). Considering AUPC and AOPC values, however, it can be argued that attraction is the most prevalent spatial pattern.*

2.4 Holocene C.E. Notation System Results

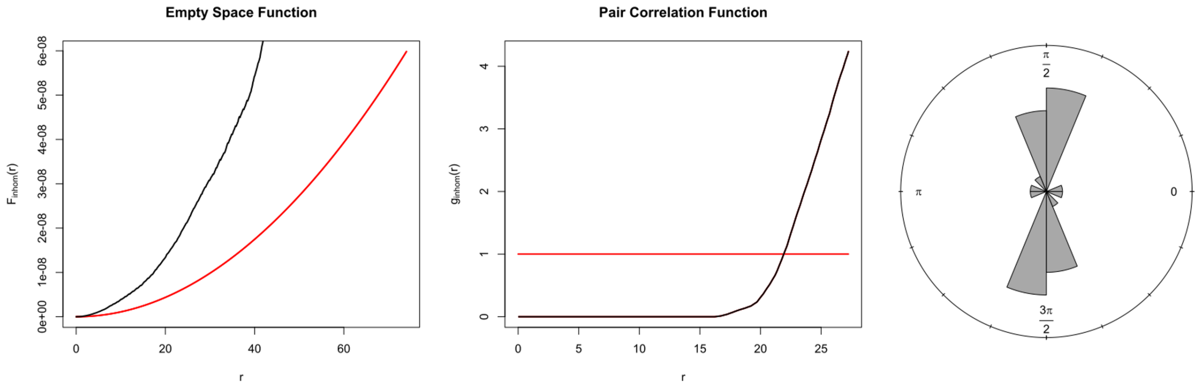

Supplementary Figure 68: *$F_{inhom}(r)$, $g_{inhom}(r)$ and Rose Plots for the spatial distribution of the markings found on the Winnebago Calendar (North America).*

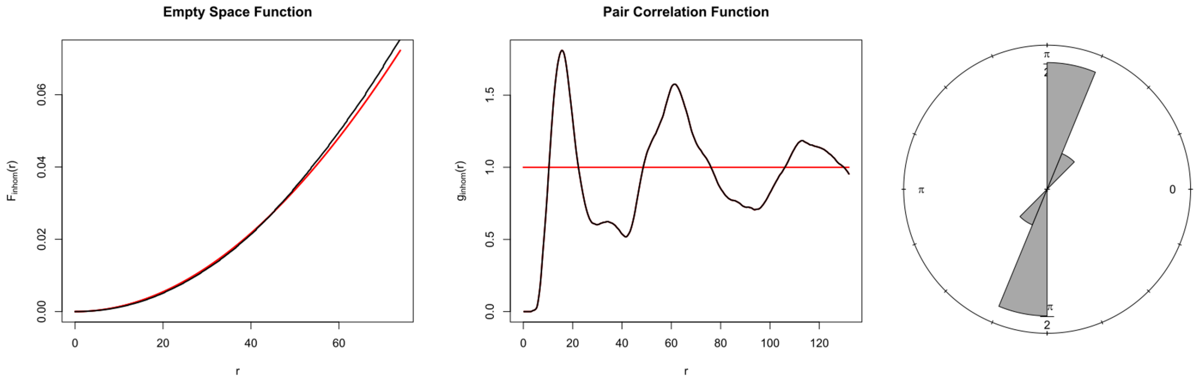

Supplementary Figure 69: *$F_{inhom}(r)$, $g_{inhom}(r)$ and Rose Plots for the spatial distribution of the markings found on the Chamula Calendar (Chiapas, Mexico).*

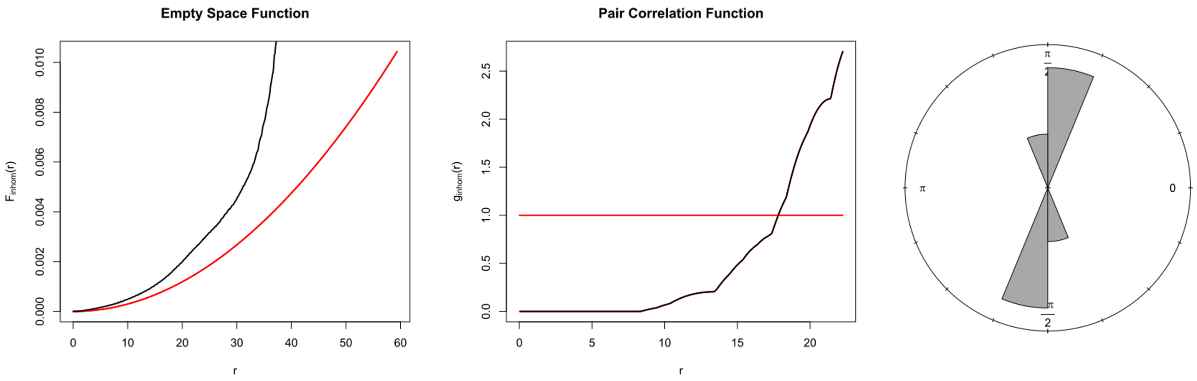

Supplementary Figure 70: *$F_{inhom}(r)$, $g_{inhom}(r)$ and Rose Plots for the spatial distribution of the markings found on the Aboriginal Australian message stick (Dawson River, S.E. Australia).*

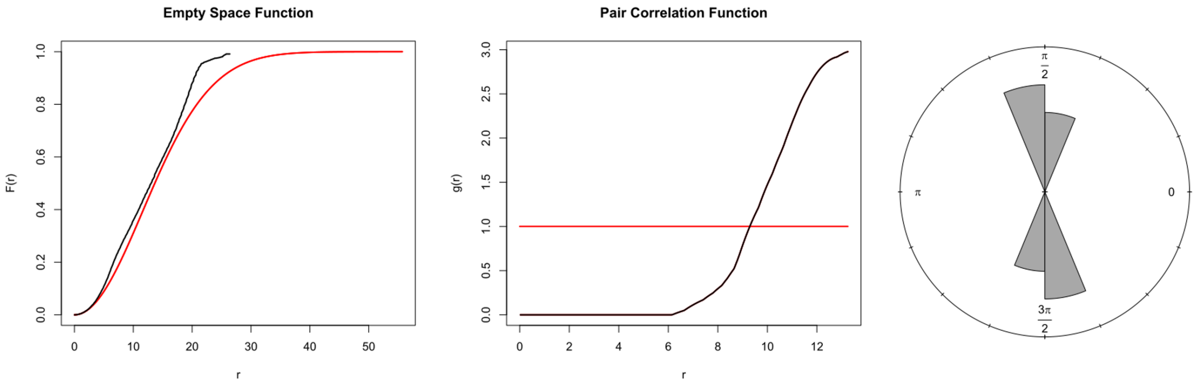

Supplementary Figure 71: *$F(r)$, $g(r)$ and Rose Plots for the spatial distribution of the markings found on the Medieval "English" Tally Stick.*

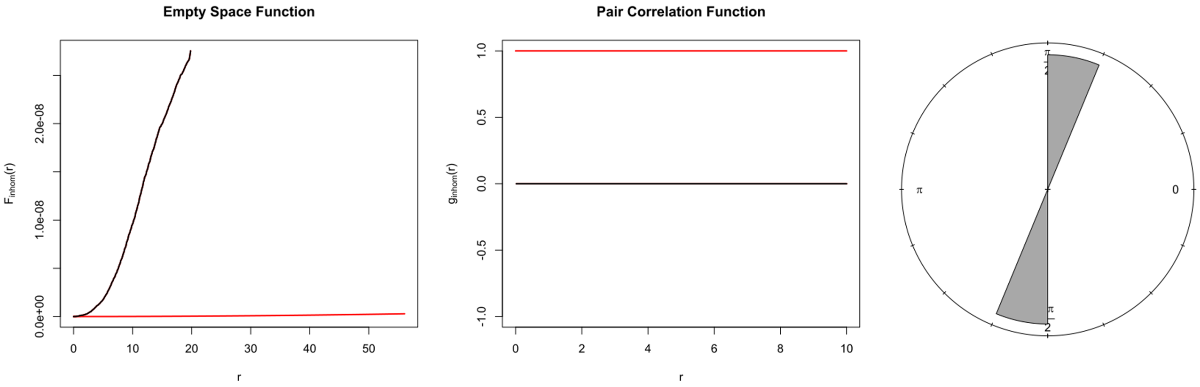

Supplementary Figure 72: *$F_{inhom}(r)$, $g_{inhom}(r)$ and Rose Plots for the spatial distribution of the markings found on the Medieval "Jewish" Tally Stick.*

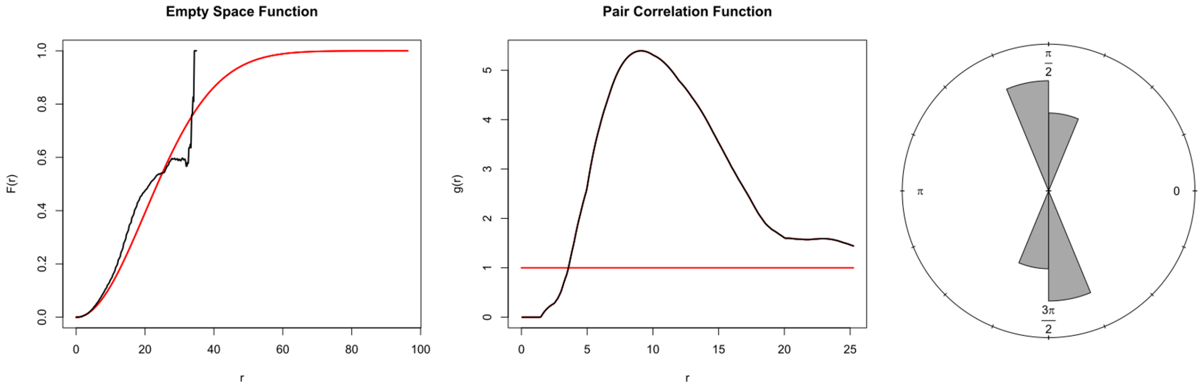

Supplementary Figure 73: *$F_{inhom}(r)$, $g_{inhom}(r)$ and Rose Plots for the spatial distribution of the markings found on the Mirān Tally Stick.*

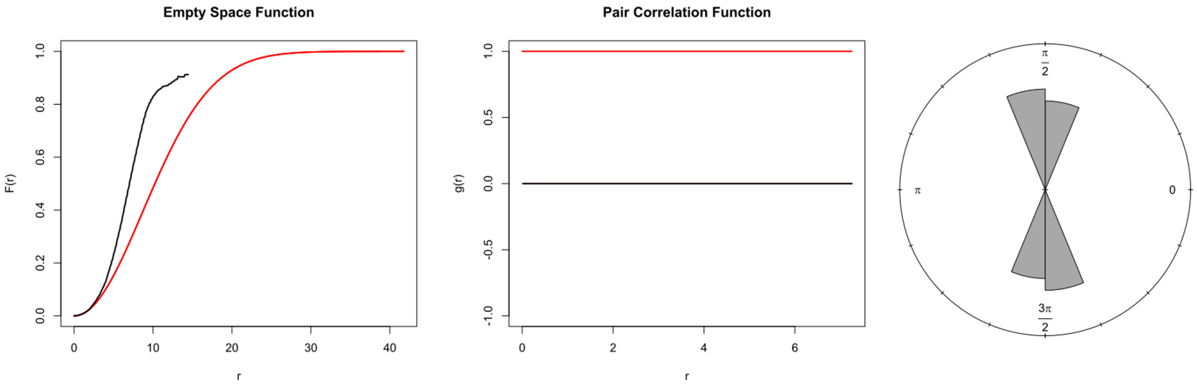

Supplementary Figure 74: *$F_{inhom}(r)$, $g_{inhom}(r)$ and Rose Plots for the spatial distribution of the markings found on the Muacapenda Tally Stick.*

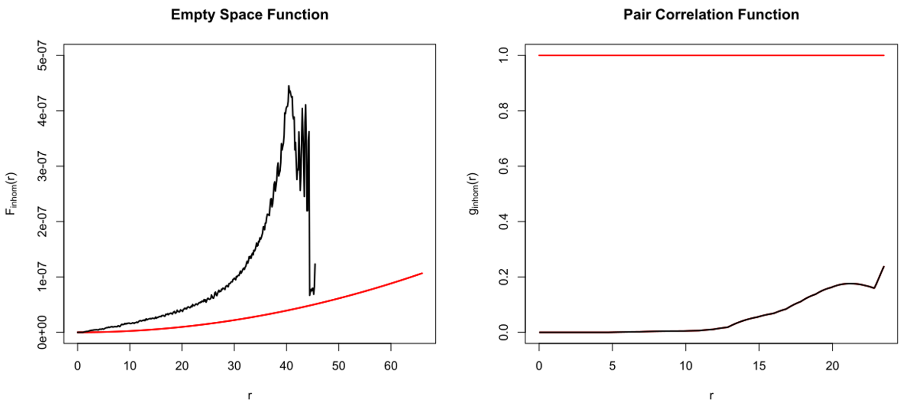

Supplementary Figure 75: $F_{inhom}(r)$, $g_{inhom}(r)$ *and Rose Plots for the spatial distribution of the markings found on the Aboriginal Australian (2) message stick (Dawson River, S.E. Australia).*

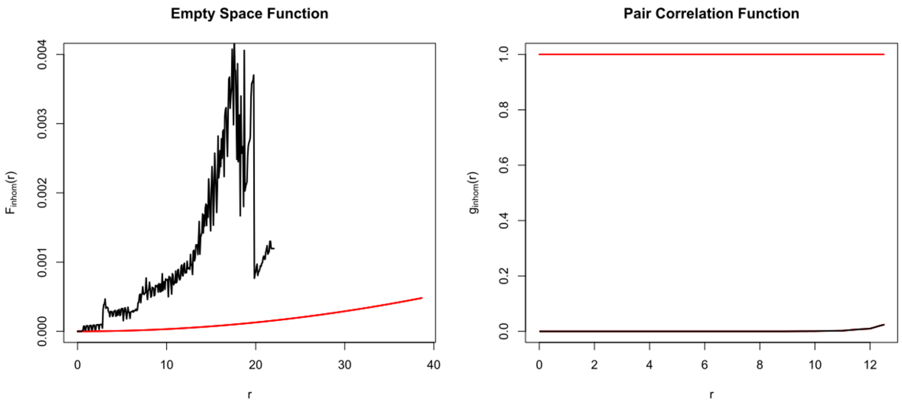

Supplementary Figure 76: $F_{inhom}(r)$, $g_{inhom}(r)$ *and Rose Plots for the spatial distribution of the markings found on the Muatchondo Tally Stick.*

2.5 Additional Results

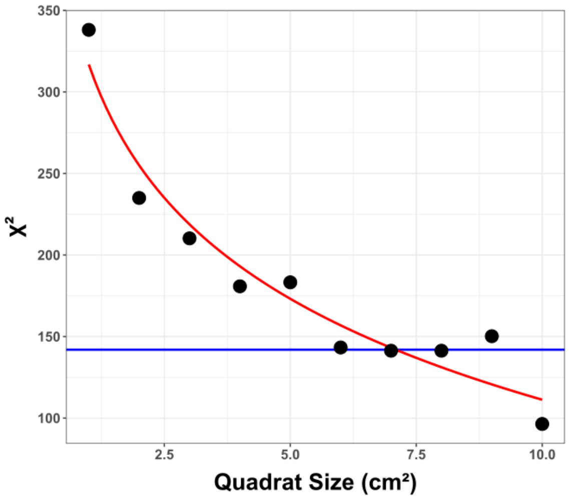

Supplementary Figure 77: *Plot of different quadrat sizes and the corresponding χ^2 statistic. Red line indicates the exponential regression line, which fit this data better (AIC = 89.8, r^2 = 0.94), than a linear model (AIC = 102.0, r^2 = 0.79). Blue line indicates the relative cut-off of greatest statistical stability.*

3 Appendix C: Supplementary Figures (PCAs and Cluster Plots)

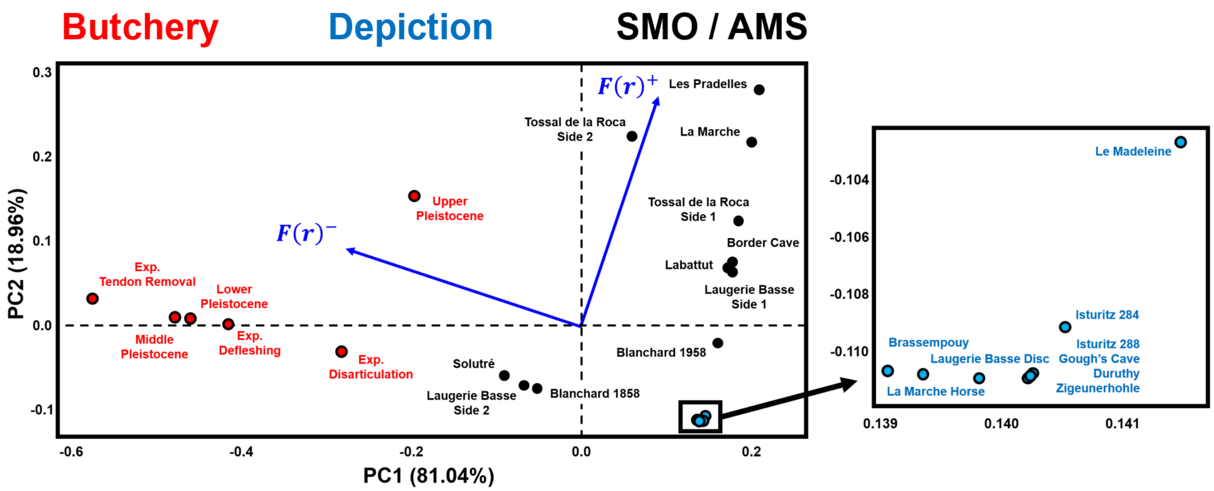

Supplementary Figure 78: *Principal Component Analysis biplot comparing butchery, Depiction and potential AMS samples based on purely spatial attributes derived from $F(r)$ AUPC and AOPC values.*

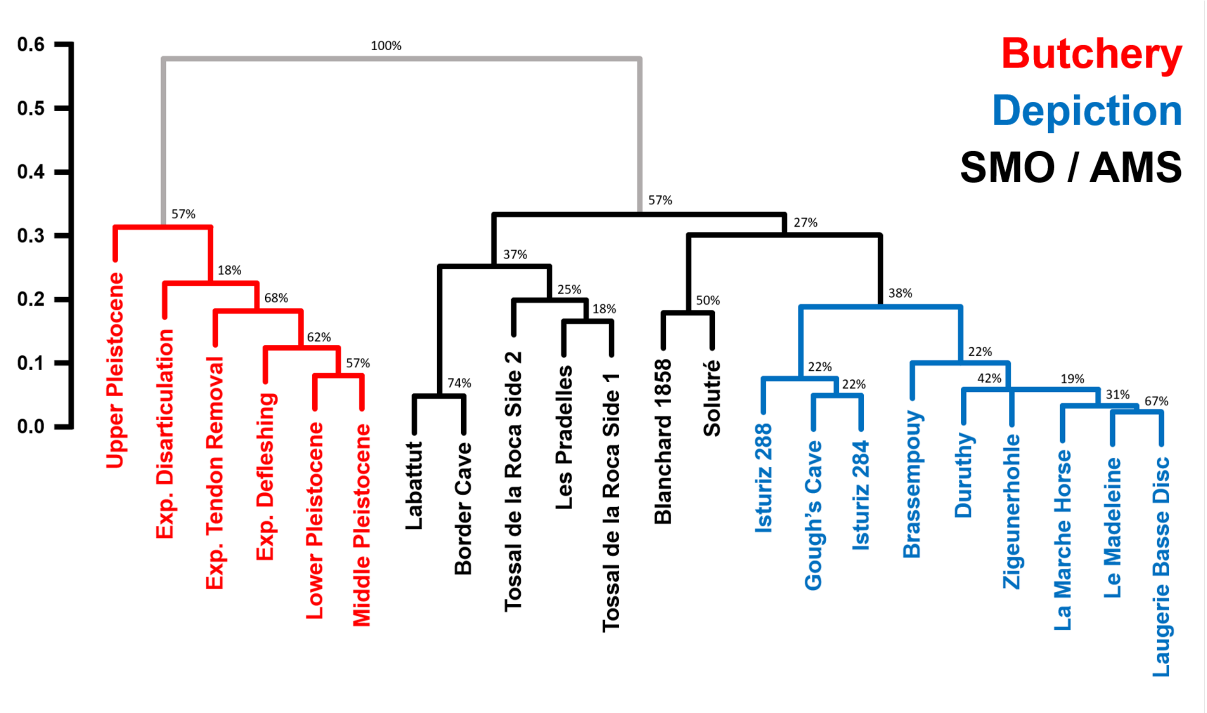

Supplementary Figure 79: *Hierarchical clustering results calculated using the Unweighted Pair-Group Method with arithmetic mean on the spatial attributes of butchery, Depiction and potential AMS samples. Euclidean distances were used to compare the samples based on both the spatial attributes derived from $F(r)$ AUPC and AOPC values, as well as the linearly transformed central tendency values for mark orientations (θ_{lin}), and the sample circular variance (v) values. Cophenetic Correlation = 0.880, AMI = 0.756*

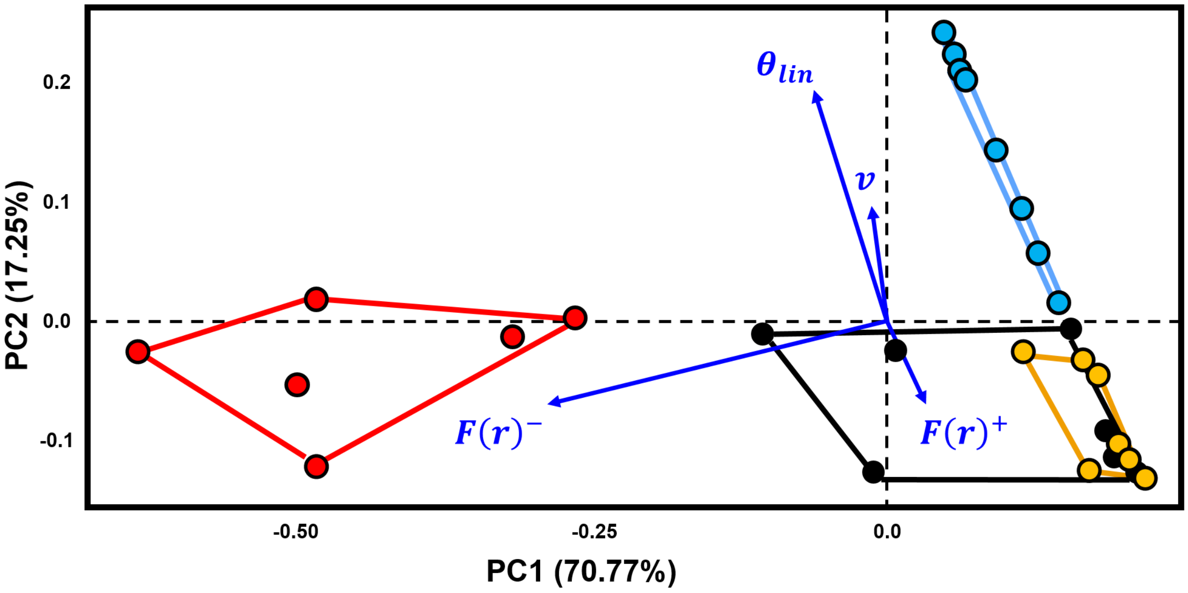

Supplementary Figure 80: *Principal Component Analysis biplot presenting convex hulls of all of the samples.*

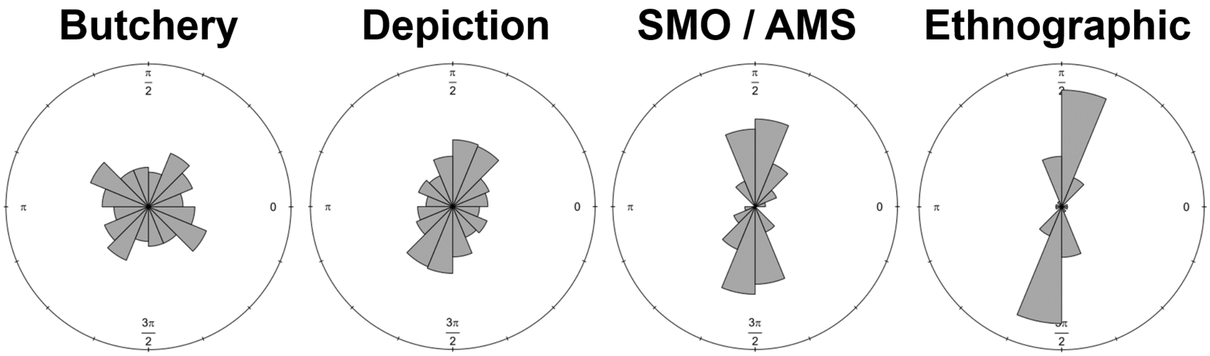

Supplementary Figure 81: *Rose diagrams comparing all of the angles from each of the samples.*

4 Appendix D: Supplementary Tables

Supplementary Table 1: *Statistical results for the quadrat tests of each of the studied bones. FPR = False Positive Risk, i.e. the probability (%) that the observed p-Value is a Type I statistical error with prior probabilities of 0.2, sensu Colquhoun (2019). FPR is represented as a percentage, while these values are only calculated for p-values under 0.3681, sensu Courtenay et al. (2021). An * indicates notable deviations from homogeneity.*

Case study	χ^2	p-Value	FPR
Experimental Defleshing	78.81	0.04	58.3
Experimental Disarticulation	48.321	0.001	6.99*
Experimental Tendon Removal	182.3	2×10^{-4}	1.82*
Lower Pleistocene Butchery	40.87	0.388	-
Middle Pleistocene Butchery	128.4	0.002	11.9*
Upper Pleistocene Butchery	21.60	0.025	50.1
Le Madeleine	141.30	2×10^{-4}	1.82*
La Marche Horse	678.88	2×10^{-4}	1.82*
Laugerie Basse Disc	184.95	2×10^{-4}	1.82*
Zigeunerhohle	267.67	2×10^{-4}	1.82*
Brassempouy	134.45	2×10^{-4}	1.82*
Isturitz 288	135.01	2×10^{-4}	1.82*
Isturitz 284	55.48	0.47	-
Duruthy	91.32	0.002	11.9*
Gough's Cave	733.75	2×10^{-4}	1.82*
Blanchard 1858	34.96	2×10^{-4}	1.82*
Solutré	98.95	7×10^{-4}	5.24*
Labattut	60.43	2×10^{-4}	1.82*
Les Pradelles	45.015	0.001	6.99*
Border Cave	28.016	0.002	11.9*
Tossal de la Roca Side 1	36.09	0.133	74.5
Tossal de la Roca Side 2	39.75	0.057	64.0
Blanchard 1958	54.37	2×10^{-4}	1.82*
La Marche AMS	674.58	2×10^{-4}	1.82*
Laugerie Basse Side 1	62.63	0.003	15.9*
Laugerie Basse Side 2	58.83	6×10^{-4}	4.6*
Winnebago	10.97	0.002	11.9*
Chamula	125.0	2×10^{-4}	1.82*
Aboriginal Australian	84.14	4×10^{-4}	3.29*
Medieval (English)	32.87	0.053	62.9
Medieval (Jewish)	1.500	0.313	79.8
Mirān	12.45	0.375	-
Muacapenda	40.72	0.048	61.3
Aboriginal Australian (2)	22.48	2×10^{-4}	1.82*
Muatchondo	4.64	0.001	6.70*

Supplementary Table 2: *A summary of all of the preferential orientation hypothesis tests computed for the present study. FPR = False Positive Risk, i.e. the probability that the observed p-Value is a Type I statistical error with prior probabilities of 0.2. FPR is represented as a percentage, while these values are only calculated for p-values under 0.3681, sensu Courtenay et al. (2021).*

Name	Sample	z	p	FPR
Defleshing	Butchery	0.958	$\ll 2.8 \times 10^{-16}$	$\ll 1.1 \times 10^{-11}$
Disarticulation	Butchery	0.984	$\ll 2.8 \times 10^{-16}$	$\ll 1.1 \times 10^{-11}$
Tendon Removal	Butchery	0.880	$\ll 2.8 \times 10^{-16}$	$\ll 1.1 \times 10^{-11}$
Lower Pleistocene	Butchery	0.945	9.0×10^{-4}	6.422
Middle Pleistocene	Butchery	0.981	$\ll 2.8 \times 10^{-16}$	$\ll 1.1 \times 10^{-11}$
Upper Pleistocene	Butchery	0.985	$\ll 2.8 \times 10^{-16}$	$\ll 1.1 \times 10^{-11}$
Le Madeleine	Depiction	0.900	$\ll 2.8 \times 10^{-16}$	$\ll 1.1 \times 10^{-11}$
La Marche Horse	Depiction	0.935	$\ll 2.8 \times 10^{-16}$	$\ll 1.1 \times 10^{-11}$
Laugerie Basse Disc	Depiction	0.917	$\ll 2.8 \times 10^{-16}$	$\ll 1.1 \times 10^{-11}$
Zigeunerhohle	Depiction	0.951	$\ll 2.8 \times 10^{-16}$	$\ll 1.1 \times 10^{-11}$
Brassempouy	Depiction	0.965	$\ll 2.8 \times 10^{-16}$	$\ll 1.1 \times 10^{-11}$
Isturitz 288	Depiction	0.982	$\ll 2.8 \times 10^{-16}$	$\ll 1.1 \times 10^{-11}$
Isturitz 284	Depiction	0.964	$\ll 2.8 \times 10^{-16}$	$\ll 1.1 \times 10^{-11}$
Duruthy	Depiction	0.973	$\ll 2.8 \times 10^{-16}$	$\ll 1.1 \times 10^{-11}$
Gough's Cave	Depiction	0.984	$\ll 2.8 \times 10^{-16}$	$\ll 1.1 \times 10^{-11}$
Blanchard 1858	AMS	1.000	$\ll 2.8 \times 10^{-16}$	$\ll 1.1 \times 10^{-11}$
Solutré	AMS	0.988	$\ll 2.8 \times 10^{-16}$	$\ll 1.1 \times 10^{-11}$
Labattut	AMS	0.999	$\ll 2.8 \times 10^{-16}$	$\ll 1.1 \times 10^{-11}$
Les Pradelles	AMS	0.984	$\ll 2.8 \times 10^{-16}$	$\ll 1.1 \times 10^{-11}$
Border Cave	AMS	0.995	$\ll 2.8 \times 10^{-16}$	$\ll 1.1 \times 10^{-11}$
Tossal de la Roca (A)	AMS	0.984	$\ll 2.8 \times 10^{-16}$	$\ll 1.1 \times 10^{-11}$
Tossal de la Roca (B)	AMS	0.960	$\ll 2.8 \times 10^{-16}$	$\ll 1.1 \times 10^{-11}$
Winnebago	Calendar	0.977	$\ll 2.8 \times 10^{-16}$	$\ll 1.1 \times 10^{-11}$
Chamula	Calendar	0.996	$\ll 2.8 \times 10^{-16}$	$\ll 1.1 \times 10^{-11}$
Aboriginal Australian	Message Stick	0.999	$\ll 2.8 \times 10^{-16}$	$\ll 1.1 \times 10^{-11}$
Medieval English	Tally Stick	0.996	$\ll 2.8 \times 10^{-16}$	$\ll 1.1 \times 10^{-11}$
Medieval Jewish	Tally Stick	0.999	$\ll 2.8 \times 10^{-16}$	$\ll 1.1 \times 10^{-11}$
Mirān	Tally Stick	0.999	$\ll 2.8 \times 10^{-16}$	$\ll 1.1 \times 10^{-11}$
Muacapenda	Tally Stick	0.999	$\ll 2.8 \times 10^{-16}$	$\ll 1.1 \times 10^{-11}$

Supplementary Table 3: *Randomised version of the Mardia-Watson-Wheeler test results to calculate whether two sets of angles have similar distributions. FPR = False Positive Risk, i.e. the probability that the observed p-Value is a Type I statistical error with prior probabilities of 0.2. FPR is represented as a percentage, while these values are only calculated for p-values under 0.3681, sensu Courtenay et al. (2021).*

Comparison	W_g	p	FPR
Butchery vs Depiction	34.25	3.7×10^{-8}	0.0007
Butchery vs Pleistocene SMO / AMS	77.2	1.8×10^{-17}	7.5×10^{-13}
Butchery vs Holocene SMO / AMS	254.3	5.9×10^{-56}	8.2×10^{-51}
Depiction vs Holocene SMO / AMS	531.3	4.2×10^{-116}	1.2×10^{-110}
Depiction vs Pleistocene SMO / AMS	144.3	4.6×10^{-32}	3.6×10^{-27}
Pleistocene SMO / AMS vs Holocene SMO / AMS	112.8	3.1×10^{-25}	1.9×10^{-20}

5 Appendix E: Validation of Clustering Results

As a means of validating our clustering results, and ensuring that inferences derived from analyses truly reflect patterns that are indicative of distinct groupings, we performed a number of simulations of synthetic artefacts to establish a base-line threshold of ideal results with which to compare our empirical data. For this purpose, we propose 3 ideal models containing both spatial (Point Pattern Processes - PPPs) and orientation data presenting the following features;

- **Ideal Scenario 1** - PPPs presenting Complete Spatial Randomness (CSR), with no preferential orientation at all.
- **Ideal Scenario 2** - PPPs presenting clustering patterns, with a slight preferential orientation oblique to the main axis of the spatial window.
- **Ideal Scenario 3** - PPPs presenting a regular spatial distribtuion, with a clear preferential orientation perpendicular to the main axis of the spatial window.

These would reflect ideal situations where all three sets are truly distinct from eachother, and approximate our current understanding of butchery, depictional representations, and the potential AMSs respectively. We can model these three ideal scenarios in a spatial window of a fixed unitless size $[0, 1] \in \mathbb{R}^2$, by defining the following models;

- **Model 1** - Simulate n number of x and y coordinates within the spatial window by sampling from a Poisson distribution to produce randomly distributed points fulfilling the properties of CSR. For angles, sample n values from a Von Mises distribution, with a μ_θ of π, and a very low κ valu of 0.5, emulating the complete lack of a preferential orientation of notches.
- **Model 2** - Simulate $\lfloor {}^{n}/_{g} \rfloor$ number of x and y coordinates by computing a random number (g) of multivariate Gaussian distributions with σ values of 0.05, and centered around g distinct points at least more than 2σ away from each other, so as to create g number of synthetic clusters. For orientation values, we sample n values from a Von Mises distribution with a μ_θ value of $\pi - 1$, and a moderate κ value of 5, so as to generate oblique angles with a moderate preferential orientation.
- **Ideal Scenario 3** - Simulate n number of x and y coordinates by creating a grid of equally spaced points, introducing a minute perturbation with $\sigma = 0.01$ to introduce a stochastic nature to an already regularly spaced PPP. For orientation values, we sample from a Von Mises distribution with a μ_θ of ${}^{\pi}/_{2}$ with an extreme κ value of 20.

For all models, n was set to 100 and g to 4 for model simplicity, also considering most of the depiction and potential AMS artefacts to display at least 100 different markings, while most butchery samples also showed at least 4 different clusters with the exception of the FLK-West bone in Sup. Fig. 4. A visualisation of the three ideal hypothetical models are presented in Sup. Fig. 82.

These models were used to simulate a number of artificial artefacts, respecting the associated sample sizes and sample balance of our actual empirical samples, so as to avoid potential issues due to the Marčenko-Pastur theorem (Marčenko and Pastur, 1967). Once these different synthetic artefacts had been simulated, we proceeded to calculate $F(r)$ curves with their associated Area Under the Poisson Curve (AUPC) and Area Over the Poisson Curve (AOPC) values, as well as the θ_{lin} and v values from the simulated angles. PCAs were then calculated from our set of simulated individuals, and from the corresponding PC scores we calculated hierarchical clustering results. An example of the resulting PCA feature space and clustering results for the three theoretical models is presented in Sup. Fig. 83.

For the selection of the clustering algorithm, we used the Unweighted Pair Group Method with Arithmetic Mean (UPGMA) method (Sokal and Michener, 1958). This was chosen as UPGMA better suits our clustering requirements and data structure, while it was found to converge on the same results and identify the same splits between individual points 73.9 $\pm$ 16.0 % of the time when bootstrapped for 1000 iterations. The other alternatives that can considered are; single and complete linkage methods, which were only found to identify the same splits between individuals 45.9 $\pm$ 17.4 % of the time; two variants of Ward's minimum variance method (Ward I = 51.1 $\pm$ 17.0 %; Ward II = 50.8 $\pm$ 16.0 %) (Ward, 1963; Murtapgh and Legendre, 2014); the Weighted Group Method with Arithmetic Mean (WGPMA; 50.5 $\pm$ 16.0 %) (McQuitty, 1966); as well as the variants of UPGMA and WPGMA that utilise centroids insted of the mean to compute clusters (UPGMC = 50.3 $\pm$ 16.9 %; WPGMC = 50.7 $\pm$ 16.6 %).

Once the dendograms had been established, clustering results were then evaluated by means of the cophenetic correlation coefficient and Ajusted Mututal Information (AMI) criterion, as explained in the methods section of the main text of our study.

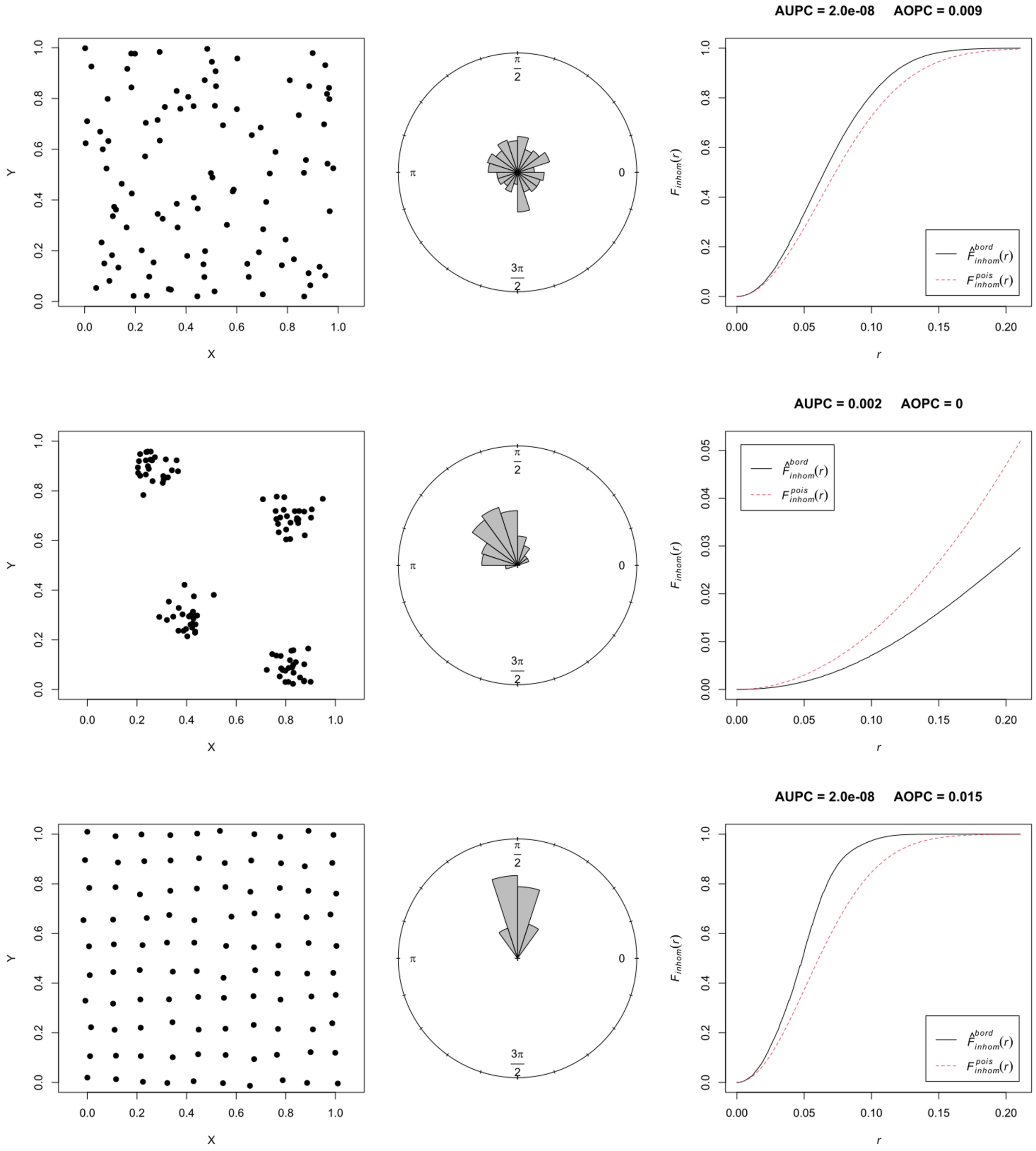

Supplementary Figure 82: *Examples of the three ideal theoretical models used for simulation purposes in the present study*

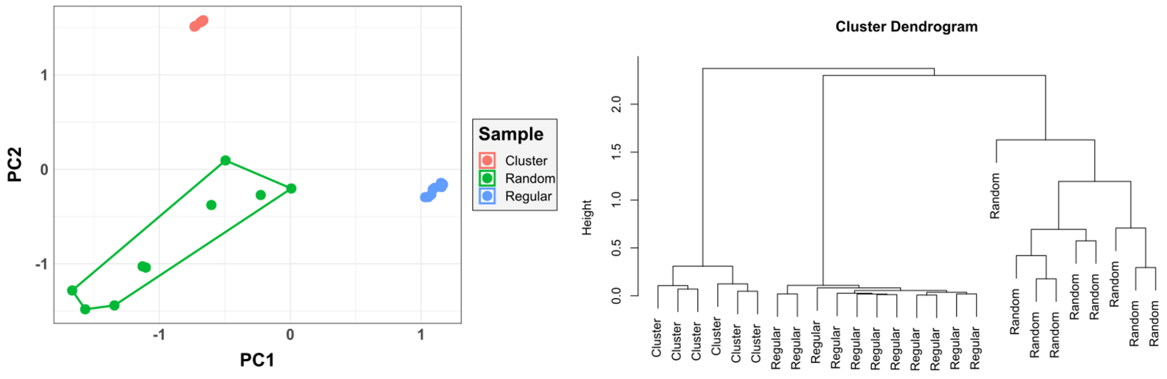

Supplementary Figure 83: *Examples of the PCA and clustering results performed on the three ideal models of samples.*

Following the definition of the three ideal models, experiments were then performed gradually adjusting the signal of these three ideal simulations to impose a greater degree of overlap between the three models. This was performed by gradually introducing points from other distributions into the ideal samples, or by decreasing the strength of the original model's signal by playing with σ or κ values. An example of this can be visualised in Sup. Fig. 84 with the resulting PCA and clustering results visualised in Sup. Fig. 85

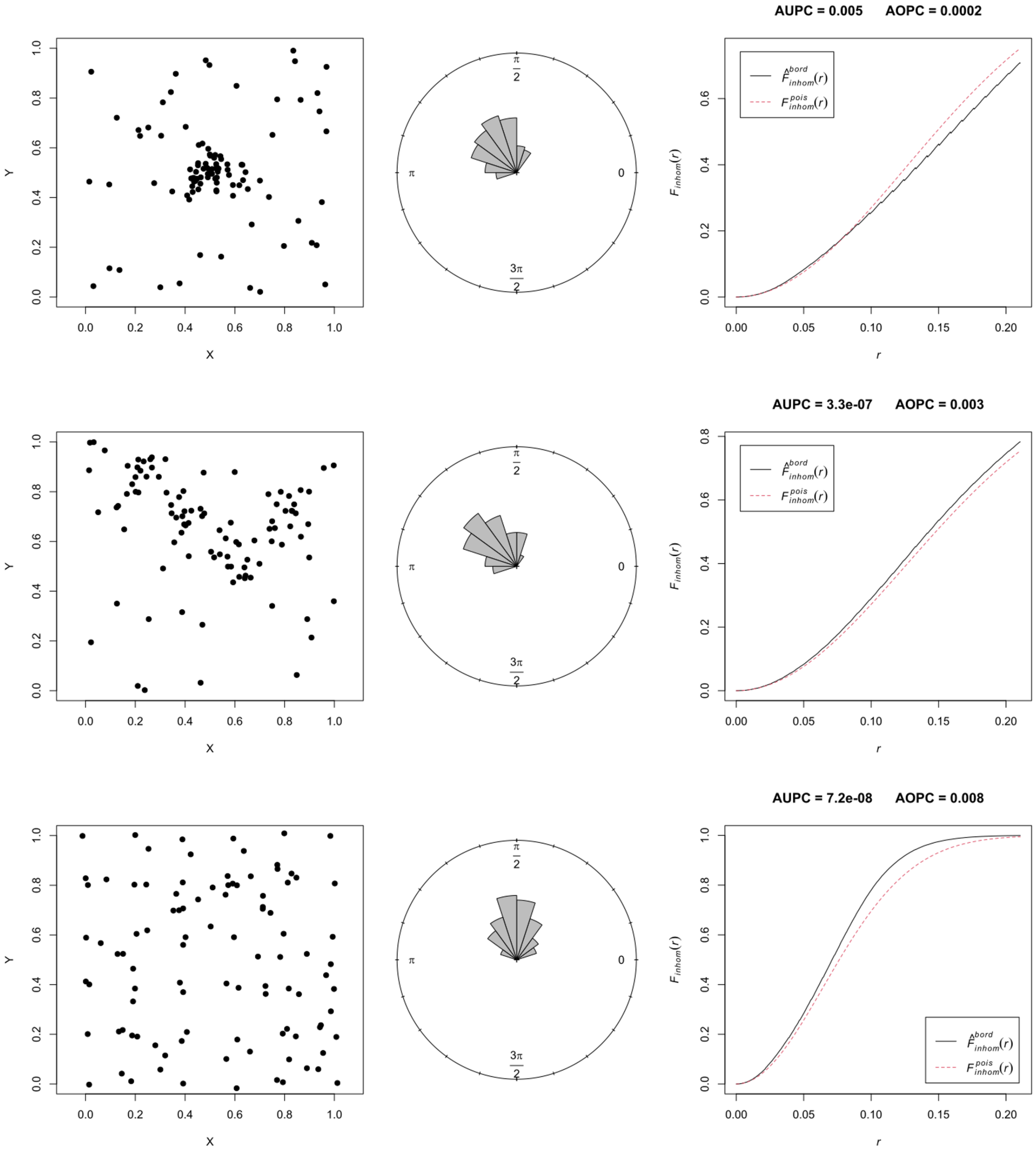

Supplementary Figure 84: *Examples of three contaminated theoretical distributions. The first example is taking model 1, whereby points are randomly distributed, with the added inclusion of a single strong cluster with* $\mu = [0, 0]$ *,* $\sigma = [0.05, 0.05]$*. Orientation values for this model maintain the original* μ_θ *value of model 1, however increase* κ *to 5. The second example is taking model 2, and introducing a number of randomly distributed points sampled from a Poisson distribution as well. In this case the Von Mises distribution is not modified from the original model. The final example is of model 3, with the addition of some random points from a Poisson distribution as well. The Von Mises distribution in this last case maintains the same* μ_θ *value, however decreases* κ *to 5*

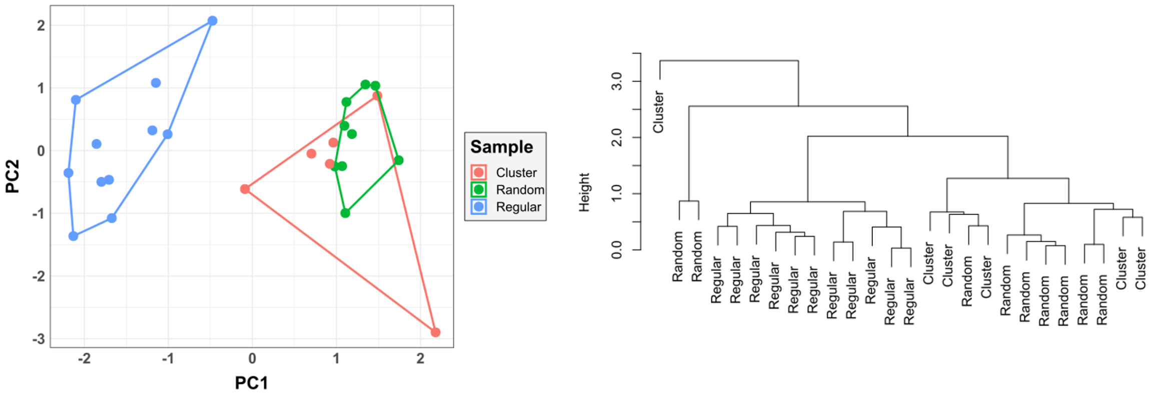

Supplementary Figure 85: *Examples of simulated models presenting certain degrees of overlap after contamination with other spatial distributions*

Finally, to establish a simulation that can be used to fulfill the criterion of falsifiability, we establish three groups that are completely random, with all 3 models sampling from a Poisson distribution for spatial points, while angles are sampled from a Von Mises distribution with a μ_θ of π and a κ of 0.01, resulting in the following PCA and clustering results:

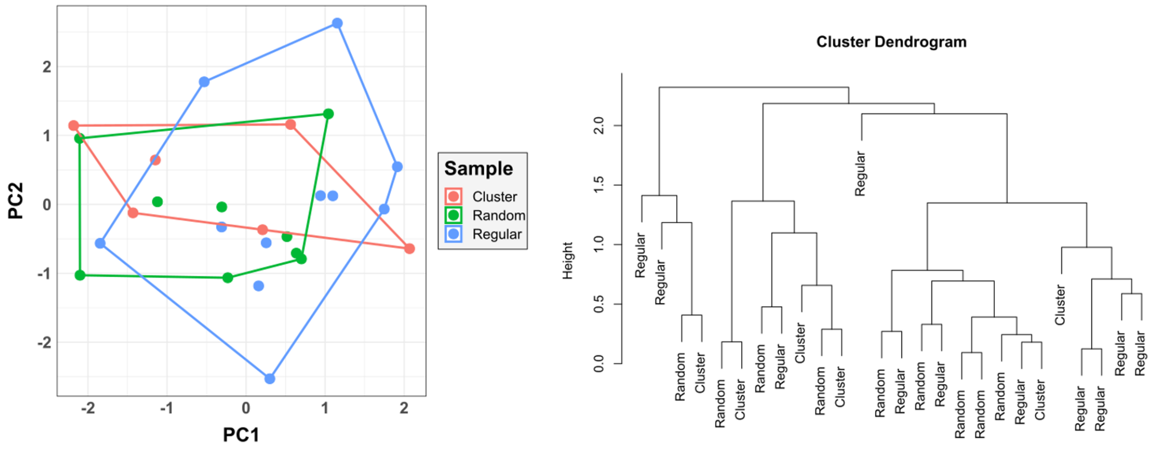

Supplementary Figure 86: *Examples of simulated models presenting no separation between groups*

To evaluate the degree of overlap and separation between these hypothetical samples, and evaluate the dendograms derived from them, we considered two metrics; (1) the area of overlap between the convex hulls; (2) the euclidean distance between robustly calculated group centroids (utilising median coordinates instead of the mean). Our first simulated observations showed that after a certain distance, groups were not found to present any degree of overlap, calculated at approximately 0.48 units (Sup. Fig. 87). Likewise, the area of overlap metric was not found to correlate well with either the cophenetic correlation (ρ = -0.31, t = -0.75, p = 0.49), or the AMI (ρ = -0.87, t = -3.97, p = 0.01, FPR = 11.6%). From this perspective, we can safely assume that the area of overlap is only useful in these simulations to indicate whether overlap exists or not, as a simple boolean variable with euclidean distance values above 0.48 indicating no overlap between our theoretical groups. Euclidean distance between centroids, however, did show a notable relationship with both the cophenetic correlation (ρ = 0.82, t = 4.59, p = 0.001, FPR = 1.8%) and the AMI (ρ = 0.97, t = 13.1, $p = 1.3 \times 10^{-7}$, FPR = 0.0006 %), indicating this metric to be much more informative beyond simple observations of whether overlap exists.

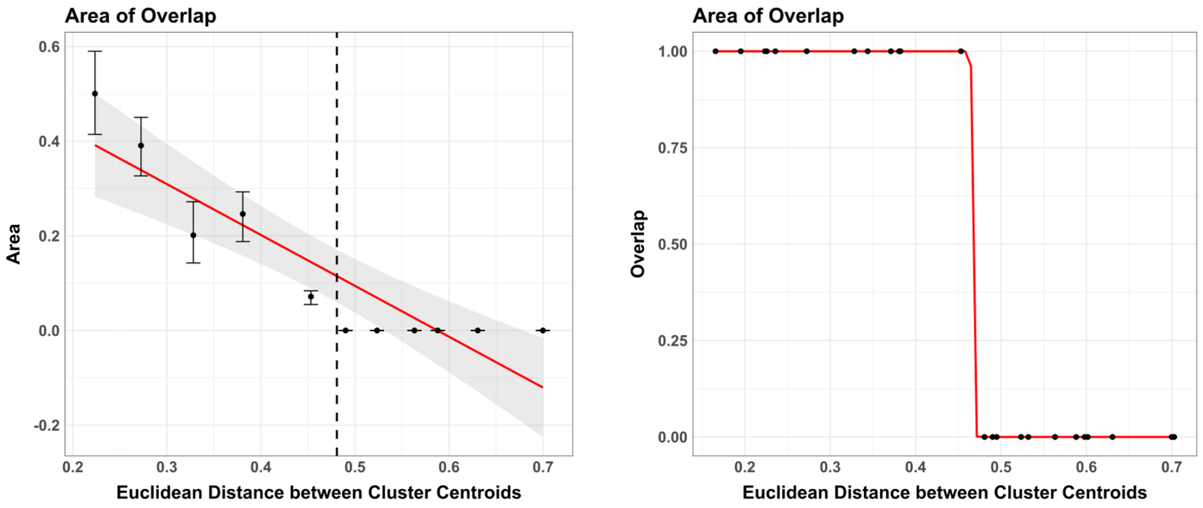

Supplementary Figure 87: *(Left) Simulated error-bar plots indicating the relationship between the two metrics used to evaluate the theoretical difference between different variants of our theoretical models. Error bars represent 95% bootstrapped (*$\times 1000$*) confidence intervals. (Right) A simple logistic regression plot remonstrating the binary relationship of the area of overlap metric, with values above 0.48 indicating no overlap, while areas below indicate overlap, regardless of how much overlap is present.*

From this perspective, it can now be seen how the moment overlap ceases to exist between groups, a linear relationship is present between the cophenetic correlation coefficient and the euclidean distance between groups, with correlation coefficients ranging between approximately 0.82 and 0.96, with a median of 0.91 and a Square Root of the Biweight Midvariance ($\sqrt{BWMV}$) of 0.05. When overlap does exist, the cophenetic correlation coefficient tends to fall within a range of 0.70 and 0.82, with a median of 0.77 and $\sqrt{BWMV}$ of 0.02. Our empirical results from the main study presented values between 0.8903 and 0.8942, falling clearly within the range of values that would be expected if the three theoretical groups with no-overlap exist. When considering AMI values, a linear relationship is generally present across values both above and bellow the 0.48 threshold indicating overlap. In this sense AMI values typically fall between the range of -0.02 and 0.60, with a median of 0.10 and $\sqrt{BWMV}$ of 0.20 when overlap exists, and a range of 0.45 and 1, with a median of 0.63 and $\sqrt{BWMV}$ of 0.20, when no overlap exists. Once again, considering our empirical AMI results fall between a range of 0.68 and 0.72, this is indicative that the theoretically separate and distinct models are more likely to exist.

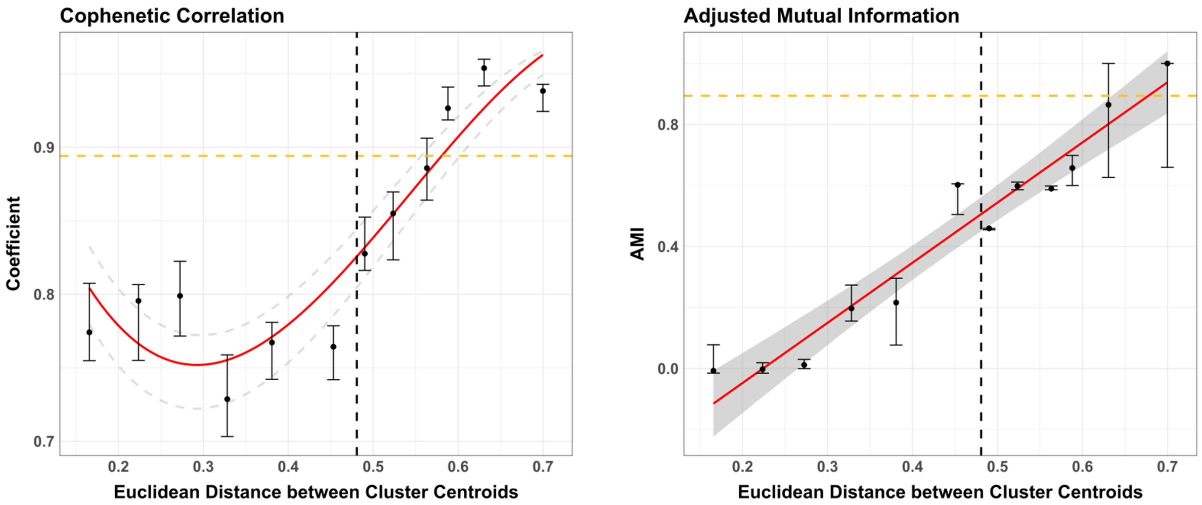

Supplementary Figure 88: *(Left) Simulated error-bar plots indicating the relationship between the euclidean distance between theoretical group centroids, and the cophenetic correlation evaluation metric of corresponding hierarchical clustering results. (Right) Simulated error-bar plots indicating the relationship between the euclidean distance between theoretical group centroids, and the Adjusted Mutual Information criterion evaluation metric of corresponding hierarchical clustering results. For both plots error bars represent 95% bootstrapped ($\times 1000$) confidence intervals. The orange horizontal line indicates the position of our empirical data, while the black vertical line indicates the previously defined threshold where overlap is observed between theoretical samples or not.*

References

Bello, S.M.; Wallduck, R.; Parfitt, S.A.; and Stringer, C.B. (2017) An Upper Palaeolithic engraved human bone associated with ritualistic cannibalism, *PLoS ONE*, 12(8):e0182127, doi:10.1371/journal.pone.0182127.

Colquhoun, D. (2019) The False Positive Risk: a proposal concerning what to do about *p*-values, *The American Statistician*, 73(Sup1):192–201, doi:10.1080/00031305.2018.1529622.

Costamagno, S.; Soulier, M.C.; Val, A.; and Chong, S. (2018) Le référentiel de stries de boucherie, in: C. Thiébaut; E. Claud; and S. Costamagno (Eds.) *L'aquisition et le traitement des matières végétales et animales par les Néandertaliens: quelles modalités et quelles stratégies?*, Online: Palethnologie, 195–291, doi:10.400/palethnologie.3548.

Courtenay, L.A. (2019) *New methodological advances in the study of taphonomic equifinality in the Lower Pleistocene Site of FLK-West (Olduvai Gorge, Tanzania)*, Masters thesis, Universitat Rovira i Virgili, Tarragona, Spain.

Courtenay, L.A.; González-Aguilera, D.; Lagüela, S.; del Pozo, S.; Ruiz-Mendez, C.; Barbero-García, I.; Román-Curto, C.; Cañueto, J.; Santos-Durán, C.; Cardeñoso-Álvarez, M.E.; Roncero-Riesco, M.; Hernandez-Lopez, D.; Guerrero-Sevilla, D.; and Rodríguez-Gonzalvez, P. (2021) Hyperspectral imaging and robust statistics in non-melanoma skin cancer analysis, *Biomedical Optics Express*, 12(8):5107–5127, doi:10.1364/BOE.428143.

D'Errico, F. (1995) A new model and its implications for the origin of writing: the La Marche antler revisited, *Cambridge Archaeological Journal*, 5(2):163–206.

D'Errico, F. (1998) Palaeolithic origins of artifical memory systems: an evolutionary perspective, in: C. Renfrew and C. Scarre (Eds.) *Cognition and Material Culture: the Archaeology of Symbolic Storage*, Cambridge: McDonald Institute Monographs, 19–50.

D'Errico, F. and Cacho, C. (1994) Notation versus decoration in the Upper Palaeolithic: a case-study from Tossal de la Roca, Alicante, Spain, *Journal of Archaeological Science*, 21:185–200.

D'Errico, F.; Doyon, L.; Colagé, I.; Queffelec, A.; Le Vraux, E.; Giacobini, G.; Vandermeersch, B.; and Maureille, B. (2017) From number sense to number symbols. an archaeological perspective, *Philosophical Transactions of the Royal Society B*, 373:20160518, doi:10.1098/rstb.2016.0518.

Gossen, G.H. (1972) A Chamula callendar board from Chiapas, Mexico, in: N. Hammond (Ed.) *Mesoamerican Archaeology: New Approaches*, Austin: University of Texas Press, 217–254.

Hardy, M. (1868) Les habitants préhistoriques de cavernes, *La Magasin Pittoreque*, 1:359–363.

Howitt, A.W. (1904) *The native tribes of South-east Australia*, London: Macmillan, 691–710.

Ifrah, G. (1985) *From one to zero: a universal history of numbers*, Virginia: Penguin Books.

Jenkinson, H. (1925) Medieval tallies, public and private, *Archaeologia*, 74:289–351, doi:10.1017/S0261340900013175.

Lagercrantz, S. (1973) Counting by means of tally sticks or cuts on the body in Africa, *Anthropos*, 68:569–588.

López Cisneros, P. (2020) *Patrones culturales en los procesos de carnicería sobre los macrovertebrados en el Paleolítico Superior Cantábrico*, Phd thesis, Complutense University of Madrid, Madrid, Spain.

Lyell, C. (1873) *The geological evidences of the Antiquity of Man*, London: Murray.

Marshack, A. (1972) The Chamula calendar board: an internal and comparative analysis, in: N. Hammond (Ed.) *Mesoamerican Archaeology: New Approaches*, Austin: University of Texas Press, 256–270.

Marshack, A. (1985) A lunar-solar year calendar stick from North America, *American Antiquity*, 50(1):27–51.

Marshack, A. (1987) The evolution and transformation of "decoration": early aurignacian to the terminal magdalenian, in: J. Clottes (Ed.) *Colloque International d'Art Mobilier Paléolithique*, Harvard: Peabody Museum, 1–32.

Marshack, A. (1988) North American Indian calendar sticks: the evidence for a widely distributed tradition, in: A.F. Aveni (Ed.) *World Archaeoastronomy*, Cambridge: Cambridge University Press, 308–324.

Marčenko, V. and Pastur, L. (1967) Distributions of eigenvalues for some sets of random matrices, *Mathematics of the USSR Sbornik*, 1:457–483.

McQuitty, L. (1966) Similarity analysis by recipocal pairs for discrete and continuous data, *Educational and Psychological Measurement*, 26:825–831.

Murtapgh, F. and Legendre, P. (2014) Ward's hierarchical agglomerative clustering method: which algorithms implement ward's criterion, *Journal of Classification*, 31:274–295.

Rodríguez-Hidalgo, A.; Saldié, P.; Ollè, A.; and Carbonell, E. (2015) Hominin subsistence and site funciton of TD10.1 bone bed level at Gran Dolina site (Atapuerca) during the late Acheulean, *Journal of Quaternary Science*, 30(7):679–701.

Sokal, R. and Michener, C. (1958) *A statistical method for evaluating systematic relationships*, Kansas: University of Kansas Science Bulletin.

Tas, A.R. (1956) Tally-stick and divination-dice in the iconography of Lha-Mo, *Acta Orientalia Acaemiae Scientiarum Hungaricae*, 6:163–179.

Ward, J. (1963) Hierarchical grouping to optimize an objective function, *Journal of the American Statistical Association*, 58:236–244.
